# Supplementary material for: Quantitative Measures of Physical Risk Factors Associated with Work-Related Musculoskeletal Disorders of the Elbow: A Systematic Review
Source: Int J Environ Res Public Health. 2019 Jan 5;16(1):130. doi: 10.3390/ijerph16010130 (PMC6339038; doi:10.3390/ijerph16010130)
Supplement: Supplementary file 1 [file ijerph-16-00130-s001.pdf]

Review – Supplementary Files (SF)

Quantitative Measures of Physical Risk Factors Associated with Work-Related Musculoskeletal Disorders of the Elbow: A Systematic Review

David H. Seidel, Dirk M. Ditchen, Ulrike M. Hoehne-Hückstädt, Monika A. Rieger and Benjamin Steinhilber

File I – Table S1: PRISMA checklist adopted from Moher et al. [12] and modified

Table S1. PRISMA checklist adopted from Moher et al. [12] and modified.

Section/topic	Item No.	Checklist item	Section	Page
**TITLE**				
Title	1	Identify the report as a systematic review, meta-analysis, or both.	Title page	1
**ABSTRACT**				
Structured summary	2	Provide a structured summary including, as applicable: background; objectives; data sources; study eligibility criteria, participants, and interventions; study appraisal and synthesis methods; results; limitations; conclusions and implications of key findings; systematic review registration number.	Abstract	1
**INTRODUCTION**				
Rationale	3	Describe the rationale for the review in the context of what is already known.	Introduction	1, 2
Objectives	4	Provide an explicit statement of questions being addressed with reference to participants, interventions, comparisons, outcomes, and study design (PICOS).	Methods	4
**METHODS**				
Protocol and registration	5	Indicate whether a review protocol exists, if and where it can be accessed (e.g., Web address), and, if available, provide registration information including registration number.	not exist	-
Eligibility criteria	6	Specify study characteristics (e.g., PICOS, length of follow-up) and report characteristics (e.g., years considered, language, publication status) used as criteria for eligibility, giving rationale.	Methods	1 to 4
Information sources	7	Describe all information sources (e.g., databases with dates of coverage, contact with study authors to identify additional studies) in the search and date last searched.	Methods & Supplementary Files	2, SF 5 to SF 8

Search	8	Present full electronic search strategy for at least one database, including any limits used, such that it could be repeated.	Supplementary Files	SF 5 to SF 8
Study selection	9	State the process for selecting studies (i.e., screening, eligibility, included in systematic review, and, if applicable, included in the meta-analysis).	Methods & Supplementary Files	2, 3, SF 9 to SF 50
Data collection process	10	Describe method of data extraction from reports (e.g., piloted forms, independently, in duplicate) and any processes for obtaining and confirming data from investigators.	Methods	2 to 4
Data items	11	List and define all variables for which data were sought (e.g., PICOS, funding sources) and any assumptions and simplifications made.	Methods, Supplementary Files	2 to 4, SF 5 to SF 8
Risk of bias in individual studies	12	Describe methods used for assessing risk of bias of individual studies (including specification of whether this was done at the study or outcome level), and how this information is to be used in any data synthesis.	Methods	5
Summary measures	13	State the principal summary measures (e.g., risk ratio, difference in means).	Methods	5
Synthesis of results	14	Describe the methods of handling data and combining results of studies, if done, including measures of consistency (e.g., I^2) for each meta-analysis.	Methods	5
Risk of bias across studies	15	Specify any assessment of risk of bias that may affect the cumulative evidence (e.g., publication bias, selective reporting within studies).	Methods, Results	5, 14 to 15
Additional analyses	16	Describe methods of additional analyses (e.g., sensitivity or subgroup analyses, meta-regression), if done, indicating which were pre-specified.	not exist	-
**RESULTS**				
Study selection	17	Give numbers of studies screened, assessed for eligibility, and included in the review, with reasons for exclusions at each stage, ideally with a flow diagram.	Results	3
Study characteristics	18	For each study, present characteristics for which data were extracted (e.g., study size, PICOS, follow-up period) and provide the citations.	Supplementary Files	SF 50 to SF 59
Risk of bias within studies	19	Present data on risk of bias of each study and, if available, any outcome level assessment (see item 12).	(grouped) Results	5, 14 to 15

Results of individual studies	20	For all outcomes considered (benefits or harms), present, for each study: (a) simple summary data for each intervention group (b) effect estimates and confidence intervals, ideally with a forest plot.	Results, Supplementary Files	6 to 14, SF 9 to SF 65
Synthesis of results	21	Present the main results of the review. If meta-analyses are done, include for each, confidence intervals and measures of consistency.	Results, Supplementary Files	6 to 14, SF 9 to SF 65
Risk of bias across studies	22	Present results of any assessment of risk of bias across studies (see Item 15).	Results	14, 15
Additional analysis	23	Give results of additional analyses, if done (e.g., sensitivity or subgroup analyses, meta-regression [see Item 16]).	not exist	-
**DISCUSSION**				
Summary of evidence	24	Summarize the main findings including the strength of evidence for each main outcome; consider their relevance to key groups (e.g., healthcare providers, users, and policy makers).	Discussion	16 to 19
Limitations	25	Discuss limitations at study and outcome level (e.g., risk of bias), and at review-level (e.g., incomplete retrieval of identified research, reporting bias).	Discussion	19, 20
Conclusions	26	Provide a general interpretation of the results in the context of other evidence, and implications for future research.	Discussion	21
**FUNDING**				
Funding	27	Describe sources of funding for the systematic review and other support (e.g., supply of data); role of funders for the systematic review.	Discussion	22

File II – Table S2: MEDLINE search strategy – 03th February 2017

Table S2. MEDLINE search strategy – 03th February 2017.

No.	Searches	Reference/ Comment	Results
I	(elbow [MH] OR upper limb [MH] OR upper extremit*[MH] elbow joint [MeSH Terms] OR cubital joint OR articulatio cubiti)		18738
II	(musculoskeletal disorders OR pain OR injury OR cumulative trauma disorders)	[4]	2535768
III	**#1 AND #2**		**13842**
IV	(epicondylitis OR (epicondylitides, lateral humeral[MeSH Terms]) OR (epicondylitis, lateral humeral[MeSH Terms]) OR (humeral epicondylitides, lateral[MeSH Terms]) OR (tennis* AND elbow) OR (golf* AND elbow) OR cubital tunnel syndrome OR radial tunnel syndrome OR ulnar nerve entrapment OR median nerve entrapment OR pronator teres syndrome OR tenosynovitis OR tendovaginitis OR radial nerve entrapment)	[6]	17373
V	#3 OR #4		30227
VI	(((((((("Elbow/abnormalities"[MeSH] OR "Elbow/diagnosis"[MeSH] OR "Elbow/etiology"[MeSH] OR "Elbow/injuries"[MeSH] OR "Elbow/pathology"[MeSH] OR "Elbow/physiopathology"[MeSH]))) OR (("Elbow Joint/abnormalities"[MeSH] OR "Elbow Joint/diagnosis"[MeSH] OR "Elbow Joint/etiology"[MeSH] OR "Elbow Joint/injuries"[MeSH] OR "Elbow Joint/pathology"[MeSH] OR "Elbow Joint/physiopathology"[MeSH]))) OR (("Tennis Elbow/diagnosis"[MeSH] OR "Tennis Elbow/epidemiology"[MeSH] OR "Tennis Elbow/etiology"[MeSH] OR "Tennis Elbow/mortality"[MeSH] OR "Tennis Elbow/pathology"[MeSH] OR "Tennis Elbow/physiopathology"[MeSH]))) OR (("Elbow Tendinopathy/diagnosis"[MeSH] OR "Elbow Tendinopathy/epidemiology"[MeSH] OR "Elbow Tendinopathy/etiology"[MeSH] OR "Elbow Tendinopathy/mortality"[MeSH] OR "Elbow Tendinopathy/pathology"[MeSH] OR "Elbow Tendinopathy/physiopathology"[MeSH]))) OR (("Cubital Tunnel Syndrome/diagnosis"[MeSH] OR "Cubital Tunnel Syndrome/epidemiology"[MeSH] OR "Cubital Tunnel Syndrome/etiology"[MeSH] OR "Cubital Tunnel Syndrome/pathology"[MeSH] OR "Cubital Tunnel Syndrome/physiopathology"[MeSH]))))		8987
VII	**#5 OR #6**		32111
VIII	((((((epicondylitides, lateral humeral[MeSH Terms]) OR epicondylitis, lateral humeral[MeSH Terms]) OR humeral epicondylitides, lateral[MeSH Terms]) OR humeral epicondylitis, lateral[MeSH Terms]) OR lateral humeral epicondylitides[MeSH Terms]) OR lateral humeral epicondylitis[MeSH Terms])		1438

IX	(((((((carpal tunnel syndrome[MeSH Terms]) OR carpal tunnel syndromes[MeSH Terms]) OR compression neuropathy, carpal tunnel[MeSH Terms]) OR entrapment neuropathy, carpal tunnel[MeSH Terms]) OR median neuropathy, carpal tunnel[MeSH Terms]) OR syndrome, carpal tunnel[MeSH Terms]) OR syndromes, carpal tunnel[MeSH Terms])		7605
X	((rotator cuff[MeSH Terms]) OR cuff, rotator[MeSH Terms])		5148
XI	**#8 AND #9**		51
XII	**#8 AND #10**		10
XIII	**#7 NOT #9 NOT #10**		23920
XIV	**#13 OR #11 OR #12**		23981
XV	(occupational diseases [MH] OR occupational exposure [MH] OR occupational exposure* [TW] OR "occupational health" OR "occupational medicine" OR work-related OR working environment [TW] OR at work [TW] OR work environment [TW] OR occupations [MH] OR work [MH] OR workplace* [TW] OR workload OR occupation* OR worke* OR work place* [TW] OR work site* [TW] OR job* [TW] OR occupational groups [MH] OR employment OR worksite* OR industry OR risk factor* OR occupational risk [TW])	[13] (p. 437) <u>added:</u> OR risk factor* OR occupational risk [TW] OSH-Terms	2164104
XVI	**#14 AND #15**		1904
XVII	("2007/09/01"[Date - Publication] : "2017/02/01"[Date - Publication])		8875059
XVIII	**#16 AND #17**		848
XIX	(("Humans"[MeSH Terms]) AND "Adult"[MeSH Terms])		6159838
XX	**#18 AND #19**		490
XXI	(((TH[SH] OR (Case Reports[Publication Type]) OR child [MH] OR child* [OT] OR child* [Title])))	no therapies / case reports	8244510
XXII	**#20 NOT #21**		159
XXIII	Filters: Abstract; Full text; German; English		141

File III – MEDLINE search string – 03th February 2017

((((((((((((((((((((((elbow [MH] OR upper limb [MH] OR upper extremit*[MH] elbow joint [MeSH Terms] OR cubital joint OR articulatio cubiti))) AND ((musculoskeletal disorders OR pain OR injury OR cumulative trauma disorders)))) OR ((epicondylitis OR (epicondylitides, lateral humeral[MeSH Terms]) OR (epicondylitis, lateral humeral[MeSH Terms]) OR (humeral epicondylitides, lateral[MeSH Terms]) OR (tennis* AND elbow) OR (golf* AND elbow) OR cubital tunnel syndrome OR radial tunnel syndrome OR ulnar nerve entrapment OR median nerve entrapment OR pronator teres syndrome OR tenosynovitis OR tendovaginitis OR radial nerve entrapment)))) OR (((((((("Elbow/abnormalities"[MeSH] OR "Elbow/diagnosis"[MeSH] OR "Elbow/etiology"[MeSH] OR "Elbow/injuries"[MeSH] OR "Elbow/pathology"[MeSH] OR "Elbow/physiopathology"[MeSH]))) OR (("Elbow Joint/abnormalities"[MeSH] OR "Elbow Joint/diagnosis"[MeSH] OR "Elbow Joint/etiology"[MeSH] OR "Elbow Joint/injuries"[MeSH] OR "Elbow Joint/pathology"[MeSH] OR "Elbow Joint/physiopathology"[MeSH]))) OR (("Tennis Elbow/diagnosis"[MeSH] OR "Tennis Elbow/epidemiology"[MeSH] OR "Tennis Elbow/etiology"[MeSH] OR "Tennis Elbow/mortality"[MeSH] OR "Tennis Elbow/pathology"[MeSH] OR "Tennis Elbow/physiopathology"[MeSH]))) OR (("Elbow Tendinopathy/diagnosis"[MeSH] OR "Elbow Tendinopathy/epidemiology"[MeSH] OR "Elbow Tendinopathy/etiology"[MeSH] OR "Elbow Tendinopathy/mortality"[MeSH] OR "Elbow Tendinopathy/pathology"[MeSH] OR "Elbow Tendinopathy/physiopathology"[MeSH]))) OR (("Cubital Tunnel Syndrome/diagnosis"[MeSH] OR "Cubital Tunnel Syndrome/epidemiology"[MeSH] OR "Cubital Tunnel Syndrome/etiology"[MeSH] OR "Cubital Tunnel Syndrome/pathology"[MeSH] OR "Cubital Tunnel Syndrome/physiopathology"[MeSH]))))))) NOT (((((((carpal tunnel syndrome[MeSH Terms]) OR carpal tunnel syndromes[MeSH Terms]) OR compression neuropathy, carpal tunnel[MeSH Terms]) OR entrapment neuropathy, carpal tunnel[MeSH Terms]) OR median neuropathy, carpal tunnel[MeSH Terms]) OR syndrome, carpal tunnel[MeSH Terms]) OR syndromes, carpal tunnel[MeSH Terms]))) NOT (((rotator cuff[MeSH Terms]) OR cuff, rotator[MeSH Terms])))) OR ((((((((epicondylitides, lateral humeral[MeSH Terms]) OR epicondylitis, lateral humeral[MeSH Terms]) OR humeral epicondylitides, lateral[MeSH Terms]) OR humeral epicondylitis, lateral[MeSH Terms]) OR lateral humeral epicondylitides[MeSH Terms]) OR lateral humeral epicondylitis[MeSH Terms]))) AND (((((((carpal tunnel syndrome[MeSH Terms]) OR carpal tunnel syndromes[MeSH Terms]) OR compression neuropathy, carpal tunnel[MeSH Terms]) OR entrapment neuropathy, carpal tunnel[MeSH Terms]) OR median neuropathy, carpal tunnel[MeSH Terms]) OR syndrome, carpal tunnel[MeSH Terms]) OR syndromes, carpal tunnel[MeSH Terms])))) OR ((((((((epicondylitides, lateral humeral[MeSH Terms]) OR epicondylitis, lateral humeral[MeSH Terms]) OR humeral epicondylitides, lateral[MeSH Terms]) OR humeral epicondylitis, lateral[MeSH Terms]) OR lateral humeral epicondylitides[MeSH Terms]) OR lateral humeral epicondylitis[MeSH Terms]))) AND (((rotator cuff[MeSH Terms]) OR cuff, rotator[MeSH Terms]))))) AND ((occupational diseases [MH] OR occupational exposure [MH] OR occupational exposure* [TW] OR “occupational health” OR “occupational medicine” OR work-related OR working environment [TW] OR at work [TW] OR work environment [TW] OR occupations [MH] OR work [MH] OR workplace* [TW] OR workload OR occupation* OR worke* OR work place* [TW] OR work site* [TW] OR job* [TW] OR occupational groups [MH] OR employment OR worksite* OR industry OR risk factor* OR occupational risk [TW])))) AND ("2007/09/01"[Date - Publication] : "2017/02/01"[Date - Publication]))) AND ((("Humans"[MeSH Terms]) AND "Adult"[MeSH Terms])))) NOT ((((TH[SH] OR (Case Reports[Publication Type]) OR child [MH] OR child* [OT] OR child* [Title]))))) AND ((hasabstract[text] AND full text[sb]) AND (English[lang] OR German[lang])))

File IV – Table S3: EMBASE search strategy – 08th February 2017

Table S3. EMBASE search strategy – 08th February 2017.

EMBASE ENTERED 10:12:09 ON 08 FEB 2017		RESULTS
**=> s elbow disease+nt/ct**		
L1	ELBOW DISEASE+NT/CT (5 TERMS)	5945
**=> s elbow injury/ct**		
L2	ELBOW INJURY/CT	1704
**=> s L1 or L2**		
L3	L1 OR L2	7302
**=> s occupation?/ct**		
L4	OCCUPATION?/CT	271962
**=> s work?/ct**		
L5	WORK?/CT	247856
**=> s L4 or L5**		
L6	L4 OR L5	477782
**=> s L3 and L6**		
L7	L3 AND L6	443
**=> s risk factor?**		
	RISK	2845952
	FACTOR?	4438575
L8	RISK FACTOR?	1005760
	(RISK(W)FACTOR?)	
**=> s L3 and L8**		
L9	L3 AND L8	323
**=> s L7 or L9**		
L10	L7 OR L9	667
**=> s 20070901-20170207/pd**		
L11	20070901-20170207/PD	11441788
	(20070901-20170207/PD)	
**=> s L10 and L11**		
L12	L10 AND L11	321
**=> s L12 not case report/ct**		
	CASE REPORT/CT	2171856
L13	L12 NOT CASE REPORT/CT	295
**=> s L13 not child?/ti,ct**		
	CHILD?/TI	796065
	CHILD?/CT	1842185
L14	L13 NOT CHILD?/TI,CT	264
**=> s L14 and en/la**		
	EN/LA	26116521
L15	L14 AND EN/LA	249
**=> s L15 and ab/fa**		
	AB/FA	21970944
L16	L15 AND AB/FA	226

File V – Cochrane Work search strategy – 06th January 2017

Cochrane Work (available from: http://work.cochrane.org/cochrane-reviews-about-occupational-safety-and-health, accessed 06th January 2017) were scanned manually via title and abstract by D.H.S., because computer-based search including e. g. MeSH terms, other key words, or search strings were not supported by Cochrane Work in January 2017.

File VI – Table S4: Documentation of all included and excluded studies

Table S4. Documentation of all included and excluded studies.

Identification			Scanning title & abstract						Scanning full-text				After scanning title & abstract, full-text, discussions about eligibility				
No.	Reference	Source	Rev-iew-er D.H.S no = 0; yes = 1	Re-vie-wer B.S. no = 0; yes = 1	Inclusion/ Exclusion after scanning abstracts, both no = 0; DHS yes & BS no = 1; DHS no & BS yes = 2; both yes = 3			Exclusion; after scanning title & abstract (incl./excl. = 1) = 0 discussion necessary (incl./excl. = 2) = 1 discussion necessary (incl./excl. = 3) = 1 inclusion for scanning full-text (incl./excl. = 3) = 2	After first discussion exclusion = 0 discussion necessary = 1 inclusion = 2	After Scanning full-text exclusion = 0 inclusion = 1	After Scanning full-text and after first discussion exclusion; because abstract is missing = 0 exclusion; after scanning title & abstract (no; no) = 1 exclusion; abstract (yes, no), full-text (no) = 2 discussion necessary; DHS exclusion after scanning full-text, BS inclusion after scanning title & abstract = 3 discussion necessary = 4 inclusion for scanning full-text = 5		Exclusion / inclusion after initial full-text scanning no = 0; yes = 1		Exclusion / inclusion after checking for eligibility and last discussions final rating no = 0; yes = 1		Reasons for exclus-ion of single studies a) to e) (see chapter 'Inclu-sion criteria' in 'Materi-als and Met-hods)
1	Aasmoe, L., et al. (2008). "Musculoskeletal symptoms among seafood production workers in North Norway." Occup Med (Lond) 58(1): 64-70.	EMBASE_08_02_2017	no	0	yes	1	2	1	1	1	discussion necessary	4	no	0	no	0	e
2	Abledu, J. K., Offei, E. B., & Abledu, G. K. (2014). Predictors of Work-Related Musculoskeletal Disorders among Commercial Minibus Drivers in Accra Metropolis, Ghana. Advances in Epidemiology, 2014.	added by Seidel	yes	1	no	0	1	1	0	0	exclusion; abstract (yes, no), full-text (no)	2	no	0	no	0	c, e
3	Abgarov, Alisa, et al. "Understanding trends and risk factors of swimming-related injuries in varsity swimmers." Clinical Kinesiology: Journal of the American Kinesiotherapy Association, vol. 66, no. 2, 2012, p. 24+. Academic OneFile, Accessed 21 Feb. 2017.	EMBASE_08_02_2017	no	0	no	0	0	0	0	0	exclusion; after scanning title & abstract (no; no)	1	no	0	no	0	b
4	Abrams, G. D., et al. (2012). "Risk factors for development of heterotopic ossification of the elbow after fracture fixation." J Shoulder Elbow Surg 21(11): 1550-1554.	EMBASE_08_02_2017	no	0	no	0	0	0	0	0	exclusion; after scanning title & abstract (no; no)	1	no	0	no	0	b

5	Aghilinejad, M., et al. (2012). "Work-related musculoskeletal complaints among workers of Iranian aluminum industries." Arch Environ Occup Health 67(2): 98-102.	EMBASE_08_02_2017	yes	1	yes	1	3	2	2	1	inclusion for scanning full-text	5	no	0	no	0	e
6	Ahmed, S. (2013). Risk factors of tennis elbow patients attended at two selected organizations in Dhaka (Doctoral dissertation, Department of Physiotherapy, Bangladesh Health Professions Institute, CRP).	added by Seidel	yes	1	no	0	1	1	1	1	discussion necessary	4	yes	1	no	0	b
7	Aitchison, L. P., et al. (2016). "The ergonomics of laparoscopic surgery: a quantitative study of the time and motion of laparoscopic surgeons in live surgical environments." Surg Endosc 30(11): 5068-5076.	added by Seidel	yes	1	yes	1	3	2	1	1	discussion necessary; DHS exclusion after scanning full-text, BS inclusion after scanning title & abstract	3	no	0	no	0	d
8	Alnaser, M. Z. (2007). Occupational musculoskeletal injuries in the health care environment and its impact on occupational therapy practitioners: a systematic review. Work, 29(2), 89-100.	added by Seidel	no	0	no	0	0	0	0	0	exclusion; after scanning title & abstract (no; no)	1	no	0	no	0	e
9	Alperovitch-Najenson, D., et al. (2010). "Upper body quadrant pain in bus drivers." Arch Environ Occup Health 65(4): 218-223.	EMBASE_08_02_2017	no	0	no	0	0	0	0	0	exclusion; after scanning title & abstract (no; no)	1	no	0	no	0	c, e
10	Alqahtani, S. M., et al. (2016). "Adult Reconstructive Surgery: A High-Risk Profession for Work-Related Injuries." J Arthroplasty 31(6): 1194-1198.	EMBASE_08_02_2017	no	0	no	0	0	0	0	0	exclusion; after scanning title & abstract (no; no)	1	no	0	no	0	c, e
11	Alrowayeh, H. N., et al. (2010). "Prevalence, characteristics, and impacts of work-related musculoskeletal disorders: a survey among physical therapists in the State of Kuwait." BMC Musculoskelet Disord 11: 116.	EMBASE_08_02_2017	no	0	no	0	0	0	0	0	exclusion; after scanning title & abstract (no; no)	1	no	0	no	0	c, e
12	Altinisik, J., et al. (2015). "The BstUI and DpnII Variants of the COL5A1 Gene Are Associated With Tennis Elbow." Am J Sports Med 43(7): 1784-1789.	PubMed_03_02_2017/ EMBASE_08_02_2017	no	0	no	0	0	0	0	0	exclusion; after scanning title & abstract (no; no)	1	no	0	no	0	b, c, e
13	Andersen, J. H., Haahr, J. P., & Frost, P. (2007). Risk factors for more severe regional musculoskeletal symptoms: A two-year prospective study of a general working population. Arthritis & Rheumatology, 56(4), 1355-1364.	added by Seidel	yes	1	yes	1	3	2	2	1	inclusion for scanning full-text	5	yes	1	no	0	d
14	Andersen, J. H., et al. (2011). "Risk factors for neck and upper extremity disorders among computers users and the effect of interventions: an overview of systematic reviews." PLoS One 6(5): e19691.	added by Seidel	no	0	no	0	0	0	0	0	exclusion; after scanning title & abstract (no; no)	1	no	0	no	0	c, e
15	Andersen, J. H., et al. (2012). "Computer use and ulnar neuropathy: results from a case-referent study." Work 41 Suppl 1: 2434-2437.	PubMed_03_02_2017	yes	1	yes	1	3	2	2	1	inclusion for scanning full-text	5	yes	1	no	0	results in abstract, but not in full-text, exclusion after discussion of content

16	Andersen, L. L., et al. (2010). "Effect of physical exercise interventions on musculoskeletal pain in all body regions among office workers: a one-year randomized controlled trial." Man Ther 15(1): 100-104.	EMBASE_08_02_2017_Abstract_not found	no	0	no	0	0	0	0	0	exclusion; because abstract is missing	0	no	0	no	0	a
17	Anderson, A. M., Meador, K. A., McClure, L. R., Makrozahopoulos, D., Brooks, D. J., & Mirka, G. A. (2007). A biomechanical analysis of anterior load carriage. Ergonomics, 50(12), 2104-2117.	added by Seidel	yes	1	no	0	1	1	1	1	discussion necessary	4	no	0	no	0	d
18	Anderson, M. W. and B. A. Alford (2010). "Overhead throwing injuries of the shoulder and elbow." Radiol Clin North Am 48(6): 1137-1154.	EMBASE_08_02_2017	no	0	no	0	0	0	0	0	exclusion; after scanning title & abstract (no; no)	1	no	0	no	0	b
19	Lindegård Andersson, A. (2007). Working technique during computer work Associations with biomechanical and psychological strain, neck and upper extremity musculoskeletal symptoms. Inst of Medicine. Dept of Public Health and Community Medicine.	added by Seidel	yes	1	yes	1	3	2	2	1	inclusion for scanning full-text	5	no	0	no	0	e
20	Andrade, D. M. and A. Barbosa-Branco (2015). "Synovitis and tenosynovitis in Brazil: analysis of sickness benefit claims." Rev Bras Epidemiol 18(1): 194-207.	PubMed_03_02_2017	no	0	no	0	0	0	0	0	exclusion; after scanning title & abstract (no; no)	1	no	0	no	0	c
21	Aragon, V. J., et al. (2012). "Trunk-rotation flexibility in collegiate softball players with or without a history of shoulder or elbow injury." J Athl Train 47(5): 507-513.	added by Seidel	no	0	no	0	0	0	0	0	exclusion; after scanning title & abstract (no; no)	1	no	0	no	0	b, c
22	Arcury, T. A., et al. (2016). "The effects of work organization on the health of immigrant manual workers: A longitudinal analysis." Arch Environ Occup Health 71(2): 66-73.	PubMed_03_02_2017	no	0	no	0	0	0	0	0	exclusion; after scanning title & abstract (no; no)	1	no	0	no	0	c
23	Aroui, H., et al. (2016). "Musculoskeletal disorders of the hand and wrist and population attributable fraction of risk of exposure to biomechanical constraints." Ann Phys Rehabil Med 59s: e113-e114.	added by Seidel	no	0	no	0	0	0	0	0	exclusion; after scanning title & abstract (no; no)	1	no	0	no	0	d
24	Artati, S. P., Van der Smagt, P., Krüger, D. I. N., & Baena, J. M. B. Calculation of Human Arm Stiffness using a Biomechanical Model.	added by Seidel	no	0	no	0	0	0	0	0	exclusion; after scanning title & abstract (no; no)	1	no	0	no	0	b, d, e
25	Arvidsson, I., et al. (2008). "Follow-up study of musculoskeletal disorders 20 months after the introduction of a mouse-based computer system." Scand J Work Environ Health 34(5): 374-380.	EMBASE_08_02_2017	yes	1	yes	1	3	2	2	1	inclusion for scanning full-text	5	no	0	no	0	e
26	Auerbach, J. D., et al. (2011). "Musculoskeletal disorders among spine surgeons: results of a survey of the Scoliosis Research Society membership." Spine (Phila Pa 1976) 36(26): E1715-1721.	PubMed_03_02_2017	no	0	no	0	0	0	0	0	exclusion; after scanning title & abstract (no; no)	1	no	0	no	0	c
27	Bagher, O. M., et al. (2011). "Pattern of rheumatic diseases in two outpatient clinics in Iran: similarities with some different features." Indian J Med Sci 65(1): 7-17.	EMBASE_08_02_2017	no	0	no	0	0	0	0	0	exclusion; after scanning title & abstract (no; no)	1	no	0	no	0	c, d
28	Baker SR, Patel RH, Lelkes V, Castro A 3rd, Sarmast U, Whang J. Non-spinal musculoskeletal malpractice suits against radiologists in the USA--rates, anatomic locations, and payments in a survey of 8,265 radiologists. Emerg Radiol. 2014 Feb;21(1):29-34. doi: 10.1007/s10140-013-1154-4. PubMed PMID: 23996223.	EMBASE_08_02_2017	no	0	no	0	0	0	0	0	exclusion; after scanning title & abstract (no; no)	1	no	0	no	0	c
29	Balogh I, Ohlsson K, Nordander C, Björk J, Hansson GÅ. The importance of work organization on workload and musculoskeletal health--Grocery store work as a model. Appl Ergon. 2016 Mar;53 Pt A:143-51. doi: 10.1016/j.apergo.2015.09.004. PubMed PMID: 26464034.	EMBASE_08_02_2017	yes	1	no	0	1	1	1	1	discussion necessary	4	no	0	no	0	e

30	Bandinelli, F., et al. (2015). "Ultrasonographic wrist and hand abnormalities in early psoriatic arthritis patients: correlation with clinical, dermatological, serological and genetic indices." Clin Exp Rheumatol 33(3): 330-335.	PubMed_03_02_2017	no	0	no	0	0	0	0	0	exclusion; after scanning title & abstract (no; no)	1	no	0	no	0	b, c
31	Bao, S., et al. (2011). "Comparison of two different methods for performing combination analysis of force and posture risk factors in an epidemiological study." Scand J Work Environ Health 37(6): 512-524.	PubMed_03_02_2017	no	0	no	0	0	0	0	0	exclusion; after scanning title & abstract (no; no)	1	no	0	no	0	c, e
32	Bao, S. S., et al. (2016). "Impact of Work Organizational Factors on Carpal Tunnel Syndrome and Epicondylitis." J Occup Environ Med 58(8): 760-764.	EMBASE_08_02_2017	yes	1	no	0	1	1	0	0	exclusion; abstract (yes, no), full-text (no)	2	no	0	no	0	d
33	Barrero, L. H., et al. (2012). "Physical workloads of the upper-extremity among workers of the Colombian flower industry." Am J Ind Med 55(10): 926-939.	PubMed_03_02_2017	yes	1	yes	1	3	2	2	1	inclusion for scanning full-text	5	no	0	no	0	e
34	Bepko, J. and K. Mansalis (2016). "Common Occupational Disorders: Asthma, COPD, Dermatitis, and Musculoskeletal Disorders." Am Fam Physician 93(12): 1000-1006.	EMBASE_08_02_2017	yes	1	no	0	1	1	1	1	discussion necessary	4	no	0	no	0	c, e
35	Bethapudi, S., et al. (2013). "Elbow injuries at the London 2012 Summer Olympic Games: demographics and pictorial imaging review." AJR Am J Roentgenol 201(3): 535-549.	PubMed_03_02_2017	no	0	no	0	0	0	0	0	exclusion; after scanning title & abstract (no; no)	1	no	0	no	0	b, c, d, e
36	Bheeshma, B., Manoj Deepak, M., Prabhu Thangaraju, Venkatachalam, K. 2015. Prospective Study of the Evaluation of Autologous Blood Transfusion in the Treatment of Lateral Epicondylitis. Research Journal of Pharmaceutical, Biological and Chemical Sciences. 6(2) Page No. 970-974	EMBASE_08_02_2017	no	0	no	0	0	0	0	0	exclusion; after scanning title & abstract (no; no)	1	no	0	no	0	b, c, e
37	Bigorre, N., et al. (2011). "Lateral epicondylitis treatment by extensor carpi radialis fasciotomy and radial nerve decompression: is outcome influenced by the occupational disease compensation aspect?" Orthop Traumatol Surg Res 97(2): 159-163.	EMBASE_08_02_2017	no	0	no	0	0	0	0	0	exclusion; after scanning title & abstract (no; no)	1	no	0	no	0	c, e
38	Bonfiglioli, R., et al. (2015). "Occupational mononeuropathies in industry." Handb Clin Neurol 131: 411-426.	added by Seidel	yes	1	yes	1	3	2	1	1	discussion necessary	4	no	0	no	0	e
39	Bongartz, T., et al. (2015). "Dual-energy CT for the diagnosis of gout: an accuracy and diagnostic yield study." Ann Rheum Dis 74(6): 1072-1077.	PubMed_03_02_2017	no	0	no	0	0	0	0	0	exclusion; after scanning title & abstract (no; no)	1	no	0	no	0	b, c, d, e
40	Bonneterre, V., et al. (2010). "Programmed health surveillance and detection of emerging diseases in occupational health: contribution of the French national occupational disease surveillance and prevention network (RNV3P)." Occup Environ Med 67(3): 178-186.	EMBASE_08_02_2017	yes	1	no	0	1	1	1	1	discussion necessary	4	no	0	no	0	c, e
41	Boocock, M. G., et al. (2009). "A framework for the classification and diagnosis of work-related upper extremity conditions: systematic review." Semin Arthritis Rheum 38(4): 296-311.	EMBASE_08_02_2017_Abstract_not found	no	0	no	0	0	0	0	0	exclusion; because abstract is missing	0	no	0	no	0	a
42	Bosch, T., Mathiassen, S. E., Visser, B., Looze, M. D., & Dieën, J. V. (2011). The effect of work pace on workload, motor variability and fatigue during simulated light assembly work. Ergonomics, 54(2), 154-168.	added by Seidel	yes	1	yes	1	3	2	1	1	discussion necessary; DHS exclusion after scanning full-text, BS inclusion after scanning title & abstract	3	no	0	no	0	d

43	Boschman, J. S., van der Molen, H. F., Sluiter, J. K., & Frings-Dresen, M. H. (2011). Occupational demands and health effects for bricklayers and construction supervisors: A systematic review. American journal of industrial medicine, 54(1), 55-77.	added by Seidel	yes	1	yes	1	3	2	2	1	inclusion for scanning full-text	5	yes	1	no	0	e
44	Boschman, J. S., et al. (2012). "Musculoskeletal disorders among construction workers: a one-year follow-up study." BMC Musculoskelet Disord 13: 196.	EMBASE_08_02_2017	yes	1	no	0	1	1	1	1	discussion necessary	4	no	0	no	0	c, e
45	Briskin, S. M. (2012). "Injuries and medical issues in softball." Curr Sports Med Rep 11(5): 265-271.	EMBASE_08_02_2017	no	0	no	0	0	0	0	0	exclusion; after scanning title & abstract (no; no)	1	no	0	no	0	b, c, d, e
46	Bruce JR, Hess R, Joyner P, Andrews JR. How much valgus instability can be expected with ulnar collateral ligament (UCL) injuries? A review of 273 baseball players with UCL injuries. J Shoulder Elbow Surg. 2014 Oct;23(10):1521-6. doi: 10.1016/j.jse.2014.05.015. PubMed PMID: 25220199.	EMBASE_08_02_2017	no	0	no	0	0	0	0	0	exclusion; after scanning title & abstract (no; no)	1	no	0	no	0	b, c, d, e
47	Buchanan, K. A., et al. (2016). "Proximal forearm extensor muscle strain is reduced when driving nails using a shock-controlled hammer." Clin Biomech (Bristol, Avon) 38: 22-28.	added by Seidel	no	0	no	0	0	0	0	0	exclusion; after scanning title & abstract (no; no)	1	no	0	no	0	c, e
48	Bugajska, J., et al. (2013). "Psychological factors at work and musculoskeletal disorders: a one year prospective study." Rheumatol Int 33(12): 2975-2983.	PubMed_03_02_2017/ EMBASE_08_02_2017	no	0	no	0	0	0	0	0	exclusion; after scanning title & abstract (no; no)	1	no	0	no	0	c, e
49	Bunata R, Icenogle K. Cerebral palsy of the elbow and forearm. J Hand Surg Am. 2014 Jul;39(7):1425-32. doi: 0.1016/j.jhsa.2013.12.017. Review. PubMed PMID: 24969499.	EMBASE_08_02_2017	no	0	no	0	0	0	0	0	exclusion; after scanning title & abstract (no; no)	1	no	0	no	0	b, c, e
50	Burnett, D. R., & Campbell-Kyureghyan, N. H. (2010). Quantification of scan-specific ergonomic risk-factors in medical sonography. International Journal of Industrial Ergonomics, 40(3), 306-314.	added by Seidel	yes	1	yes	1	3	2	1	1	discussion necessary; DHS exclusion after scanning full-text, BS inclusion after scanning title & abstract	3	no	0	no	0	d
51	Burton, A. K., Kendall, N. A., Pearce, B. G., Birrell, L. N., & Bainbridge, L. C. (2009). Management of work-relevant upper limb disorders: a review. Occupational medicine, 59(1), 44-52.	added by Seidel	no	0	no	0	0	0	0	0	exclusion; after scanning title & abstract (no; no)	1	no	0	no	0	c, e
52	Burton, A. K. (2008). Work-relevant upper limb disorders: their characterisation, causation and management. Occupational Health at Work, 5(4), 13-18.	added by Seidel	yes	1	yes	1	3	2	1	1	discussion necessary; DHS exclusion after scanning full-text, BS inclusion after scanning title & abstract	3	no	0	no	0	e
53	Cai J, Zhou Y, Chen S, Sun Y, Yuanming O, Ruan H, Fan C. Ulnar neuritis after open elbow arthrolysis combined with ulnar nerve subcutaneous transposition for post-traumatic elbow stiffness: outcome and risk factors. J Shoulder Elbow Surg. 2016 Jun;25(6):1027-33. doi: 10.1016/j.jse.2016.01.013. PubMed PMID: 27039670.	EMBASE_08_02_2017	no	0	no	0	0	0	0	0	exclusion; after scanning title & abstract (no; no)	1	no	0	no	0	b, c, e
54	Cabral, P., et al. (2013). "Correlation of morphologic and pathologic features of the various tendon groups around the ankle: MR imaging investigation." Skeletal Radiol 42(10): 1393-1402.	PubMed_03_02_2017	no	0	no	0	0	0	0	0	exclusion; after scanning title & abstract (no; no)	1	no	0	no	0	d

55	Cai J, Wang W, Yan H, Sun Y, Chen W, Chen S, Fan C. Complications of Open Elbow Arthrolysis in Post-Traumatic Elbow Stiffness: A Systematic Review. PLoS One. 2015 Sep 18;10(9):e0138547. doi: 10.1371/journal.pone.0138547. Review. PubMed PMID: 26383106; PubMed Central PMCID: PMC4575202.	EMBASE_08_02_2017	no	0	no	0	0	0	0	0	exclusion; after scanning title & abstract (no; no)	1	no	0	no	0	b, c, e
56	Cantley, L. F., Taiwo, O. A., Galusha, D., Barbour, R., Slade, M. D., Tessier-Sherman, B., & Cullen, M. R. (2014). Effect of systematic ergonomic hazard identification and control implementation on musculoskeletal disorder and injury risk. Scandinavian journal of work, environment & health, 40(1), 57.	added by Seidel	yes	1	no	0	1	1	0	0	exclusion; abstract (yes, no), full-text (no)	2	no	0	no	0	c, e
57	Carter GT, Weiss MD. Diagnosis and Treatment of Work-Related Proximal Median and Radial Nerve Entrapment. Phys Med Rehabil Clin N Am. 2015 Aug;26(3):539-49. doi: 10.1016/j.pmr.2015.04.001. Review. PubMed PMID: 26231964.	EMBASE_08_02_2017	no	0	no	0	0	0	0	0	exclusion; after scanning title & abstract (no; no)	1	no	0	no	0	c, e
58	Cartwright, M. S., et al. (2016). "Examining the association between musculoskeletal injuries and carpal tunnel syndrome in manual laborers." Muscle Nerve 54(1): 31-35.	added by Seidel	no	0	no	0	0	0	0	0	exclusion; after scanning title & abstract (no; no)	1	no	0	no	0	c, e
59	Chahal, J., et al. (2010). "Generalized ligamentous laxity as a predisposing factor for primary traumatic anterior shoulder dislocation." J Shoulder Elbow Surg 19(8): 1238-1242.	PubMed_03_02_2017	no	0	no	0	0	0	0	0	exclusion; after scanning title & abstract (no; no)	1	no	0	no	0	b, d
60	Chalmers PN, Sgroi T, Riff AJ, Lesniak M, Sayegh ET, Verma NN, Cole BJ, Romeo AA. Correlates With History of Injury in Youth and Adolescent Pitchers. Arthroscopy. 2015 Jul;31(7):1349-57. doi: 10.1016/j.arthro.2015.03.017. PubMed PMID: 25953122.	EMBASE_08_02_2017_Double	no	0	no	0	0	0	0	0	exclusion; after scanning title & abstract (no; no)	1	no	0	no	0	b
61	Chang, C. W., et al. (2008). "Increased carrying angle is a risk factor for nontraumatic ulnar neuropathy at the elbow." Clin Orthop Relat Res 466(9): 2190-2195.	PubMed_03_02_2017	yes	1	yes	1	3	2	2	1	inclusion for scanning full-text	5	yes	1	no	0	exclusion after discussion of content, diagnostic study
62	Chang, J. H., et al. (2012). "Prevalence of musculoskeletal disorders and ergonomic assessments of cleaners." Am J Ind Med 55(7): 593-604.	PubMed_03_02_2017	yes	1	yes	1	3	2	1	1	discussion necessary; DHS exclusion after scanning full-text, BS inclusion after scanning title & abstract	3	no	0	no	0	e
63	Chassé M, Fergusson DA, Chen Y. Body mass index and the risk of injury in adults: a cross-sectional study. Int J Obes (Lond). 2014 Nov;38(11):1403-9. doi: 10.1038/ijo.2014.28. PubMed PMID: 24525959.	EMBASE_08_02_2017	no	0	no	0	0	0	0	0	exclusion; after scanning title & abstract (no; no)	1	no	0	no	0	c, d, e
64	Chau, Leo, and Richard Wells. "Biomechanical loading on the hand, wrist, and forearm when holding a tablet computer." IIE Transactions on Occupational Ergonomics and Human Factors 3.2 (2015): 105-114.	added by Seidel	yes	1	yes	1	3	2	2	1	inclusion for scanning full-text	5	no	0	no	0	d
65	Cherniack M, Brammer AJ, Lundstrom R, et al. The effect of different warming methods on sensory nerve conduction velocity in shipyard workers occupationally exposed to hand-arm vibration. International archives of occupational and environmental health 2008;81(8):1045-58. doi: 10.1007/s00420-007-0299-4 [published Online First: 2008/01/16]	PubMed_03_02_2017	no	0	no	0	0	0	0	0	exclusion; after scanning title & abstract (no; no)	1	no	0	no	0	d

66	Castillo-Lozano R., Casuso-Holgado M.J. , A comparison musculoskeletal injuries among junior and senior paddle-tennis players, Science & Sports, Volume 30, Issue 5, October 2015, Pages 268-274, ISSN 0765-1597, http://dx.doi.org/10.1016/j.scispo.2015.03.005. (http://www.sciencedirect.com/science/article/pii/S0765159715000684)	EMBASE_08_02_2017	no	0	no	0	0	0	0	0	exclusion; after scanning title & abstract (no; no)	1	no	0	no	0	b
67	Chelsea Hopkins, Sai-Chuen Fu, Eldrich Chua, Xiaorui Hu, Christer Rolf, Ville M. Mattila, Ling Qin, Patrick Shu-Hang Yung, Kai-Ming Chan, Critical review on the socio-economic impact of tendinopathy, Asia-Pacific Journal of Sports Medicine, Arthroscopy, Rehabilitation and Technology, Volume 4, April 2016, Pages 9-20, ISSN 2214-6873, http://dx.doi.org/10.1016/j.asmart.2016.01.002.	EMBASE_08_02_2017	no	0	no	0	0	0	0	0	exclusion; after scanning title & abstract (no; no)	1	no	0	no	0	c, e
68	Chimenti RL, Van Dillen LR, Prather H, Hunt D, Chimenti PC, Khoo-Summers L. Underutilization of worker's compensation insurance among professional orchestral musicians. Med Probl Perform Art. 2013 Mar;28(1):54-60. PubMed PMID: 23462905.	EMBASE_08_02_2017	yes	1	no	0	1	1	0	0	exclusion; abstract (yes, no), full-text (no)	2	no	0	no	0	c, e
69	Cho, C. Y., et al. (2012). "Musculoskeletal symptoms and associated risk factors among office workers with high workload computer use." J Manipulative Physiol Ther 35(7): 534-540.	EMBASE_08_02_2017	no	0	no	0	0	0	0	0	exclusion; after scanning title & abstract (no; no)	1	no	0	no	0	d
70	Choi, S. D., Yuan, L., & Borchardt, J. G. (2014, September). Critical analyses of work-related musculoskeletal disorders and practical solutions in construction. In Proceedings of the Human Factors and Ergonomics Society Annual Meeting (Vol. 58, No. 1, pp. 1633-1637). SAGE Publications.	added by Seidel	yes	1	no	0	1	1	0	0	exclusion; abstract (yes, no), full-text (no)	2	no	0	no	0	c, e
71	Choung, S. D., et al. (2016). "Comparison of muscle activity of wrist extensors and kinematics of wrist joint during wrist extension in automobile assembly line workers with and without lateral epicondylitis." Work 55(1): 241-247.	added by Seidel	yes	1	yes	1	3	2	2	1	inclusion for scanning full-text	5	no	0	no	0	e
72	Chu, P. C., et al. (2013). "The impact of occupational health service network and reporting system in Taiwan." Int J Occup Environ Health 19(4): 352-362.	PubMed_03_02_2017	no	0	no	0	0	0	0	0	exclusion; after scanning title & abstract (no; no)	1	no	0	no	0	c, e
73	Chung KC, Lark ME. Upper Extremity Injuries in Tennis Players: Diagnosis, Treatment, and Management. Hand Clin. 2017 Feb;33(1):175-186. doi: 10.1016/j.hcl.2016.08.009. Review. PubMed PMID: 27886833; PubMed Central PMCID: PMC5125509.	EMBASE_08_02_2017	no	0	no	0	0	0	0	0	exclusion; after scanning title & abstract (no; no)	1	no	0	no	0	a
74	Chung, W. M., et al. (2012). "Musculoskeletal injuries in elite able-bodied and wheelchair foil fencers--a pilot study." Clin J Sport Med 22(3): 278-280.	EMBASE_08_02_2017	no	0	no	0	0	0	0	0	exclusion; after scanning title & abstract (no; no)	1	no	0	no	0	a
75	Chung, Y. C., et al. (2013). "Risk of musculoskeletal disorder among Taiwanese nurses cohort: a nationwide population-based study." BMC Musculoskelet Disord 14: 144.	PubMed_03_02_2017	yes	1	yes	1	3	2	1	1	discussion necessary; DHS exclusion after scanning full-text, BS inclusion after scanning title & abstract	3	no	0	no	0	e
76	Claessen FM, Braun Y, van Leeuwen WF, Dyer GS, van den Bekerom MP, Ring D. What Factors are Associated With a Surgical Site Infection After Operative Treatment of an Elbow Fracture? Clin Orthop Relat Res. 2016 Feb;474(2):562-70. doi: 10.1007/s11999-015-4523-3. PubMed PMID: 26304043; PubMed Central PMCID: PMC4709303.	EMBASE_08_02_2017	no	0	no	0	0	0	0	0	exclusion; after scanning title & abstract (no; no)	1	no	0	no	0	b, c, e

77	Clavet, H., et al. (2008). "Joint contracture following prolonged stay in the intensive care unit." Cmaj 178(6): 691-697.	PubMed_03_02_2017	no	0	no	0	0	0	0	0	exclusion; after scanning title & abstract (no; no)	1	no	0	no	0	b
78	Clement, N. D. and D. E. Porter (2013). "Forearm deformity in patients with hereditary multiple exostoses: factors associated with range of motion and radial head dislocation." J Bone Joint Surg Am 95(17): 1586-1592.	PubMed_03_02_2017	no	0	no	0	0	0	0	0	exclusion; after scanning title & abstract (no; no)	1	no	0	no	0	b
79	Cohn CS, Lockhart E, McCullough JJ. The use of autologous platelet-rich plasma in the orthopedic setting. Transfusion. 2015 Jul;55(7):1812-20. doi: 10.1111/trf.13005. PubMed PMID: 25646697.	EMBASE_08_02_2017	no	0	no	0	0	0	0	0	exclusion; after scanning title & abstract (no; no)	1	no	0	no	0	c, e
80	Colantoni, A., Marucci, A., Monarca, D., Pagniello, B., Cecchini, M., & Bedini, R. (2012). The risk of musculoskeletal disorders due to repetitive movements of upper limbs for workers employed to vegetable grafting. Journal of Food, Agriculture & Environment, 10(3&4), 14-18.	EMBASE_08_02_2017	yes	1	yes	1	3	2	2	1	inclusion for scanning full-text	5	yes	1	no	0	d
81	Collins, R. M., Janse Van Rensburg, D. C. & Patricios, J. S. Common work-related musculoskeletal strains and injuries. S Afr Fam Pract 2011;53(3):240-246	EMBASE_08_02_2017	no	0	yes	1	2	1	1	1	discussion necessary	4	no	0	no	0	d
82	Conte SA, Hodgins JL, ElAttrache NS, Patterson-Flynn N, Ahmad CS. Media perceptions of Tommy John surgery. Phys Sportsmed. 2015 Nov;43(4):375-80. doi: 10.1080/00913847.2015.1077098. PubMed PMID: 26307904.	EMBASE_08_02_2017	no	0	no	0	0	0	0	0	exclusion; after scanning title & abstract (no; no)	1	no	0	no	0	b, c, e
83	Coombes, B. K., et al. (2010). "Efficacy and safety of corticosteroid injections and other injections for management of tendinopathy: a systematic review of randomised controlled trials." Lancet 376(9754): 1751-1767.	EMBASE_08_02_2017	no	0	no	0	0	0	0	0	exclusion; after scanning title & abstract (no; no)	1	no	0	no	0	a, c, e
84	Crawford, J. O., Laiou, E., Spurgeon, A., & McMillan, G. (2008). Musculoskeletal disorders within the telecommunications sector—a systematic review. International Journal of Industrial Ergonomics, 38(1), 56-72.	added by Seidel	no	0	no	0	0	0	0	0	exclusion; after scanning title & abstract (no; no)	1	no	0	no	0	c, e
85	Creuzé, A. 2014). Interest of botulinum toxin in tendinopathies: Review of literature. Annals of Physical and Rehabilitation Medicine Volume 57, n° S1 page e269 (mai 2014) Doi : 10.1016/j.rehab.2014.03.975	EMBASE_08_02_2017	no	0	no	0	0	0	0	0	exclusion; after scanning title & abstract (no; no)	1	no	0	no	0	b, c, e
86	Cudlip, A. C., et al. (2015). "Effects of sitting and standing on upper extremity physical exposures in materials handling tasks." Ergonomics 58(10): 1637-1646.	PubMed_03_02_2017	yes	1	no	0	1	1	0	0	exclusion; abstract (yes, no), full-text (no)	2	no	0	no	0	c, e
87	Cunha-Miranda, L., et al. (2010). "Prevalence of rheumatic occupational diseases - PROUD study." Acta Reumatol Port 35(2): 215-226.	PubMed_03_02_2017	yes	1	no	0	1	1	0	0	exclusion; abstract (yes, no), full-text (no)	2	no	0	no	0	c, e
88	Curti S, Sauni R, Spreeuwers D, De Schryver A, Valenty M, Rivière S, Mattioli S. Interventions to increase the reporting of occupational diseases by physicians. Cochrane Database of Systematic Reviews 2015, Issue 3. Art. No.: CD010305. DOI: 10.1002/14651858.CD010305.pub2.	CochraneWork_06_01_2017	no	0	no	0	0	0	0	0	exclusion; after scanning title & abstract (no; no)	1	no	0	no	0	c, e
89	Curwin, S., et al. (2013). "The Healthy LifeWorks project: The effect of a comprehensive workplace wellness program on the prevalence and severity of musculoskeletal disorders in a Canadian government department." J Occup Environ Med 55(6): 628-633.	EMBASE_08_02_2017	no	0	no	0	0	0	0	0	exclusion; after scanning title & abstract (no; no)	1	no	0	no	0	c, e

90	Dabholkar, Ajit, Sinan Tejani, and Sujata Yardi. "Common Musculoskeletal Injuries in Rock Climbers." Indian Journal of Public Health Research & Development 5.4 (2014): 184-189.	EMBASE_08_02_2017	no	0	no	0	0	0	0	0	exclusion; after scanning title & abstract (no; no)	1	no	0	no	0	b
91	da Costa, B. R. and E. R. Vieira (2010). "Risk factors for work-related musculoskeletal disorders: A systematic review of recent longitudinal studies." Am J Ind Med 53(3): 285-323.	added by Seidel	yes	1	yes	1	3	2	2	1	inclusion for scanning full-text	5	yes	1	no	0	systematic review, no single study
92	da Costa, J. T., et al. (2015). "Incidence and prevalence of upper-limb work related musculoskeletal disorders: A systematic review." Work 51(4): 635-644.	added by Seidel	yes	1	no	0	1	1	1	1	discussion necessary	4	no	0	no	0	c, e
93	Dahlquist M, Leisz MC, Finkelstein M. The club-level road cyclist: injury, pain, and performance. Clin J Sport Med. 2015 Mar;25(2):88-94. doi: 10.1097/JSM.0000000000000111. PubMed PMID: 24915174.	EMBASE_08_02_2017	no	0	no	0	0	0	0	0	exclusion; after scanning title & abstract (no; no)	1	no	0	no	0	b
94	Daruwalla JH, Daly CA, Seiler JG 3rd. Medial Elbow Injuries in the Throwing Athlete. Hand Clin. 2017 Feb;33(1):47-62. doi: 10.1016/j.hcl.2016.08.013. Review. PubMed PMID: 27886839.	EMBASE_08_02_2017	no	0	no	0	0	0	0	0	exclusion; after scanning title & abstract (no; no)	1	no	0	no	0	b
95	Darwish, M. A. and S. Z. Al-Zuhair (2013). "Musculoskeletal Pain Disorders among Secondary School Saudi Female Teachers." Pain Res Treat 2013: 878570.	added by Seidel	yes	1	no	0	1	1	0	0	exclusion; abstract (yes, no), full-text (no)	2	no	0	no	0	c, e
96	Das, B., Ghosh, T., & Gangopadhyay, S. (2012). Assessment of ergonomic and occupational health-related problems among female prawn seed collectors of Sunderbans, West Bengal, India. International Journal of Occupational Safety and Ergonomics, 18(4), 531-540.	added by Seidel	yes	1	yes	1	3	2	1	1	discussion necessary; DHS exclusion after scanning full-text, BS inclusion after scanning title & abstract	3	no	0	no	0	e
97	Das De, S., et al. (2013). "Contribution of kinesophobia and catastrophic thinking to upper-extremity-specific disability." J Bone Joint Surg Am 95(1): 76-81.	PubMed_03_02_2017	no	0	no	0	0	0	0	0	exclusion; after scanning title & abstract (no; no)	1	no	0	no	0	c, e
98	Davis WT, Fletcher SA, Guillamondegui OD. Musculoskeletal occupational injury among surgeons: effects for patients, providers, and institutions. J Surg Res. 2014 Jun 15;189(2):207-212.e6. doi: 10.1016/j.jss.2014.03.013. PubMed PMID: 24721601.	EMBASE_08_02_2017	no	0	no	0	0	0	0	0	exclusion; after scanning title & abstract (no; no)	1	no	0	no	0	c, e
99	Day, C. S., et al. (2010). "Effects of workers' compensation on the diagnosis and surgical treatment of patients with hand and wrist disorders." J Bone Joint Surg Am 92(13): 2294-2299.	EMBASE_08_02_2017	no	0	no	0	0	0	0	0	exclusion; after scanning title & abstract (no; no)	1	no	0	no	0	c, e
100	de Haan, J., Schep, N.W.L., Eygendaal, D., Kleinrensink, G-J., Tuinebreijer, W.E., den Hartog, D. (2011).Stability of the Elbow Joint: Relevant Anatomy and Clinical Implications of In Vitro Biomechanical Studies. The Open Orthopaedics Journal, 2011, Volume 5	added by Seidel	no	0	no	0	0	0	0	0	exclusion; after scanning title & abstract (no; no)	1	no	0	no	0	c, e
101	Delleman, N. J., & Dul, J. (2007). International standards on working postures and movements ISO 11226 and EN 1005-4. Ergonomics, 50(11), 1809-1819.	added by Seidel	yes	1	no	0	1	1	1	1	discussion necessary	4	no	0	no	0	c, e
102	Dempsey, P. G., Mathiassen, S. E., Jackson, J. A., & O'Brien, N. V. (2010). Influence of three principles of pacing on the temporal organisation of work during cyclic assembly and disassembly tasks. Ergonomics, 53(11), 1347-1358.	added by Seidel	yes	1	no	0	1	1	0	0	exclusion; abstract (yes, no), full-text (no)	2	no	0	no	0	c, e

103	Denbeigh, K., et al. (2013). "Wrist postures and forces in tree planters during three tree unloading conditions." Ergonomics 56(10): 1599-1607.	PubMed_03_02_2017	yes	1	yes	1	3	2	1	1	discussion necessary; DHS exclusion after scanning full-text, BS inclusion after scanning title & abstract	3	no	0	no	0	d
104	Dennerlein, J. T., & Johnson, P. W. (2006). Different computer tasks affect the exposure of the upper extremity to biomechanical risk factors. Ergonomics, 49(1), 45-61.	added by Seidel	yes	1	yes	1	3	2	1	1	discussion necessary; DHS exclusion after scanning full-text, BS inclusion after scanning title & abstract	3	no	0	no	0	d
105	Dennerlein, J. T., Kingma, I., Visser, B., & van Dieën, J. H. (2007). The contribution of the wrist, elbow and shoulder joints to single-finger tapping. Journal of biomechanics, 40(13), 3013-3022.	added by Seidel	yes	1	no	0	1	1	0	0	exclusion; abstract (yes, no), full-text (no)	2	no	0	no	0	b, d, e
106	Degen RM, Cancienne JM, Camp CL, Altchek DW, Dines JS, Werner BC. Three or more preoperative injections is the most significant risk factor for revision surgery after operative treatment of lateral epicondylitis: an analysis of 3863 patients. J Shoulder Elbow Surg. 2017 Jan 13. pii: S1058-2746(16)30567-5. doi: 10.1016/j.jse.2016.10.022. [Epub ahead of print] PubMed PMID: 28094190.	EMBASE_08_02_2017	no	0	no	0	0	0	0	0	exclusion; after scanning title & abstract (no; no)	1	no	0	no	0	b, c, e
107	Descatha, A., Roquelaure, Y., Evanoff, B. et al. Int Arch Occup Environ Health (2007) 81: 1. doi:10.1007/s00420-007-0180-5	added by Seidel	yes	1	no	0	1	1	1	1	discussion necessary	4	no	0	no	0	c, d, e
108	Descatha, A., et al.Do Symptoms And Physical Examination Findings Predict Elbow Pain And Functional Outcomes In A Working Population? J Occup Environ Med. 2014 November; 56(11): e131–e132. doi:10.1097/JOM.0000000000000293	added by Seidel	yes	1	no	0	1	1	1	1	discussion necessary	4	no	0	no	0	c, e
109	Descatha, A., et al. (2016). "Lateral Epicondylitis and Physical Exposure at Work? A Review of Prospective Studies and Meta-Analysis." Arthritis Care Res (Hoboken) 68(11): 1681-1687.	EMBASE_08_02_2017	yes	1	yes	1	3	2	2	1	inclusion for scanning full-text	5	yes	1	no	0	meta-analysis, no single study
110	Descatha, Alexis; Despreaux, Thomas; Calfee, Ryan P.; Evanoff, Bradley; and Saint-Lary, Olivier,"Progressive elbow pain." BMJ.353,. i1391. (2016). http://digitalcommons.wustl.edu/open_access_pubs/4881	added by Seidel	no	0	no	0	0	0	0	0	exclusion; because abstract is missing	0	no	0	no	0	a
111	Descatha, Alexis; Dale, Ann Marie; Silverstein, Barbara A.; Roquelaure, Yves; and Rempel, David, "Lateral epicondylitis: new evidence for work relatedness".Joint Bone Spine, 82, 1, 5-7. 2015.	added by Seidel	no	0	no	0	0	0	0	0	exclusion; because abstract is missing	0	no	0	no	0	a
112	Descatha, A., et al. (2013). "Self-reported physical exposure association with medial and lateral epicondylitis incidence in a large longitudinal study." Occup Environ Med 70(9): 670-673.	PubMed_03_02_2017/ EMBASE_08_02_2017	yes	1	yes	1	3	2	2	1	inclusion for scanning full-text	5	yes	1	yes	1	no
113	De Smedt, T., de Jong, A., Van Leemput, W., Lieven, D., & Van Glabbeek, F. (2007). Lateral epicondylitis in tennis: update on aetiology, biomechanics and treatment. British journal of sports medicine, 41(11), 816-819.	added by Seidel	no	0	no	0	0	0	0	0	exclusion; after scanning title & abstract (no; no)	1	no	0	no	0	b

114	Diesselhorst, M. M., et al. (2013). "Survey of upper extremity injuries among martial arts participants." Hand Surg 18(2): 151-157.	PubMed_03_02_2017	no	0	no	0	0	0	0	0	exclusion; after scanning title & abstract (no; no)	1	no	0	no	0	b
115	Dick, F. D., et al. (2011). "Workplace management of upper limb disorders: a systematic review." Occup Med (Lond) 61(1): 19-25.	EMBASE_08_02_2017	yes	1	yes	1	3	2	1	1	discussion necessary; DHS exclusion after scanning full-text, BS inclusion after scanning title & abstract	3	no	0	no	0	e
116	Dick, R. B., Lowe, B., Ming-Lun, L., & Krieg, E. F. (2015). Further Trends in Work-Related Musculoskeletal Disorders-A Comparison of Risk factors for Symptoms Using Quality of Work Life Data From the 2002, 2006 and 2010 General Social Survey. Journal of Occupational and Environmental Medicine / American College of Occupational and Environmental Medicine, 57(8), 910–928. http://doi.org/10.1097/JOM.0000000000000501	added by Seidel	yes	1	yes	1	3	2	1	1	discussion necessary; DHS exclusion after scanning full-text, BS inclusion after scanning title & abstract	3	no	0	no	0	e
117	Di Domizio, J., & Keir, P. J. (2010). Forearm posture and grip effects during push and pull tasks. Ergonomics, 53(3), 336-343.	added by Seidel	yes	1	yes	1	3	2	2	1	inclusion for scanning full-text	5	no	0	no	0	d
118	DiFiori, J. P., et al. (2014). Overuse Injuries and Burnout in Youth Sports: A Position Statement from the American Medical Society for Sports Medicine. Clin J Sport Med 2014;24:3–20)	added by Seidel	no	0	no	0	0	0	0	0	exclusion; after scanning title & abstract (no; no)	1	no	0	no	0	b
119	Dion, S., et al. (2017). "Are Passive Physical Modalities Effective for the Management of Common Soft Tissue Injuries of the Elbow?: A Systematic Review by the Ontario Protocol for Traffic Injury Management (OPTIMa) Collaboration." Clin J Pain 33(1): 71-86.	added by Seidel	no	0	no	0	0	0	0	0	exclusion; after scanning title & abstract (no; no)	1	no	0	no	0	c, e
120	Dohn, P., et al. (2012). "Adult post-traumatic radioulnar synostosis." Orthop Traumatol Surg Res 98(6): 709-714.	PubMed_03_02_2017	no	0	no	0	0	0	0	0	exclusion; after scanning title & abstract (no; no)	1	no	0	no	0	b, c, e
121	Doke, P. P., et al. (2011). "A clinico-epidemiological study of Chikungunya outbreak in Maharashtra State, India." Indian J Public Health 55(4): 313-316.	PubMed_03_02_2017	no	0	no	0	0	0	0	0	exclusion; after scanning title & abstract (no; no)	1	no	0	no	0	b, c, d, e
122	Douglas, K., et al. (2012). "Incidence and risk factors of heterotopic ossification following major elbow trauma." Orthopedics 35(6): e815-822.	EMBASE_08_02_2017	no	0	no	0	0	0	0	0	exclusion; after scanning title & abstract (no; no)	1	no	0	no	0	b, c, e
123	Dozono, K., et al. (2015). "Peripheral Neuropathies in Nonparetic Upper Extremities of Stroke Patients Induced by Excessive Use of a Walking Device." J Stroke Cerebrovasc Dis 24(8): 1841-1847.	PubMed_03_02_2017	no	0	no	0	0	0	0	0	exclusion; after scanning title & abstract (no; no)	1	no	0	no	0	b, c, e
124	Draicchio F, Trebbi M, Mari S, Forzano F, Serrao M, Sicklinger A, Silvetti A, Iavicoli S, Ranavolo A. Biomechanical evaluation of supermarket cashiers before and after a redesign of the checkout counter. Ergonomics. 2012;55(6):650-69. doi: 10.1080/00140139.2012.659762. PubMed PMID: 22455556.	added by Seidel	yes	1	yes	1	3	2	1	1	discussion necessary; DHS exclusion after scanning full-text, BS inclusion after scanning title & abstract	3	no	0	no	0	d
125	Dryson, E. W. and C. B. Walls (2001). "The distribution of occupations in two populations with upper limb pain." Int J Occup Environ Health 7(3): 201-205.	added by Seidel	no	0	no	0	0	0	0	0	exclusion; after scanning title & abstract (no; no)	1	no	0	no	0	c, e

126	East, J. M., Whittenmore, K., Polan, M., Lenehan, B. 2013.A fractured referral system-an audit of accident and emergency referrals to orthopedic fracture clinics at the University Hospital Limerick (UHL). Irish Journal of Medical Science 182:S57-S58 · March 2013	EMBASE_08_02_2017_Abstract_not found	no	0	no	0	0	0	0	0	exclusion; because abstract is missing	0	no	0	no	0	a
127	Edouard P, Depiesse F, Branco P, Alonso JM. Analyses of Helsinki 2012 European Athletics Championships injury and illness surveillance to discuss elite athletes risk factors. Clin J Sport Med. 2014 Sep;24(5):409-15.doi: 0.1097/JSM.0000000000000052. PubMed PMID: 24326930.	EMBASE_08_02_2017	no	0	no	0	0	0	0	0	exclusion; after scanning title & abstract (no; no)	1	no	0	no	0	b, c, e
128	Eijckelhof, B. H. W., Huysmans, M. A., Garza, J. B., Blatter, B. M., Van Dieën, J. H., Dennerlein, J. T., & Van Der Beek, A. J. (2013). The effects of workplace stressors on muscle activity in the neck-shoulder and forearm muscles during computer work: A systematic review and meta-analysis. European Journal of Applied Physiology, 113(12), 2897-2912.	added by Seidel	yes	1	yes	1	3	2	1	1	discussion necessary; DHS exclusion after scanning full-text, BS inclusion after scanning title & abstract	3	no	0	no	0	systematic review, no single study
129	El-Bestar, S. F., et al. (2011). "Neck-upper extremity musculoskeletal disorders among workers in the telecommunications company at Mansoura City." Int J Occup Saf Ergon 17(2): 195-205.	PubMed_03_02_2017	yes	1	yes	1	3	2	1	1	discussion necessary; DHS exclusion after scanning full-text, BS inclusion after scanning title & abstract	3	no	0	no	0	d
130	El-Sayed, A. M., et al. (2010). "Household food insecurity and symptoms of neurologic disorder in Ethiopia: an observational analysis." BMC Public Health 10: 802.	PubMed_03_02_2017	no	0	no	0	0	0	0	0	exclusion; after scanning title & abstract (no; no)	1	no	0	no	0	c, e
131	Eltayeb, S., Staal, J. B., Hassan, A., & De Bie, R. A. (2009). Work related risk factors for neck, shoulder and arms complaints: a cohort study among Dutch computer office workers. Journal of occupational rehabilitation, 19(4), 315-322.	added by Seidel	yes	1	yes	1	3	2	1	1	discussion necessary; DHS exclusion after scanning full-text, BS inclusion after scanning title & abstract	3	no	0	no	0	e
132	Emery, K. and J. N. Cote (2012). "Repetitive arm motion-induced fatigue affects shoulder but not endpoint position sense." Exp Brain Res 216(4): 553-564.	PubMed_03_02_2017	yes	1	no	0	1	1	0	0	exclusion; abstract (yes, no), full-text (no)	2	no	0	no	0	d
133	Erhan, B., et al. (2013). "Elbow problems in paraplegic spinal cord injured patients: frequency and related risk factors--a preliminary controlled study." Spinal Cord 51(5): 406-408.	PubMed_03_02_2017/ EMBASE_08_02_2017	no	0	no	0	0	0	0	0	exclusion; after scanning title & abstract (no; no)	1	no	0	no	0	b
134	Evans, Kevin D., and Carolyn Sommerich. "The feasibility of using an HCU system for investigating ergonomic injury among autoworkers." Journal of Diagnostic Medical Sonography 25.2 (2009): 80-87.	EMBASE_08_02_2017	yes	1	no	0	1	1	0	0	exclusion; abstract (yes, no), full-text (no)	2	no	0	no	0	c, e
135	Evans, P. J., et al. (2009). "Prevention and treatment of elbow stiffness." J Hand Surg Am 34(4): 769-778.	EMBASE_08_02_2017	no	0	no	0	0	0	0	0	exclusion; after scanning title & abstract (no; no)	1	no	0	no	0	c, e
136	Faber, A., Sell, L., Hansen, J. V., Burr, H., Lund, T., Holtermann, A., & Søgaard, K. (2012). Does muscle strength predict future musculoskeletal disorders and sickness absence?. Occupational medicine, 62(1), 41-46.	added by Seidel	yes	1	no	0	1	1	0	0	exclusion; abstract (yes, no), full-text (no)	2	no	0	no	0	c, e

137	Fan, Z. J., et al. (2014). "Predicting work-related incidence of lateral and medial epicondylitis using the strain index." Am J Ind Med 57(12): 1319-1330.	EMBASE_08_02_2017	yes	1	yes	1	3	2	2	1	inclusion for scanning full-text	5	no	0	no	0	e
138	Fan, Z. J., et al. (2014). "The association between combination of hand force and forearm posture and incidence of lateral epicondylitis in a working population." Hum Factors 56(1): 151-165.	EMBASE_08_02_2017	yes	1	yes	1	3	2	2	1	inclusion for scanning full-text	5	yes	1	yes	1	no
139	Fan, Z. J., et al. (2009). "Quantitative exposure-response relations between physical workload and prevalence of lateral epicondylitis in a working population." Am J Ind Med 52(6): 479-490.	PubMed_03_02_2017/ EMBASE_08_02_2017	yes	1	yes	1	3	2	2	1	inclusion for scanning full-text	5	yes	1	yes	1	no
140	Fearon, A., Scott, A., Ezzat, A., Kennedy, C., Levesque, L., Kahan, K., ... & Hoens, A. (2016). Engaging physiotherapists in effective knowledge translation–Treating tendinopathy effectively. Manual Therapy, 25, e9-e10.	EMBASE_08_02_2017_Abstract_not found	no	0	no	0	0	0	0	0	exclusion; because abstract is missing	0	no	0	no	0	a
141	Feathers, D. J., et al. (2013). "Alternative computer mouse designs: performance, posture, and subjective evaluations for college students aged 18-25." Work 44 Suppl 1: S115-122.	PubMed_03_02_2017	yes	1	no	0	1	1	1	1	discussion necessary	4	no	0	no	0	c, e
142	Fedorczyk, J. M. (2012). "Tendinopathies of the elbow, wrist, and hand: histopathology and clinical considerations." J Hand Ther 25(2): 191-200; quiz 201.	EMBASE_08_02_2017	no	0	no	0	0	0	0	0	exclusion; after scanning title & abstract (no; no)	1	no	0	no	0	c, e
143	Feleus, A., et al. (2008). "Incidence of non-traumatic complaints of arm, neck and shoulder in general practice." Man Ther 13(5): 426-433.	PubMed_03_02_2017	no	0	no	0	0	0	0	0	exclusion; after scanning title & abstract (no; no)	1	no	0	no	0	c, d, e
144	Fernandez-Carnero, J., et al. (2008). "Bilateral myofascial trigger points in the forearm muscles in patients with chronic unilateral lateral epicondylalgia: a blinded, controlled study." Clin J Pain 24(9): 802-807.	PubMed_03_02_2017	no	0	no	0	0	0	0	0	exclusion; after scanning title & abstract (no; no)	1	no	0	no	0	c, e
145	Fernandez-Carnero, J., et al. (2009). "Widespread mechanical pain hypersensitivity as sign of central sensitization in unilateral epicondylalgia: a blinded, controlled study." Clin J Pain 25(7): 555-561.	PubMed_03_02_2017	no	0	no	0	0	0	0	0	exclusion; after scanning title & abstract (no; no)	1	no	0	no	0	c, e
146	Fernandez-de-Las-Penas, C., et al. (2010). "Specific mechanical pain hypersensitivity over peripheral nerve trunks in women with either unilateral epicondylalgia or carpal tunnel syndrome." J Orthop Sports Phys Ther 40(11): 751-760.	PubMed_03_02_2017	no	0	no	0	0	0	0	0	exclusion; after scanning title & abstract (no; no)	1	no	0	no	0	c, e
147	Fethke NB, Merlino L, Gerr F. Effect of ergonomics training on agreement between expert and nonexpert ratings of the potential for musculoskeletal harm in manufacturing tasks. J Occup Environ Med. 2013 Dec;55(12 Suppl):S82-5. doi: 10.1097/JOM.0000000000000038. PubMed PMID: 24284748.	EMBASE_08_02_2017	no	0	no	0	0	0	0	0	exclusion; after scanning title & abstract (no; no)	1	no	0	no	0	c, d, e
148	Fieseler, G., et al. (2015). "Range of motion and isometric strength of shoulder joints of team handball athletes during the playing season, Part II: changes after midseason." J Shoulder Elbow Surg 24(3): 391-398.	PubMed_03_02_2017	no	0	no	0	0	0	0	0	exclusion; after scanning title & abstract (no; no)	1	no	0	no	0	b
149	Finestone HM, Yanni MM, Dalzell CJ. Patients' recall of diagnostic and treatment information improves with use of the Pain Explanation and Treatment Diagram in an outpatient chronic pain clinic. Pain Res Manag. 2015 May-Jun;20(3):145-51. PubMed PMID: 25831077; PubMed Central PMCID: PMC4447158.	EMBASE_08_02_2017	no	0	no	0	0	0	0	0	exclusion; after scanning title & abstract (no; no)	1	no	0	no	0	b

150	Fleisig, G. S. and J. R. Andrews (2012). "Prevention of elbow injuries in youth baseball pitchers." Sports Health 4(5): 419-424.	EMBASE_08_02_2017	no	0	no	0	0	0	0	0	exclusion; after scanning title & abstract (no; no)	1	no	0	no	0	b
151	Flouzat-Lachaniette, C. H., et al. (2012). "The natural progression of adult elbow osteonecrosis related to corticosteroid treatment." Clin Orthop Relat Res 470(12): 3478-3482.	PubMed_03_02_2017/ EMBASE_08_02_2017	no	0	no	0	0	0	0	0	exclusion; after scanning title & abstract (no; no)	1	no	0	no	0	b, c, e
152	Font YM, Castro-Santana LE, Nieves-Plaza M, Maldonado M, Mayor AM, Vilá LM. Factors associated with regional rheumatic pain disorders in a population of Puerto Ricans with diabetes mellitus. Clin Rheumatol. 2014 Jul;33(7):995-1000. doi: 10.1007/s10067-013-2474-1. PubMed PMID: 24522480; PubMed Central PMCID: PMC4302737.	EMBASE_08_02_2017	no	0	no	0	0	0	0	0	exclusion; after scanning title & abstract (no; no)	1	no	0	no	0	b, c, e
153	Forcella, L., Bonfiglioli, R., Cutilli, P. et al. Analysis of occupational stress in a high fashion clothing factory with upper limb biomechanical overload Int Arch Occup Environ Health (2012) 85: 527. doi:10.1007/s00420-011-0702-z	added by Seidel	yes	1	yes	1	3	2	1	1	discussion necessary; DHS exclusion after scanning full-text, BS inclusion after scanning title & abstract	3	no	0	no	0	b, d
154	Forman, J. L., Lopez-Valdes, F. J., Duprey, S., Bose, D., De Dios, E. D. P., Subit, D., ... & Segui-Gomez, M. (2015). The tolerance of the human body to automobile collision impact–a systematic review of injury biomechanics research, 1990–2009. Accident Analysis & Prevention, 80, 7-17.	added by Seidel	no	0	no	0	0	0	0	0	exclusion; after scanning title & abstract (no; no)	1	no	0	no	0	b, c, e
155	Foruria AM, Augustin S, Morrey BF, Sánchez-Sotelo J. Heterotopic ossification after surgery for fractures and fracture-dislocations involving the proximal aspect of the radius or ulna. J Bone Joint Surg Am. 2013 May 15;95(10):e66. doi: 10.2106/JBJS.K.01533. PubMed PMID: 23677367.	EMBASE_08_02_2017	no	0	no	0	0	0	0	0	exclusion; after scanning title & abstract (no; no)	1	no	0	no	0	b, c, e
156	Fotiadis DG, Fotiadou EG, Kokaridas DG, Mylonas AC. Prevalence of musculoskeletal disorders in professional symphony orchestra musicians in Greece: a pilot study concerning age, gender, and instrument-specific results. Med Probl Perform Art. 2013 Jun;28(2):91-5. PubMed PMID: 23752283.	EMBASE_08_02_2017	no	0	no	0	0	0	0	0	exclusion; after scanning title & abstract (no; no)	1	no	0	no	0	c, e
157	Franceschi, F., et al. (2014). "Obesity as a risk factor for tendinopathy: a systematic review." Int J Endocrinol 2014: 670262.	EMBASE_08_02_2017	no	0	no	0	0	0	0	0	exclusion; after scanning title & abstract (no; no)	1	no	0	no	0	c, e
158	Fraysse, F., et al. (2016). "Practices and risks associated with operation of tie-down lashings in the vehicle transport industry." Ergonomics 59(12): 1661-1672.	added by Seidel	yes	1	yes	1	3	2	2	1	inclusion for scanning full-text	5	no	0	no	0	d
159	Frazao, P., et al. (2010). "Risks associated with tendinitis: effects from demographic, socioeconomic, and psychological status among Brazilian workers." Am J Ind Med 53(1): 72-79.	PubMed_03_02_2017	no	0	no	0	0	0	0	0	exclusion; after scanning title & abstract (no; no)	1	no	0	no	0	c, e
160	Fronek, J., et al. (2015). "Shoulder functional performance status of Minor League professional baseball pitchers." J Shoulder Elbow Surg 24(1): 17-23.	PubMed_03_02_2017	no	0	no	0	0	0	0	0	exclusion; after scanning title & abstract (no; no)	1	no	0	no	0	b, c, e
161	Frost R, MacPherson H, O'Meara S. A critical scoping review of external uses of comfrey (Symphytum spp.). Complement Ther Med. 2013 Dec;21(6):724-45. doi: 10.1016/j.ctim.2013.09.009. Review. PubMed PMID: 24280482.	EMBASE_08_02_2017	no	0	no	0	0	0	0	0	exclusion; after scanning title & abstract (no; no)	1	no	0	no	0	b, c, d, e

162	Furushima K, Itoh Y, Iwabu S. What are the Risk Factors for Failure after Conservative Treatment of Ulnar Collateral Ligament Injuries of the Elbow in Baseball Players? Orthopaedic Journal of Sports Medicine. 2013;1(4 Suppl):2325967113S00015. doi:10.1177/2325967113S00015.	EMBASE_08_02_2017	no	0	no	0	0	0	0	0	exclusion; after scanning title & abstract (no; no)	1	no	0	no	0	b, c, d, e
163	Furushima, K., et al. (2014). "Classification of Olecranon Stress Fractures in Baseball Players." Am J Sports Med 42(6): 1343-1351.	PubMed_03_02_2017	no	0	no	0	0	0	0	0	exclusion; after scanning title & abstract (no; no)	1	no	0	no	0	b
164	Furuya, S., et al. (2012). "Individual differences in the biomechanical effect of loudness and tempo on upper-limb movements during repetitive piano keystrokes." Hum Mov Sci 31(1): 26-39.	PubMed_03_02_2017	yes	1	no	0	1	1	0	0	exclusion; abstract (yes, no), full-text (no)	2	no	0	no	0	d, e
165	Gallagher, S., & Heberger, J. R. (2013). Examining the Interaction of Force and Repetition on Musculoskeletal Disorder Risk A Systematic Literature Review. Human Factors: The Journal of the Human Factors and Ergonomics Society, 55(1), 108-124.	added by Seidel	yes	1	yes	1	3	2	1	1	discussion necessary; DHS exclusion after scanning full-text, BS inclusion after scanning title & abstract	3	no	0	no	0	d
166	Gallo, R., & Mazzetto, F. (2013). Ergonomic analysis for the assessment of the risk of work-related musculoskeletal disorder in forestry operations. Journal of Agricultural Engineering, 44(2s).	added by Seidel	yes	1	yes	1	3	2	1	1	discussion necessary; DHS exclusion after scanning full-text, BS inclusion after scanning title & abstract	3	no	0	no	0	d
167	Ganiyu, S. O., Olabode, J. A., Stanley, M. M., & Muhammad, I. (2015). Patterns of occurrence of work-related musculoskeletal disorders and its correlation with ergonomic hazards among health care professionals. Nigerian Journal of Experimental and Clinical Biosciences, 3(1), 18.	added by Seidel	yes	1	yes	1	3	2	1	1	discussion necessary; DHS exclusion after scanning full-text, BS inclusion after scanning title & abstract	3	no	0	no	0	e
168	Gardner, B. T., et al. (2016). "Functional Measures Developed for Clinical Populations Identified Impairment Among Active Workers with Upper Extremity Disorders." J Occup Rehabil 26(1): 84-94.	added by Seidel	no	0	yes	1	2	1	1	1	discussion necessary; DHS exclusion after scanning full-text, BS inclusion after scanning title & abstract	3	no	0	no	0	e
169	Garg, A., et al. (2012). "The WISTAH hand study: a prospective cohort study of distal upper extremity musculoskeletal disorders." BMC Musculoskelet Disord 13: 90.	EMBASE_08_02_2017	yes	1	yes	1	3	2	2	1	inclusion for scanning full-text	5	yes	1	no	0	e
170	Garg, A., et al. (2014). "The strain index and TLV for HAL: risk of lateral epicondylitis in a prospective cohort." Am J Ind Med 57(3): 286-302.	PubMed_03_02_2017/ EMBASE_08_02_2017	yes	1	yes	1	3	2	2	1	inclusion for scanning full-text	5	no	0	no	0	e
171	Gaspar, Ana Teresa, and Filipe Antunes. "Type I complex regional pain syndrome." Acta Médica Portuguesa 24.6 (2011): 1031-40.	EMBASE_08_02_2017	no	0	no	0	0	0	0	0	exclusion; after scanning title & abstract (no; no)	1	no	0	no	0	b, c, d, e
172	Gates, D. H., et al. (2016). "Range of Motion Requirements for Upper-Limb Activities of Daily Living." Am J Occup Ther 70(1): 7001350010p7001350011-7001350010p7001350010.	PubMed_03_02_2017	no	0	no	0	0	0	0	0	exclusion; after scanning title & abstract (no; no)	1	no	0	no	0	b, c, d, e

173	Gerr, F., Fethke, N. B., Merlino, L., Anton, D., Rosecrance, J., Jones, M. P., ... & Meyers, A. R. (2014). A prospective study of musculoskeletal outcomes among manufacturing workers: I. Effects of physical risk factors. Human factors, 56(1), 112-130.	added by Seidel	yes	1	yes	1	3	2	2	1	inclusion for scanning full-text	5	no	0	no	0	e
174	Gold, J. E., et al. (2009). "Specific and non-specific upper extremity musculoskeletal disorder syndromes in automobile manufacturing workers." Am J Ind Med 52(2): 124-132.	PubMed_03_02_2017	no	0	no	0	0	0	0	0	exclusion; after scanning title & abstract (no; no)	1	no	0	no	0	c, e
175	González-Moreno P, Sanabria RS, Hernández-Sánchez R, et al A6.12 Lateral Epicondyle Tendon Lesions Treatment with Platelet Growth Factors Annals of the Rheumatic Diseases 2013;72:A46.	EMBASE_08_02_2017	no	0	no	0	0	0	0	0	exclusion; after scanning title & abstract (no; no)	1	no	0	no	0	b, c, e
176	Grantham, W. J., et al. (2015). "The curveball as a risk factor for injury: a systematic review." Sports Health 7(1): 19-26.	added by Seidel	no	0	no	0	0	0	0	0	exclusion; after scanning title & abstract (no; no)	1	no	0	no	0	b
177	Green, H. J., et al. (2015). "Cellular properties of extensor carpi radialis brevis and trapezius muscles in healthy males and females." Can J Physiol Pharmacol 93(11): 953-966.	PubMed_03_02_2017	no	0	no	0	0	0	0	0	exclusion; after scanning title & abstract (no; no)	1	no	0	no	0	b, c, d, e
178	Grieve, J. R., & Dickerson, C. R. (2008). Overhead work: Identification of evidence-based exposure guidelines. Occupational Ergonomics, 8(1), 53-66.	added by Seidel	yes	1	no	0	1	1	1	1	discussion necessary	4	no	0	no	0	d
179	Gross, D. P. and M. C. Battie (2010). "Recovery expectations predict recovery in workers with back pain but not other musculoskeletal conditions." J Spinal Disord Tech 23(7): 451-456.	PubMed_03_02_2017	no	0	no	0	0	0	0	0	exclusion; after scanning title & abstract (no; no)	1	no	0	no	0	c, e
180	Grzywacz, J. G., et al. (2012). "Work organization and musculoskeletal health: clinical findings from immigrant Latino poultry processing and other manual workers." J Occup Environ Med 54(8): 995-1001.	PubMed_03_02_2017/ EMBASE_08_02_2017	yes	1	yes	1	3	2	2	1	inclusion for scanning full-text	5	yes	1	no	0	d
181	Guillin, R., et al. (2012). "Imaging of snapping phenomena." Br J Radiol 85(1018): 1343-1353.	EMBASE_08_02_2017	no	0	no	0	0	0	0	0	exclusion; after scanning title & abstract (no; no)	1	no	0	no	0	b, c, d, e
182	Gupta, A., et al. (2014). "Ergonomics in dentistry." Int J Clin Pediatr Dent 7(1): 30-34.	added by Seidel	no	0	no	0	0	0	0	0	exclusion; after scanning title & abstract (no; no)	1	no	0	no	0	c, e
183	Ha, C., et al. (2009). "The French Musculoskeletal Disorders Surveillance Program: Pays de la Loire network." Occup Environ Med 66(7): 471-479.	PubMed_03_02_2017	yes	1	no	0	1	1	0	0	exclusion; abstract (yes, no), full-text (no)	2	no	0	no	0	c, e
184	Hagberg, M., Vilhemsson, R., Tornqvist, E. W., & Toomingas, A. (2007). Incidence of self-reported reduced productivity owing to musculoskeletal symptoms: association with workplace and individual factors among computer users. Ergonomics, 50(11), 1820-1834.	added by Seidel	yes	1	yes	1	3	2	2	1	inclusion for scanning full-text	5	yes	1	no	0	d
185	Hansson, G. Å., Balogh, I., Ohlsson, K., Granqvist, L., Nordander, C., Arvidsson, I., ... & Skerfving, S. (2010). Physical workload in various types of work: Part II. Neck, shoulder and upper arm. International Journal of Industrial Ergonomics, 40(3), 267-281.	added by Seidel	yes	1	yes	1	3	2	1	1	discussion necessary; DHS exclusion after scanning full-text, BS inclusion after scanning title & abstract	3	no	0	no	0	d
186	Harber, P., Lin, B. 2011. Lexical Analysis Of Abstracts Demonstrates Occupational Health Perspective. DOI: 10.1164/ajrccm-conference.2011.183.1_MeetingAbstracts.A4775 Conference: American	EMBASE_08_02_2017_Abstract_not found	no	0	no	0	0	0	0	0	exclusion; because abstract is missing	0	no	0	no	0	a

	Thoracic Society 2011 International Conference, May 13-18, 2011 Denver Colorado																
187	Harrington, J. M., et al. (1998). "Surveillance case definitions for work related upper limb pain syndromes." Occup Environ Med 55(4): 264-271.	added by Seidel	no	0	no	0	0	0	0	0	exclusion; after scanning title & abstract (no; no)	1	no	0	no	0	c, e
188	Heales, L. J., Bergin, M. J., Vicenzino, B., & Hodges, P. W. (2016). Forearm muscle activity in lateral epicondylalgia: a systematic review with quantitative analysis. Sports Medicine, 46(12), 1833-1845.	added by Seidel	yes	1	no	0	1	1	1	1	discussion necessary	4	no	0	no	0	c, e
189	Healy, K. A., et al. (2011). "Hand problems among endourologists." J Endourol 25(12): 1915-1920.	EMBASE_08_02_2017	no	0	no	0	0	0	0	0	exclusion; after scanning title & abstract (no; no)	1	no	0	no	0	d
190	Hegmann, K. T., et al. (2014). "Impacts of differences in epidemiological case definitions on prevalence for upper-extremity musculoskeletal disorders." Hum Factors 56(1): 191-202.	added by Seidel	no	0	no	0	0	0	0	0	exclusion; after scanning title & abstract (no; no)	1	no	0	no	0	c, e
191	Helliwell, P. S., et al. (2003). "Towards epidemiological criteria for soft-tissue disorders of the arm." Occup Med (Lond) 53(5): 313-319.	added by Seidel	yes	1	no	0	1	1	0	0	exclusion; abstract (yes, no), full-text (no)	2	no	0	no	0	c, e
192	Herquelot, E., et al. (2013). "Work-related risk factors for lateral epicondylitis and other cause of elbow pain in the working population." Am J Ind Med 56(4): 400-409.	PubMed_03_02_2017/ EMBASE_08_02_2017	yes	1	yes	1	3	2	2	1	inclusion for scanning full-text	5	yes	1	yes	1	no
193	Herquelot, E., et al. (2013). "Work-related risk factors for incidence of lateral epicondylitis in a large working population." Scand J Work Environ Health 39(6): 578-588.	PubMed_03_02_2017/ EMBASE_08_02_2017	yes	1	yes	1	3	2	2	1	inclusion for scanning full-text	5	yes	1	yes	1	no
194	Hibberd, E. E., et al. (2015). "Optimal management of ulnar collateral ligament injury in baseball pitchers." Open Access J Sports Med 6: 343-352.	added by Seidel	no	0	no	0	0	0	0	0	exclusion; after scanning title & abstract (no; no)	1	no	0	no	0	d
195	Hoe VCW, Urquhart DM, Kelsall HL, Sim MR. Ergonomic design and training for preventing work-related musculoskeletal disorders of the upper limb and neck in adults. Cochrane Database of Systematic Reviews 2012, Issue 8. Art. No.: CD008570. DOI: 10.1002/14651858.CD008570.pub2	CochraneWork_06_01_2017	yes	1	no	0	1	1	1	1	discussion necessary	4	no	0	no	0	c, d, e
196	Holtermann, A., Blangsted, A. K., Christensen, H., Hansen, K., & Søgaard, K. (2009). What characterizes cleaners sustaining good musculoskeletal health after years with physically heavy work?. International archives of occupational and environmental health, 82(8), 1015.	added by Seidel	yes	1	yes	1	3	2	1	1	discussion necessary; DHS exclusion after scanning full-text, BS inclusion after scanning title & abstract	3	no	0	no	0	e
197	Hong CC, Nashi N, Hey HW, Chee YH, Murphy D. Clinically relevant heterotopic ossification after elbow fracture surgery: a risk factors study. Orthop Traumatol Surg Res. 2015 Apr;101(2):209-13. doi: 10.1016/j.otsr.2014.10.021. PubMed PMID: 25701160.	EMBASE_08_02_2017	no	0	no	0	0	0	0	0	exclusion; after scanning title & abstract (no; no)	1	no	0	no	0	b, c, d, e
198	Hoozemans MJ, Knelange EB, Frings-Dresen MH, Veeger HE, Kuijer PP. Are pushing and pulling work-related risk factors for upper extremity symptoms? A systematic review of observational studies. Occup Environ Med. 2014 Nov;71(11):788-95. doi: 10.1136/oemed-2013-101837. Review. PubMed PMID: 25035115.	EMBASE_08_02_2017	yes	1	yes	1	3	2	1	1	discussion necessary; DHS exclusion after scanning full-text, BS inclusion after	3	no	0	no	0	e

											scanning title & abstract						
199	Hou WH, Chi CC, Lo HLD, Kuo KN, Chuang HY. Vocational rehabilitation for enhancing return-to-work in workers with traumatic upper limb injuries. Cochrane Database of Systematic Reviews 2013, Issue 10. Art. No.: CD010002. DOI: 10.1002/14651858.CD010002.pub2	CochraneWork_06_01_2017	no	0	no	0	0	0	0	0	exclusion; after scanning title & abstract (no; no)	1	no	0	no	0	c, e
200	House R, Krajnak K, Jiang D. Factors affecting finger and hand pain in workers with HAVS. Occupational medicine (Oxford, England) 2016;66(4):292-5. doi: 10.1093/occmed/kqw022 [published Online First: 2016/03/02]	PubMed_03_02_2017	no	0	no	0	0	0	0	0	exclusion; after scanning title & abstract (no; no)	1	no	0	no	0	d, e
201	House R, Wills M, Liss G, et al. The effect of hand-arm vibration syndrome on quality of life. Occupational medicine (Oxford, England) 2014;64(2):133-5. doi: 10.1093/occmed/kqt167 [published Online First: 2014/02/04]	PubMed_03_02_2017	no	0	no	0	0	0	0	0	exclusion; after scanning title & abstract (no; no)	1	no	0	no	0	d, e
202	Horn, S., et al. (2009). "Self-reported anabolic-androgenic steroids use and musculoskeletal injuries: findings from the center for the study of retired athletes health survey of retired NFL players." Am J Phys Med Rehabil 88(3): 192-200.	PubMed_03_02_2017	no	0	no	0	0	0	0	0	exclusion; after scanning title & abstract (no; no)	1	no	0	no	0	b, c, d, e
203	Hsu, J. W., et al. (2009). "The emerging role of elbow arthroscopy in chronic use injuries and fracture care." Hand Clin 25(3): 305-321.	EMBASE_08_02_2017_Abstract_not found	no	0	no	0	0	0	0	0	exclusion; because abstract is missing	0	no	0	no	0	a
204	Hsu, D. J., Chang, J. H., Wu, J. D., Chen, C. Y., & Yang, Y. H. (2011). Prevalence of musculoskeletal disorders and job exposure in Taiwan oyster shuckers. American journal of industrial medicine, 54(11), 885-893.	added by Seidel	yes	1	yes	1	3	2	2	1	inclusion for scanning full-text	5	yes	1	no	0	d
205	Huang CY, Cho CY (2011). The effects of dual task on posture and muscle activity of upper trunk in touch and non-touch typists. Proceedings of the 16th International Congress of the WCPT. Amsterdam, Holland: World Confederation for Physical Therapy.	EMBASE_08_02_2017_Abstract_not found	no	0	no	0	0	0	0	0	exclusion; because abstract is missing	0	no	0	no	0	a
206	Huang, Q., et al. (2015). "The arthropathic and functional impairment features of adult Kashin-Beck disease patients in Aba Tibetan area in China." Osteoarthritis Cartilage 23(4): 601-606.	PubMed_03_02_2017	no	0	no	0	0	0	0	0	exclusion; after scanning title & abstract (no; no)	1	no	0	no	0	d
207	Huysmans, M. A., IJmker, S., Blatter, B. M., Knol, D. L., van Mechelen, W., Bongers, P. M., & van der Beek, A. J. (2012). The relative contribution of work exposure, leisure time exposure, and individual characteristics in the onset of arm–wrist–hand and neck–shoulder symptoms among office workers. International archives of occupational and environmental health, 85(6), 651-666.	added by Seidel	yes	1	yes	1	3	2	1	1	discussion necessary; DHS exclusion after scanning full-text, BS inclusion after scanning title & abstract	3	no	0	no	0	d, e
208	Ihm, J. (2008). "Proximal wrist extensor tendinopathy." Curr Rev Musculoskelet Med 1(1): 48-52.	added by Seidel	yes	1	no	0	1	1	0	0	exclusion; abstract (yes, no), full-text (no)	2	no	0	no	0	c, e
209	İLÇE, Arzu. "STUDY ON WORK-RELATED MUSCULOSKELETAL DISORDERS IN INTENSIVE CARE UNIT NURSES." Anatolian Journal of Clinical Investigation 8.2 (2014).	EMBASE_08_02_2017	no	0	no	0	0	0	0	0	exclusion; after scanning title & abstract (no; no)	1	no	0	no	0	c, e

210	Inbaraj LR, Haebar OJ, Saj F, Dawson S, Paul P, Prabhakar AK, Mohan VR, Alex RG. Prevalence of musculoskeletal disorders among brick kiln workers in rural Southern India. Indian J Occup Environ Med. 2013 May;17(2):71-5. doi: 10.4103/0019-5278.123170. PubMed PMID: 24421594; PubMed Central PMCID: PMC3877450.	EMBASE_08_02_2017	no	0	yes	1	2	1	1	1	discussion necessary	4	no	0	no	0	d
211	Jacobs, J. W. and J. W. Bijlsma (2011). "Glucocorticoids in rheumatology: indications and routes of administration." Clin Exp Rheumatol 29(5 Suppl 68): S81-84.	EMBASE_08_02_2017	no	0	no	0	0	0	0	0	exclusion; after scanning title & abstract (no; no)	1	no	0	no	0	b, c, e
212	Jain, N. B., et al. (2011). "Prevalence of and factors associated with posterior tibial tendon pathology on sonographic assessment." Pm r 3(11): 998-1004.	PubMed_03_02_2017	no	0	no	0	0	0	0	0	exclusion; after scanning title & abstract (no; no)	1	no	0	no	0	d
213	James M. Paci, MD, Christopher Michael Jones, MD, Jie Yang, PhD, Jiawen Zhu, MS, David Komatsu, PhD, Arturo Flores, MS, ATC, David Van Dyke, M.S., S.C.C.C., C.S. Predictive Value Of Preseason Screening In Collegiate Baseball Pitchers. Orthopaedic Journal of Sports Medicine Vol 3, Issue 7_suppl2 First published date: July-17-2015 10.1177/2325967115S00148	EMBASE_08_02_2017	no	0	no	0	0	0	0	0	exclusion; after scanning title & abstract (no; no)	1	no	0	no	0	b
214	Janga, D. and O. Akinfenwa (2012). "Work-related repetitive strain injuries amongst practitioners of obstetric and gynaecological ultrasound worldwide." Arch Gynecol Obstet 286(2): 353-356.	added by Seidel	yes	1	yes	1	3	2	1	1	discussion necessary; DHS exclusion after scanning full-text, BS inclusion after scanning title & abstract	3	no	0	no	0	e
215	Jensen, A., et al. (2008). "Locomotor diseases among male long-haul truck drivers and other professional drivers." Int Arch Occup Environ Health 81(7): 821-827.	PubMed_03_02_2017	yes	1	no	0	1	1	0	0	exclusion; abstract (yes, no), full-text (no)	2	no	0	no	0	c, e
216	John D. Collins, Leonard W. O'Sullivan, Musculoskeletal disorder prevalence and psychosocial risk exposures by age and gender in a cohort of office based employees in two academic institutions, International Journal of Industrial Ergonomics, Volume 46, March 2015, Pages 85-97, ISSN 0169-8141, http://dx.doi.org/10.1016/j.ergon.2014.12.013.	EMBASE_08_02_2017	no	0	no	0	0	0	0	0	exclusion; after scanning title & abstract (no; no)	1	no	0	no	0	c, e
217	Johnston, V. (2007). Biological pathways between occupational stress and work-related musculoskeletal disorders of the neck and upper extremity. Physical Therapy Reviews, 12(4), 335-345.	added by Seidel	no	0	yes	1	2	1	1	1	discussion necessary	4	no	0	no	0	d
218	Jonathan Dickens, Kevin F. Wilson, Scott Tintle, Reed Heckert, Benjamin K. Potter, Outcomes and Risk Factors for Poor Functional Outcomes in Open Elbow Fractures, The Journal of Hand Surgery, Volume 37, Issue 8, 2012, Page 33, ISSN 0363-5023, http://dx.doi.org/10.1016/S0363-5023(12)60044-6. (http://www.sciencedirect.com/science/article/pii/S0363502312600446)	EMBASE_08_02_2017_Abstract_not found	no	0	no	0	0	0	0	0	exclusion; because abstract is missing	0	no	0	no	0	a
219	Jones, K. J., et al. (2013). "Functional outcomes following revision ulnar collateral ligament reconstruction in Major League Baseball pitchers." J Shoulder Elbow Surg 22(5): 642-646.	EMBASE_08_02_2017	no	0	no	0	0	0	0	0	exclusion; after scanning title & abstract (no; no)	1	no	0	no	0	c, d, e
220	Jones, T., & Kumar, S. (2007). Assessment of physical demands and comparison of multiple exposure definitions in a repetitive sawmill job: board edger operator. Ergonomics, 50(5), 676-693.	added by Seidel	yes	1	yes	1	3	2	2	1	inclusion for scanning full-text	5	yes	1	no	0	e

221	Ju, Y. Y., et al. (2011). "Work-related musculoskeletal disorders in athletic trainer." J Occup Rehabil 21(2): 190-198.	PubMed_03_02_2017	no	0	yes	1	2	1	1	1	discussion necessary	4	no	0	no	0	e
222	Juratli, S. M., et al. (2010). "A population-based study of ulnar neuropathy at the elbow in Washington State workers' compensation." Am J Ind Med 53(12): 1242-1251.	PubMed_03_02_2017	no	0	no	0	0	0	0	0	exclusion; after scanning title & abstract (no; no)	1	no	0	no	0	c, e
223	Kaerlev, L., et al. (2008). "Hospital contacts for injuries and musculoskeletal diseases among seamen and fishermen: a population-based cohort study." BMC Musculoskelet Disord 9: 8.	PubMed_03_02_2017	no	0	no	0	0	0	0	0	exclusion; after scanning title & abstract (no; no)	1	no	0	no	0	c, e
224	Kaliniene G, Ustinaviciene R, Skemiene L, Vaiciulis V, Vasilavicius P. Associations between musculoskeletal pain and work-related factors among public service sector computer workers in Kaunas County, Lithuania. BMC Musculoskelet Disord. 2016 Oct 7;17(1):420. PubMed PMID: 27717347; PubMed Central PMCID: PMC5055679.	EMBASE_08_02_2017	yes	1	yes	1	3	2	2	1	inclusion for scanning full-text	5	yes	1	no	0	e
225	Kameyama, M., et al. (2009). "The presence of limited joint mobility is significantly associated with multiple digit involvement by stenosing flexor tenosynovitis in diabetics." J Rheumatol 36(8): 1686-1690.	PubMed_03_02_2017	no	0	no	0	0	0	0	0	exclusion; after scanning title & abstract (no; no)	1	no	0	no	0	c, e
226	Kamper, S. J., et al. (2010). "Treatment-based subgroups of low back pain: a guide to appraisal of research studies and a summary of current evidence." Best Pract Res Clin Rheumatol 24(2): 181-191.	EMBASE_08_02_2017	no	0	no	0	0	0	0	0	exclusion; after scanning title & abstract (no; no)	1	no	0	no	0	d
227	Kane, S. F., et al. (2014). "Evaluation of elbow pain in adults." Am Fam Physician 89(8): 649-657.	PubMed_03_02_2017	yes	1	no	0	1	1	1	1	discussion necessary	4	no	0	no	0	c, e
228	Kelley, Brian P., and Kevin C. Chung. "Soft-Tissue Coverage for Elbow Trauma." Hand clinics 31.4 (2015): 693-703.	EMBASE_08_02_2017_Abstract_not found	no	0	no	0	0	0	0	0	exclusion; because abstract is missing	0	no	0	no	0	a
229	Kennedy, C. A., Amick III, B. C., Dennerlein, J. T., Brewer, S., Catli, S., Williams, R., ... & Franzblau, A. (2010). Systematic review of the role of occupational health and safety interventions in the prevention of upper extremity musculoskeletal symptoms, signs, disorders, injuries, claims and lost time. Journal of occupational rehabilitation, 20(2), 127-162.	added by Seidel	no	0	no	0	0	0	0	0	exclusion; after scanning title & abstract (no; no)	1	no	0	no	0	c, e
230	Kim, C. Y., et al. (2014). "Effect of Treatment Table Height on Shoulder Muscles during Ultrasound Therapy Work." J Phys Ther Sci 26(10): 1615-1617.	added by Seidel	no	0	no	0	0	0	0	0	exclusion; after scanning title & abstract (no; no)	1	no	0	no	0	d
231	Kim, T. H., et al. (2013). "Joint laxity negatively correlates with lumbar disc degeneration in young adults." Spine (Phila Pa 1976) 38(24): E1541-1547.	PubMed_03_02_2017	no	0	no	0	0	0	0	0	exclusion; after scanning title & abstract (no; no)	1	no	0	no	0	d
232	Kachooei AR, Claessen FM, Chase SM, Verheij KK, van Dijk CN, Ring D. Factors associated with removal of a radial head prosthesis placed for acute trauma. Injury. 2016 Jun;47(6):1253-7. doi: 10.1016/j.injury.2016.02.023. PubMed PMID: 26975795.	EMBASE_08_02_2017	no	0	no	0	0	0	0	0	exclusion; after scanning title & abstract (no; no)	1	no	0	no	0	b, d
233	Kaila-Kangas L, Haukka E, Miranda H, Kivekäs T, Ahola K, Luukkonen R, Shiri R, Kääriä S, Heliövaara M, Leino-Arjas P. Common mental and musculoskeletal disorders as predictors of disability retirement among Finns. J Affect Disord. 2014 Aug;165:38-44. doi: 10.1016/j.jad.2014.04.036. PubMed PMID: 24882175.	EMBASE_08_02_2017	yes	1	no	0	1	1	1	1	discussion necessary	4	no	0	no	0	c, e

234	Kebede Deyyas W, Tafese A. Environmental and organizational factors associated with elbow/forearm and hand/wrist disorder among sewing machine operators of garment industry in Ethiopia. J Environ Public Health. 2014;2014:732731. doi: 10.1155/2014/732731. PubMed PMID: 25298780; PubMed Central PMCID: PMC4178914.	EMBASE_08_02_2017	yes	1	yes	1	3	2	2	1	inclusion for scanning full-text	5	no	0	no	0	d
235	Klussmann, A., et al. (2010). "The Key Indicator Method for Manual Handling Operations (KIM-MHO) - evaluation of a new method for the assessment of working conditions within a cross-sectional study." BMC Musculoskelet Disord 11: 272.	EMBASE_08_02_2017	yes	1	yes	1	3	2	2	1	inclusion for scanning full-text	5	no	0	no	0	e
236	Knobloch K. Drug-Induced Tendon Disorders. Adv Exp Med Biol. 2016;920:229-38. doi: 10.1007/978-3-319-33943-6_22. PubMed PMID: 27535265.	EMBASE_08_02_2017	no	0	no	0	0	0	0	0	exclusion; after scanning title & abstract (no; no)	1	no	0	no	0	b, d
237	Kobayashi, T., et al. (2014). "Prevalence of and risk factors for shoulder osteoarthritis in Japanese middle-aged and elderly populations." J Shoulder Elbow Surg 23(5): 613-619.	PubMed_03_02_2017	no	0	no	0	0	0	0	0	exclusion; after scanning title & abstract (no; no)	1	no	0	no	0	b, d
238	Koh, K. H., et al. (2013). "Surgical treatment of elbow stiffness caused by post-traumatic heterotopic ossification." J Shoulder Elbow Surg 22(8): 1128-1134.	EMBASE_08_02_2017	no	0	no	0	0	0	0	0	exclusion; after scanning title & abstract (no; no)	1	no	0	no	0	b, c, d, e
239	Kotani, K., Barrero, L. H., Lee, D. L., & Dennerlein, J. T. (2007). Effect of horizontal position of the computer keyboard on upper extremity posture and muscular load during computer work. Ergonomics, 50(9), 1419-1432.	added by Seidel	no	0	yes	1	2	1	1	1	discussion necessary	4	no	0	no	0	d
240	Krabben, A., et al. (2014). "MRI-detected subclinical joint inflammation is associated with radiographic progression." Ann Rheum Dis 73(11): 2034-2037.	PubMed_03_02_2017	no	0	no	0	0	0	0	0	exclusion; after scanning title & abstract (no; no)	1	no	0	no	0	b, c, e
241	Kreiswirth, E. M., et al. (2014). "Incidence of injury among male Brazilian jiujitsu fighters at the World Jiu-Jitsu No-Gi Championship 2009." J Athl Train 49(1): 89-94.	PubMed_03_02_2017	no	0	no	0	0	0	0	0	exclusion; after scanning title & abstract (no; no)	1	no	0	no	0	b, d
242	Krogue JD, Aleem AW, Osei DA, Goldfarb CA, Calfee RP. Predictors of surgical revision after in situ decompression of the ulnar nerve. J Shoulder Elbow Surg. 2015 Apr;24(4):634-9. doi: 10.1016/j.jse.2014.12.015. Erratum in: J Shoulder Elbow Surg. 2015 Jun;24(6):994. PubMed PMID: 25660241.	EMBASE_08_02_2017	no	0	no	0	0	0	0	0	exclusion; after scanning title & abstract (no; no)	1	no	0	no	0	b, c, e
243	Krüger, M. and E. Bischof-Leger (2008). "[Frequency of biceps tendon tenosynovitis from crutches: a sonographical observation]." Z Rheumatol 67(1): 62, 64-67.	PubMed_03_02_2017	no	0	no	0	0	0	0	0	exclusion; after scanning title & abstract (no; no)	1	no	0	no	0	b, c, e
244	Kryger, A. I., et al. (2007). "The role of physical examinations in studies of musculoskeletal disorders of the elbow." Occup Environ Med 64(11): 776-781.	PubMed_03_02_2017	no	0	no	0	0	0	0	0	exclusion; after scanning title & abstract (no; no)	1	no	0	no	0	c, e
245	Kumar, A. R., et al. (2009). "Lessons from the modern battlefield: successful upper extremity injury reconstruction in the subacute period." J Trauma 67(4): 752-757.	EMBASE_08_02_2017_Abstract_not found	no	0	no	0	0	0	0	0	exclusion; because abstract is missing	0	no	0	no	0	a
246	Kumar MS, Goud BR, Joseph B. A study of occupational health and safety measures in the Laundry Department of a private tertiary care teaching hospital, Bengaluru. Indian J Occup Environ Med. 2014 Jan;18(1):13-20. doi: 10.4103/0019-5278.134951. PubMed PMID: 25006311; PubMed Central PMCID: PMC4083516.	EMBASE_08_02_2017	yes	1	no	0	1	1	1	1	discussion necessary	4	no	0	no	0	c, d, e

247	Laffont, I., Julia, M., Tiffreau, V., Yelnik, A., Herisson, C., & Pelissier, J. (2010). Aging and sequelae of poliomyelitis. Annals of physical and rehabilitation medicine, 53(1), 24-33.	EMBASE_08_02_2017	no	0	no	0	0	0	0	0	exclusion; after scanning title & abstract (no; no)	1	no	0	no	0	b, c, d, e
248	Langer, D., et al. (2015). "Stenosing flexor tenosynovitis: Validity of standard assessment tools of daily functioning and quality of life." J Hand Ther 28(4): 384-387; quiz 388.	PubMed_03_02_2017	no	0	no	0	0	0	0	0	exclusion; after scanning title & abstract (no; no)	1	no	0	no	0	b, c, e
249	Larkin, M. E., et al. (2014). "Musculoskeletal complications in type 1 diabetes." Diabetes Care 37(7): 1863-1869.	PubMed_03_02_2017	no	0	no	0	0	0	0	0	exclusion; after scanning title & abstract (no; no)	1	no	0	no	0	b, c, d, e
250	Lauren H. Redler, Joshua S. Dines, Elbow Trauma in the Athlete, Hand Clinics, Volume 31, Issue 4, November 2015, Pages 663-681, ISSN 0749-0712, http://dx.doi.org/10.1016/j.hcl.2015.07.002.	EMBASE_08_02_2017_Abstract_not found	no	0	no	0	0	0	0	0	exclusion; because abstract is missing	0	no	0	no	0	a
251	Lee, J. T., et al. (2008). "Long term results of radial tunnel release--the effect of co-existing tennis elbow, multiple compression syndromes and workers' compensation." J Plast Reconstr Aesthet Surg 61(9): 1095-1099.	EMBASE_08_02_2017	no	0	no	0	0	0	0	0	exclusion; after scanning title & abstract (no; no)	1	no	0	no	0	c, e
252	Lee, S. Y., et al. (2016). "The Potential Risk Factors Relevant to Lateral Epicondylitis by Wrist Coupling Posture." PLoS One 11(5): e0155379.	EMBASE_08_02_2017	yes	1	yes	1	3	2	2	1	inclusion for scanning full-text	5	no	0	no	0	d
253	Levanon, Y., et al. (2010). "Validity and reliability of upper extremity three-dimensional kinematics during a typing task." Gait Posture 32(4): 469-474.	PubMed_03_02_2017	yes	1	no	0	1	1	1	1	discussion necessary	4	no	0	no	0	b, c, d, e
254	Lewis, Thomas L., Nicholas A. Ferran, and Benjamin WT Gooding. "Current concepts in shoulder and elbow fractures and dislocations." Surgery (Oxford) 34.3 (2016): 122-128	EMBASE_08_02_2017	no	0	no	0	0	0	0	0	exclusion; after scanning title & abstract (no; no)	1	no	0	no	0	b, c, d, e
255	Lindenhovius, A., et al. (2012). "Radiographic arthrosis after elbow trauma: interobserver reliability." J Hand Surg Am 37(4): 755-759.	EMBASE_08_02_2017	no	0	no	0	0	0	0	0	exclusion; after scanning title & abstract (no; no)	1	no	0	no	0	b, c, d, e
256	Lo, J. and J. A. Ashton-Miller (2008). "Effect of upper and lower extremity control strategies on predicted injury risk during simulated forward falls: a study in healthy young adults." J Biomech Eng 130(4): 041015.	PubMed_03_02_2017	yes	1	no	0	1	1	1	1	discussion necessary	4	no	0	no	0	b
257	Lo, M. Y. and M. R. Safran (2007). "Surgical treatment of lateral epicondylitis: a systematic review." Clin Orthop Relat Res 463: 98-106.	EMBASE_08_02_2017	no	0	no	0	0	0	0	0	exclusion; after scanning title & abstract (no; no)	1	no	0	no	0	b, c, e
258	Lombardi DA, Smith GS, Courtney TK, Brennan MJ, Kim JY, Perry MJ. Work-related falls from ladders--a follow-back study of US emergency department cases. Scand J Work Environ Health. 2011 Nov;37(6):525-32. doi: 10.5271/sjweh.3174. PubMed PMID: 21670941.	EMBASE_08_02_2017_Double	no	0	no	0	0	0	0	0	exclusion; after scanning title & abstract (no; no)	1	no	0	no	0	c, d, e
259	Lopez, V. G. (2011). "[Evaluation of the Sentinel Surveillance Program in Occupational Health in Navarra (1998-2007)]." An Sist Sanit Navar 34(3): 419-430.	EMBASE_08_02_2017	no	0	no	0	0	0	0	0	exclusion; after scanning title & abstract (no; no)	1	no	0	no	0	c, e
260	Lund, H., et al. (2008). "Movement detection impaired in patients with knee osteoarthritis compared to healthy controls: a cross-sectional case-control study." J Musculoskelet Neuronal Interact 8(4): 391-400.	PubMed_03_02_2017	no	0	no	0	0	0	0	0	exclusion; after scanning title & abstract (no; no)	1	no	0	no	0	d

261	Lutsky, K., et al. (2016). "Hand Dominance and Common Hand Conditions." Orthopedics 39(3): e444-448.	added by Seidel	yes	1	no	0	1	1	1	1	discussion necessary	4	no	0	no	0	c, e
262	MacKenzie, M. S. and J. Berkowitz (2010). "Do procedural skills workshops during family practice residency work?" Can Fam Physician 56(8): e296-301.	EMBASE_08_02_2017	no	0	no	0	0	0	0	0	exclusion; after scanning title & abstract (no; no)	1	no	0	no	0	d
263	MacIver, H., Smyth, G., & Bird, H. A. (2007). Occupational disorders: non-specific forearm pain. Best Practice & Research Clinical Rheumatology, 21(2), 349-365.	added by Seidel	no	0	yes	1	2	1	1	1	discussion necessary	4	no	0	no	0	e
264	Maffulli, N., Longo, U. G., Gougoulias, N., Caine, D., & Denaro, V. (2010). Sport injuries: a review of outcomes. British medical bulletin, ldq026.	EMBASE_08_02_2017	no	0	no	0	0	0	0	0	exclusion; after scanning title & abstract (no; no)	1	no	0	no	0	b, d
265	Mallows, A., Debenham, J., Walker, T., & Littlewood, C. (2016). Association of psychological variables and outcome in tendinopathy: a systematic review. British Journal of Sports Medicine, bjsports-2016.	added by Seidel	no	0	no	0	0	0	0	0	exclusion; after scanning title & abstract (no; no)	1	no	0	no	0	c, e
266	Marks, W., et al. (2014). "Humeral fracture in arm wrestling: bone morphology as a permanent risk factor. Indications for safety measures in arm wrestling." J Sports Med Phys Fitness 54(1): 88-92.	PubMed_03_02_2017	no	0	no	0	0	0	0	0	exclusion; after scanning title & abstract (no; no)	1	no	0	no	0	d
267	Marras, W. S., Cutlip, R. G., Burt, S. E., & Waters, T. R. (2009). National occupational research agenda (NORA) future directions in occupational musculoskeletal disorder health research. Applied ergonomics, 40(1), 15-22.	added by Seidel	yes	1	yes	1	3	2	2	1	inclusion for scanning full-text	5	no	0	no	0	e
268	Martimo K-P, Shiri R, Miranda H, Ketola R, Varonen H, Viikari-Juntura E. Self-reported productivity loss among workers with upper extremity disorders. Scand J Work Environ Health. 2009;35(4):301–308.	EMBASE_08_02_2017_Double	yes	1	yes	1	3	2	2	1	inclusion for scanning full-text	5	no	0	no	0	e
269	Martimo K-P, Shiri R, Miranda H, Ketola R, Varonen H, Viikari-Juntura E. Effectiveness of an ergonomic intervention on the productivity of workers with upper-extremity disorders – a randomized controlled trial. Scand J Work Environ Health. 2010;36(1):25–33	EMBASE_08_02_2017	no	0	no	0	0	0	0	0	exclusion; after scanning title & abstract (no; no)	1	no	0	no	0	c, e
270	Martin, B. D., et al. (2008). "Complications related to simple dislocations of the elbow." Hand Clin 24(1): 9-25.	EMBASE_08_02_2017	no	0	no	0	0	0	0	0	exclusion; after scanning title & abstract (no; no)	1	no	0	no	0	b, c, e
271	Marwa Amer; Tarek Shafshak; Mowaffak Saad. 2014.Upper limb neuro musculoskeletal complications in patients using walking aids. PM&R. Vol. 6, Iss. 8S2, 2014 S159	EMBASE_08_02_2017	no	0	no	0	0	0	0	0	exclusion; after scanning title & abstract (no; no)	1	no	0	no	0	b
272	Matzon, J. L., et al. (2016). "Risk Factors for Ulnar Nerve Instability Resulting in Transposition in Patients With Cubital Tunnel Syndrome." J Hand Surg Am 41(2): 180-183.	added by Seidel	no	0	no	0	0	0	0	0	exclusion; after scanning title & abstract (no; no)	1	no	0	no	0	c, e
273	Mattioli, S., et al. (2015). "Upper-extremity and neck disorders associated with keyboard and mouse use." Handb Clin Neurol 131: 427-433.	added by Seidel	no	0	yes	1	2	1	1	1	discussion necessary	4	no	0	no	0	d
274	Mautner BK, Blazuk J. Overuse throwing injuries in skeletally immature athletes--diagnosis, treatment, and prevention. Curr Sports Med Rep. 2015 May-Jun;14(3):209-14. doi: 10.1249/JSR.0000000000000155. PubMed PMID: 25968854.	EMBASE_08_02_2017	no	0	no	0	0	0	0	0	exclusion; after scanning title & abstract (no; no)	1	no	0	no	0	b, c, e

275	McMahon, P. J., et al. (2013). "Anterior shoulder dislocation increases the propensity for recurrence: a cadaveric study of the number of dislocations and type of capsulolabral lesion." J Shoulder Elbow Surg 22(8): 1046-1052.	PubMed_03_02_2017	no	0	no	0	0	0	0	0	exclusion; after scanning title & abstract (no; no)	1	no	0	no	0	b
276	Menta, R., Randhawa, K., Côté, P., Wong, J. J., Yu, H., Sutton, D., ... & Brown, C. (2015). The effectiveness of exercise for the management of musculoskeletal disorders and injuries of the elbow, forearm, wrist, and hand: a systematic review by the Ontario Protocol for Traffic Injury Management (OPTIMa) collaboration. Journal of manipulative and physiological therapeutics, 38(7), 507-520.	added by Seidel	no	0	no	0	0	0	0	0	exclusion; after scanning title & abstract (no; no)	1	no	0	no	0	c, e
277	Michienzi, A. E., et al. (2015). "Lateral Epicondylitis and Tobacco Use: A Case-Control Study." Iowa Orthop J 35: 114-118.	PubMed_03_02_2017	no	0	no	0	0	0	0	0	exclusion; after scanning title & abstract (no; no)	1	no	0	no	0	c, e
278	Mischke C, Verbeek JH, Job J, Morata TC, Alvesalo-Kuusi A, Neuvonen K, Clarke S, Pedlow RI. Occupational safety and health enforcement tools for preventing occupational diseases and injuries. Cochrane Database of Systematic Reviews 2013, Issue 8. Art. No.: CD010183. DOI: 10.1002/14651858.CD010183.pub2.	CochraneWork_06_01_2017	yes	1	no	0	1	1	1	1	discussion necessary	4	no	0	no	0	c, e
279	Modi CS, Wasserstein D, Mayne IP, Henry PD, Mahomed N, Veillette CJ. The frequency and risk factors for subsequent surgery after a simple elbow dislocation. Injury. 2015;46(6):1156-60. doi: 10.1016/j.injury.2015.02.009. PubMed PMID: 25796345.	EMBASE_08_02_2017	no	0	no	0	0	0	0	0	exclusion; after scanning title & abstract (no; no)	1	no	0	no	0	b, c, e
280	Mohammadi, Ghorbanali. (2012). RISK FACTORS FOR THE PREVALENCE OF THE UPPER LIMB AND NECK WORK-RELATED MUSCULOSKELETAL DISORDERS AMONG POULTRY SLAUGHTER WORKERS. J. Musculoskelet. Res., 15, 1250005 (2012) [8 pages]	added by Seidel	yes	1	yes	1	3	2	2	1	inclusion for scanning full-text	5	no	0	no	0	e
281	Mohan, G. M., Prasad, P. S. S., & Sudarmathi, V. (2008). Simplified procedure to analyse work posture of foundry men through the rapid upper limb assessment. International Journal of Environment and Health, 2(2), 225-238.	added by Seidel	yes	1	yes	1	3	2	2	1	inclusion for scanning full-text	5	no	0	no	0	d
282	Mondam, S., Shaik, R., Prakash, J., Fook, J. L., & Nekkanti, S. (2016). Surveillance of Musculoskeletal Symptoms and Anthropometric Variables among Four International Cricket Teams Competed in ACC Premier League Malaysia 2014. Asian Journal of Pharmaceutical Research and Health Care, 8(2), 47-51.	EMBASE_08_02_2017	no	0	no	0	0	0	0	0	exclusion; after scanning title & abstract (no; no)	1	no	0	no	0	c, e
283	Mondelli, M., Aretini, A., Ciaramitaro, P. , Mattioli, S. 2014. Anthropometric, lifestyle and occupational risk factors for ulnar neuropathy at the elbow: Study design. onference: 4th Annual Meeting of the, Volume: 19	EMBASE_08_02_2017_Abstract_not found	no	0	no	0	0	0	0	0	exclusion; because abstract is missing	0	no	0	no	0	a
284	Moore, J. S. (2002). "Biomechanical models for the pathogenesis of specific distal upper extremity disorders." Am J Ind Med 41(5): 353-369.	added by Seidel	no	0	no	0	0	0	0	0	exclusion; after scanning title & abstract (no; no)	1	no	0	no	0	c, e
285	Mora, D. C., et al. (2016). "Prevalence of musculoskeletal disorders among immigrant Latino farmworkers and non-farmworkers in North Carolina." Arch Environ Occup Health 71(3): 136-143.	added by Seidel	yes	1	no	0	1	1	1	1	discussion necessary	4	no	0	no	0	c, e
286	Müezzinoğlu, Arif. "Role of Occupational Physician in Mining Accidents." Turkish Thoracic J 16.Suppl 1 (2015): 21-24.	EMBASE_08_02_2017_Abstract_not found	no	0	no	0	0	0	0	0	exclusion; because abstract is missing	0	no	0	no	0	a

287	Mullick, A. A., et al. (2013). "Stretch reflex spatial threshold measure discriminates between spasticity and rigidity." Clin Neurophysiol 124(4): 740-751.	PubMed_03_02_2017	no	0	no	0	0	0	0	0	exclusion; after scanning title & abstract (no; no)	1	no	0	no	0	b, d
288	Mulimani P, Hoe VCW, Hayes MJ, Idiculla JJ, Abas ABL, Karanth L. Ergonomic interventions for preventing musculoskeletal disorders in dental care practitioners. Cochrane Database of Systematic Reviews 2014, Issue 8. Art. No.: CD011261. DOI:10.1002/14651858.CD011261.	CochraneWork_06_01_2017	no	0	no	0	0	0	0	0	exclusion; after scanning title & abstract (no; no)	1	no	0	no	0	a
289	Murtaugh, Bryan, and Joseph M. Ihm. "Eccentric training for the treatment of tendinopathies." Current sports medicine reports 12.3 (2013): 175-182.	EMBASE_08_02_2017	no	0	no	0	0	0	0	0	exclusion; after scanning title & abstract (no; no)	1	no	0	no	0	c, e
290	Myers, J. B., et al. (2013). "Scapular dysfunction in high school baseball players sustaining throwing-related upper extremity injury: a prospective study." J Shoulder Elbow Surg 22(9): 1154-1159.	added by Seidel	no	0	no	0	0	0	0	0	exclusion; after scanning title & abstract (no; no)	1	no	0	no	0	b, d
291	Nandi, S., Maschke, S., Evans, P. J., & Lawton, J. N. (2009). The stiff elbow. Hand, 4(4), 368-379.	EMBASE_08_02_2017	no	0	no	0	0	0	0	0	exclusion; after scanning title & abstract (no; no)	1	no	0	no	0	c, e
292	Nainzadeh, N. K., et al. (2011). "Ulnar neuropathy at the elbow in computer keyboard operators." Work 39(2): 93-101.	PubMed_03_02_2017	no	0	no	0	0	0	0	0	exclusion; after scanning title & abstract (no; no)	1	no	0	no	0	c, e
293	Nilsson, P., et al. (2012). "Lateral epicondylalgia. A quantitative and qualitative analysis of interdisciplinary cooperation and treatment choice in the Swedish health care system." Scand J Caring Sci 26(1): 28-37.	EMBASE_08_02_2017	no	0	no	0	0	0	0	0	exclusion; after scanning title & abstract (no; no)	1	no	0	no	0	b, c, e
294	Nilsson, P., et al. (2012). "Lateral epicondylalgia: a structured programme better than corticosteroids and NSAID." Scand J Occup Ther 19(5): 404-410.	EMBASE_08_02_2017	no	0	no	0	0	0	0	0	exclusion; after scanning title & abstract (no; no)	1	no	0	no	0	c, e
295	Nimura, A., et al. (2014). "Joint capsule attachment to the extensor carpi radialis brevis origin: an anatomical study with possible implications regarding the etiology of lateral epicondylitis." J Hand Surg Am 39(2): 219-225.	PubMed_03_02_2017	no	0	no	0	0	0	0	0	exclusion; after scanning title & abstract (no; no)	1	no	0	no	0	b, c, e
296	Noonan TJ, Thigpen CA, Bailey LB, Wyland DJ, Kissenberth M, Hawkins RJ, Shanley E. Humeral Torsion as a Risk Factor for Shoulder and Elbow Injury in Professional Baseball Pitchers. Am J Sports Med. 2016 Sep;44(9):2214-9. doi: 10.1177/0363546516648438. PubMed PMID: 27281279.	EMBASE_08_02_2017_Double	no	0	no	0	0	0	0	0	exclusion; after scanning title & abstract (no; no)	1	no	0	no	0	b, c, e
297	Nourissat G, Ciais G, Coudane H. Arthroscopy and obesity. Orthop TraumatolSurg Res. 2015 Dec;101(8 Suppl):S351-2. doi: 10.1016/j.otsr.2015.09.001. Review. PubMed PMID: 26552647.	EMBASE_08_02_2017	no	0	no	0	0	0	0	0	exclusion; after scanning title & abstract (no; no)	1	no	0	no	0	b, c, e
298	Nordander, C., et al. (2009). "Risk of musculoskeletal disorders among females and males in repetitive/constrained work." Ergonomics 52(10): 1226-1239.	PubMed_03_02_2017/ EMBASE_08_02_2017	yes	1	yes	1	3	2	2	1	inclusion for scanning full-text	5	yes	1	yes	1	no
299	Nordander, C., et al. (2013). "Exposure-response relationships in work-related musculoskeletal disorders in elbows and hands - A synthesis of group-level data on exposure and response obtained using uniform methods of data collection." Appl Ergon 44(2): 241-253.	PubMed_03_02_2017/ EMBASE_08_02_2017	yes	1	yes	1	3	2	2	1	inclusion for scanning full-text	5	yes	1	yes	1	no
300	Occhionero, V., et al. (2014). "Upper limb musculoskeletal disorders in healthcare personnel." Ergonomics 57(8): 1166-1191.	added by Seidel	no	0	no	0	0	0	0	0	exclusion; after scanning title & abstract (no; no)	1	no	0	no	0	c, e

301	Odell, D., Barr, A., Goldberg, R., Chung, J., & Rempel, D. (2007). Evaluation of a dynamic arm support for seated and standing tasks: a laboratory study of electromyography and subjective feedback. Ergonomics, 50(4), 520-535.	added by Seidel	yes	1	no	0	1	1	1	1	discussion necessary	4	no	0	no	0	c, d, e
302	Oh, J. H., et al. (2011). "The prevalence of shoulder osteoarthritis in the elderly Korean population: association with risk factors and function." J Shoulder Elbow Surg 20(5): 756-763.	PubMed_03_02_2017	no	0	no	0	0	0	0	0	exclusion; after scanning title & abstract (no; no)	1	no	0	no	0	d
303	Oh, S., et al. (2013). "Causes of hand tingling in visual display terminal workers." Ann Rehabil Med 37(2): 221-228.	added by Seidel	yes	1	no	0	1	1	1	1	discussion necessary	4	no	0	no	0	c, e
304	Olaussen M, Holmedal O, Lindbaek M, Brage S, Solvang H. Treating lateral epicondylitis with corticosteroid injections or non-electrotherapeutical physiotherapy: a systematic review. BMJ Open. 2013 Oct 29;3(10):e003564. doi: 10.1136/bmjopen-2013-003564. PubMed PMID: 24171937; PubMed Central PMCID: PMC3816235.	EMBASE_08_02_2017	no	0	no	0	0	0	0	0	exclusion; after scanning title & abstract (no; no)	1	no	0	no	0	b, c, e
305	Oliveira Dantas FF, de Lima KC. The relationship between physical load and musculoskeletal complaints among Brazilian dentists. Appl Ergon. 2015 Mar;47:93-8. doi: 10.1016/j.apergo.2014.09.003. PubMed PMID: 25479978.	EMBASE_08_02_2017	yes	1	yes	1	3	2	2	1	inclusion for scanning full-text	5	no	0	no	0	d, e
306	Oliveira, G. R., et al. (2008). "Peripheral neuropathy and neurological disorders in an unselected Brazilian population-based cohort of IBD patients." Inflamm Bowel Dis 14(3): 389-395.	PubMed_03_02_2017	no	0	no	0	0	0	0	0	exclusion; after scanning title & abstract (no; no)	1	no	0	no	0	d
307	Oliveira, A. B., Silva, L. C., & Coury, H. J. (2011). How do low/high height and weight variation affect upper limb movements during manual material handling of industrial boxes?. Brazilian Journal of Physical Therapy, 15(6), 494-502.	added by Seidel	yes	1	no	0	1	1	1	1	discussion necessary	4	no	0	no	0	c, e
308	Omrane, A., Kammoun, S., Bouzgarrou, L., Rassas, I., Allagui, I., Kraiem, A., ... & Chaari, N. (2015). Musculoskeletal disorders of the upper limbs: A scourge among nursing staff. Annals of Physical and Rehabilitation Medicine, 58, e36.	EMBASE_08_02_2017	yes	1	no	0	1	1	1	1	discussion necessary	4	no	0	no	0	c, e
309	Omejec, G. and S. Podnar (2016). "What causes ulnar neuropathy at the elbow?" Clin Neurophysiol 127(1): 919-924.	PubMed_03_02_2017	yes	1	yes	1	3	2	2	1	inclusion for scanning full-text	5	no	0	no	0	e
310	Ophir, A., et al. (2014). "An uncommon pattern of polyneuropathy induced by lifetime exposures to drift containing organophosphate pesticides." Neurotoxicology 45: 338-346.	PubMed_03_02_2017	no	0	no	0	0	0	0	0	exclusion; after scanning title & abstract (no; no)	1	no	0	no	0	c, e
311	Osborne, A., Blake, C., Fullen, B. M., Meredith, D., Phelan, J., McNamara, J., & Cunningham, C. (2012). Prevalence of musculoskeletal disorders among farmers: a systematic review. American journal of industrial medicine, 55(2), 143-158.	added by Seidel	yes	1	no	0	1	1	1	1	discussion necessary	4	no	0	no	0	c, e
312	O'Shaughnessy, M. A., et al. (2016). "A Rare Diagnosis: Recognizing and Managing Fungal Tenosynovitis of the Hand and Upper Extremity." J Hand Surg Am.	added by Seidel	no	0	no	0	0	0	0	0	exclusion; after scanning title & abstract (no; no)	1	no	0	no	0	d
313	Oshlag BL, Ray TR. Elbow Injuries in the Young Throwing Athlete. Curr Sports Med Rep. 2016 Sep-Oct;15(5):325-9. doi: 10.1249/JSR.0000000000000300. PubMed PMID: 27618241.	EMBASE_08_02_2017	no	0	no	0	0	0	0	0	exclusion; after scanning title & abstract (no; no)	1	no	0	no	0	b
314	Otoshi, K., et al. (2015). "Chronic hyperglycemia increases the risk of lateral epicondylitis: the Locomotive Syndrome and Health Outcome in Aizu Cohort Study (LOHAS)." Springerplus 4: 407.	added by Seidel	no	0	no	0	0	0	0	0	exclusion; after scanning title & abstract (no; no)	1	no	0	no	0	c, e

315	Özdolap, S., et al. (2013). "Upper limb tendinitis and entrapment neuropathy in coal miners." Am J Ind Med 56(5): 569-575.	PubMed_03_02_2017/ EMBASE_08_02_2017	yes	1	no	0	1	1	1	1	discussion necessary	4	no	0	no	0	c, e
316	Padula, R. S., Comper, M. L. C., Sparer, E. H., & Dennerlein, J. T. (2017). Job rotation designed to prevent musculoskeletal disorders and control risk in manufacturing industries: A systematic review. Applied Ergonomics, 58, 386-397.	added by Seidel	yes	1	no	0	1	1	1	1	discussion necessary	4	no	0	no	0	c, e
317	Palmer, K. T. (2008). "Diagnosing soft tissue rheumatic disorders of the upper limb in epidemiological studies of vibration-exposed populations." Int Arch Occup Environ Health 81(5): 575-593.	EMBASE_08_02_2017	no	0	no	0	0	0	0	0	exclusion; after scanning title & abstract (no; no)	1	no	0	no	0	d, e
318	Palmer, K. T., et al. (2012). "Optimising case definitions of upper limb disorder for aetiological research and prevention: a review." Occup Environ Med 69(1): 71-78.	EMBASE_08_02_2017	yes	1	no	0	1	1	1	1	discussion necessary	4	no	0	no	0	c, e
319	Pandy, R. (2013). "Prevalence of upper limb disorders among female librarians." Occup Med (Lond) 63(6): 432-434.	PubMed_03_02_2017	yes	1	no	0	1	1	1	1	discussion necessary	4	no	0	no	0	c, e
320	Pansard, E., et al. (2013). "Reliability and validity assessment of a glenoid bone loss measurement using the Bernageau profile view in chronic anterior shoulder instability." J Shoulder Elbow Surg 22(9): 1193-1198.	PubMed_03_02_2017	no	0	no	0	0	0	0	0	exclusion; after scanning title & abstract (no; no)	1	no	0	no	0	d
321	Pensri, P., et al. (2009). "Prevalence of self-reported musculoskeletal symptoms in salespersons." Occup Med (Lond) 59(7): 499-501.	EMBASE_08_02_2017	no	0	no	0	0	0	0	0	exclusion; after scanning title & abstract (no; no)	1	no	0	no	0	c, e
322	Peters, J. A., Zwerver, J., Diercks, R. L., Elferink-Gemser, M. T., & van den Akker-Scheek, I. (2016). Preventive interventions for tendinopathy: A systematic review. Journal of Science and Medicine in Sport, 19(3), 205-211.	added by Seidel	no	0	no	0	0	0	0	0	exclusion; after scanning title & abstract (no; no)	1	no	0	no	0	c, e
323	Papa, J. A. (2012). "Two cases of work-related lateral epicondylopathy treated with Graston Technique(R) and conservative rehabilitation." J Can Chiropr Assoc 56(3): 192-200.	added by Seidel	no	0	no	0	0	0	0	0	exclusion; after scanning title & abstract (no; no)	1	no	0	no	0	c, e
324	Parida, R., & Ray, P. K. (2012). Study and analysis of occupational risk factors for ergonomic design of construction worksystems. Work, 41(Supplement 1), 3788-3794.	added by Seidel	yes	1	yes	1	3	2	2	1	inclusion for scanning full-text	5	no	0	no	0	e, d
325	Parihar JK, Jain VK, Chaturvedi P, Kaushik J, Jain G, Parihar AK. Computer and visual display terminals (VDT) vision syndrome (CVDTS). Med J Armed Forces India. 2016 Jul;72(3):270-6. doi: 10.1016/j.mjafi.2016.03.016. Review. PubMed PMID: 27546968; PubMed Central PMCID: PMC4982978.	EMBASE_08_02_2017	yes	1	yes	1	3	2	2	1	inclusion for scanning full-text	5	no	0	no	0	e
326	Park, J. H., et al. (2009). "Effects of vocalization on elbow motion during reaching in persons with hemiparetic stroke." NeuroRehabilitation 25(2): 123-128.	PubMed_03_02_2017	no	0	no	0	0	0	0	0	exclusion; after scanning title & abstract (no; no)	1	no	0	no	0	d
327	Park, J. K., & Jang, S. H. (2010). Association between upper extremity musculoskeletal disorders and psychosocial factors at work: a review on the Job DCS Model's perspective. Safety and health at work, 1(1), 37-42.	added by Seidel	no	0	no	0	0	0	0	0	exclusion; after scanning title & abstract (no; no)	1	no	0	no	0	c, e
328	Ratri Parida, Pradip Kumar Ray, Biomechanical Modelling of Manual Material Handling Tasks: A Comprehensive Review, Procedia Manufacturing, Volume 3, 2015, Pages 4598-4605, ISSN 2351-9789,	added by Seidel	yes	1	yes	1	3	2	2	1	inclusion for scanning full-text	5	no	0	no	0	e

	http://dx.doi.org/10.1016/j.promfg.2015.07.539. (http://www.sciencedirect.com/science/article/pii/S2351978915005405)																
329	PANEL, EXPERT. "Selected Issues in Injury and Illness Prevention andtheTeamPhysician: AConsensusStatement." Medicine & Science in Sports & Exercise 500 (2007): 2059.	EMBASE_08_02_2017_Abstract_not found	no	0	no	0	0	0	0	0	exclusion; because abstract is missing	0	no	0	no	0	a
330	Pasternak, B., Jr., et al. (2012). "Analysis of kinematic, kinetic and electromyographic patterns during root canal preparation with rotary and manual instruments." J Appl Oral Sci 20(1): 57-63.	PubMed_03_02_2017	yes	1	no	0	1	1	1	1	discussion necessary	4	no	0	no	0	d, e
331	Petit Le Manac'h A, Roquelaure Y, Ha C, Bodin J, Meyer G, Bigot F, Veaudor M, Descatha A, Goldberg M, Imbernon E. Risk factors for de Quervain's disease in a French working population. Scand J Work Environ Health. 2011 Sep;37(5):394-401. doi: 10.5271/sjweh.3160. PubMed PMID: 21431276.	EMBASE_08_02_2017	no	0	no	0	0	0	0	0	exclusion; after scanning title & abstract (no; no)	1	no	0	no	0	d
332	Pieber, K., et al. (2012). "Acute injuries and overuse syndromes in sport climbing and bouldering in Austria: a descriptive epidemiological study." Wien Klin Wochenschr 124(11-12): 357-362.	PubMed_03_02_2017/ EMBASE_08_02_2017	no	0	no	0	0	0	0	0	exclusion; after scanning title & abstract (no; no)	1	no	0	no	0	b
333	Pienimaki, T., et al. (2011). "Widespread pain in chronic epicondylitis." Eur J Pain 15(9): 921-927.	PubMed_03_02_2017/ EMBASE_08_02_2017	yes	1	no	0	1	1	1	1	discussion necessary	4	no	0	no	0	c, e
334	Pienimaki, T. T., et al. (2008). "Three-phase bone scintigraphy in chronic epicondylitis." Arch Phys Med Rehabil 89(11): 2180-2184.	PubMed_03_02_2017	no	0	no	0	0	0	0	0	exclusion; after scanning title & abstract (no; no)	1	no	0	no	0	c, e
335	Piper, S., et al. (2016). "The effectiveness of soft-tissue therapy for the management of musculoskeletal disorders and injuries of the upper and lower extremities: A systematic review by the Ontario Protocol for Traffic Injury management (OPTIMa) collaboration." Man Ther 21: 18-34.	EMBASE_08_02_2017	no	0	no	0	0	0	0	0	exclusion; after scanning title & abstract (no; no)	1	no	0	no	0	b, c, e
336	Ploumis A, Belbasis L, Ntzani E, Tsekeris P, Xenakis T. Radiotherapy for prevention of heterotopic ossification of the elbow: a systematic review of the literature. J Shoulder Elbow Surg. 2013 Nov;22(11):1580-8. doi: 10.1016/j.jse.2013.07.045. Review. PubMed PMID: 24138821.	EMBASE_08_02_2017	no	0	no	0	0	0	0	0	exclusion; after scanning title & abstract (no; no)	1	no	0	no	0	b, c, e
337	Prodromo J, Rackley J, Mulcahey MK. A review of important medical and surgicalconsiderations for obese patients undergoing arthroscopic surgery. PhysSportsmed. 2016 Sep;44(3):231-9. doi: 10.1080/00913847.2016.1221750. Review.PubMed PMID: 27578242.	EMBASE_08_02_2017	no	0	no	0	0	0	0	0	exclusion; after scanning title & abstract (no; no)	1	no	0	no	0	b, c, e
338	Polster, J. M., et al. (2016). "Throwing-related injuries of the subscapularis in professional baseball players." Skeletal Radiol 45(1): 41-47.	PubMed_03_02_2017	no	0	no	0	0	0	0	0	exclusion; after scanning title & abstract (no; no)	1	no	0	no	0	d
339	Poulsen, T. R., et al. (2014). "Health of Danish seafarers and fishermen 1970-2010: What have register-based studies found?" Scand J Public Health 42(6): 534-545.	PubMed_03_02_2017	no	0	no	0	0	0	0	0	exclusion; after scanning title & abstract (no; no)	1	no	0	no	0	c, e
340	Pullopdissakul, S., et al. (2013). "Upper extremities musculoskeletal disorders: prevalence and associated ergonomic factors in an electronic assembly factory." Int J Occup Med Environ Health 26(5): 751-761.	PubMed_03_02_2017	yes	1	yes	1	3	2	2	1	inclusion for scanning full-text	5	yes	1	no	0	e

341	Qin, J., et al. (2014). "Upper extremity kinematic and kinetic adaptations during a fatiguing repetitive task." J Electromyogr Kinesiol 24(3): 404-411.	PubMed_03_02_2017	yes	1	yes	1	3	2	2	1	inclusion for scanning full-text	5	no	0	no	0	d
342	Rafie, F., Zamani Jam, A., Shahravan, A., Raoof, M., & Eskandarizadeh, A. (2015). Prevalence of upper extremity musculoskeletal disorders in dentists: symptoms and risk factors. Journal of environmental and public health, 2015.	added by Seidel	no	0	yes	1	2	1	1	1	discussion necessary	4	no	0	no	0	d
343	Raghunathan, R., Maiti, J., & Samanta, B. (2014). Application of the Cube Model for Biomechanical Exposure Assessment of Combined Manual Material Handling Tasks in a Manufacturing Plant in India. IIE Transactions on Occupational Ergonomics and Human Factors, 2(1), 39-51.	added by Seidel	yes	1	no	0	1	1	1	1	discussion necessary	4	no	0	no	0	c, e
344	Rahman, M. N. A., & Razak, N. S. A. (2016). Review on Pen and Paper Based Observational Methods for Assessing Work-related Upper Limb Disorders. Indian Journal of Science and Technology, 9(S1).	added by Seidel	no	0	no	0	0	0	0	0	exclusion; after scanning title & abstract (no; no)	1	no	0	no	0	b, c, d, e
345	Rajabally, Y. A. and M. Narasimhan (2011). "Electrophysiological entrapment syndromes in chronic inflammatory demyelinating polyneuropathy." Muscle Nerve 44(3): 444-447.	PubMed_03_02_2017	no	0	no	0	0	0	0	0	exclusion; after scanning title & abstract (no; no)	1	no	0	no	0	b, c, e
346	Rajen N. Naidoo, Syed Atiqul Haq, Occupational use syndromes, Best Practice & Research Clinical Rheumatology, Volume 22, Issue 4, August 2008, Pages 677-691, ISSN 1521-6942, http://dx.doi.org/10.1016/j.berh.2008.04.001.	EMBASE_08_02_2017	yes	1	no	0	1	1	1	1	discussion necessary	4	no	0	no	0	c, e
347	Ramponi, D. R. and J. A. Kaufmann (2012). "Elbow injuries and fractures." Adv Emerg Nurs J 34(2): 99-109; quiz 110-101.	added by Seidel	no	0	no	0	0	0	0	0	exclusion; after scanning title & abstract (no; no)	1	no	0	no	0	b, c, d, e
348	Ratzlaff, C. R., Gillies, J. H., & Koehoorn, M. W. (2007). Work-related repetitive strain injury and leisure-time physical activity. Arthritis Care & Research, 57(3), 495-500.	added by Seidel	no	0	yes	1	2	1	1	1	discussion necessary	4	no	0	no	0	e
349	Rautiainen R, Lehtola MM, Day LM, Schonstein E, Suutarinen J, Salminen S, Verbeek JH. Interventions for preventing injuries in the agricultural industry. Cochrane Database of Systematic Reviews 2008, Issue 1. Art. No.: CD006398. DOI: 10.1002/14651858.CD006398.pub2.	CochraneWork_06_01_2017	no	0	no	0	0	0	0	0	exclusion; after scanning title & abstract (no; no)	1	no	0	no	0	c, e
350	Pradip Kumar Ray, Ratri Parida, Sagar Sarkar, Ergonomic Analysis of Construction Jobs in India: A Biomechanical Modelling Approach, Procedia Manufacturing, Volume 3, 2015, Pages 4606-4612, ISSN 2351-9789, http://dx.doi.org/10.1016/j.promfg.2015.07.542. (http://www.sciencedirect.com/science/article/pii/S2351978915005430)	added by Seidel	no	0	no	0	0	0	0	0	exclusion; after scanning title & abstract (no; no)	1	no	0	no	0	d
351	Rechardt M, Shiri R, Lindholm H, et al. Associations of metabolic factors and adipokines with pain in incipient upper extremity soft tissue disorders: a cross-sectional study.BMJOpen2013;3:e003036. doi:10.1136/bmjopen-2013-003036	EMBASE_08_02_2017	no	0	no	0	0	0	0	0	exclusion; after scanning title & abstract (no; no)	1	no	0	no	0	c, e
352	Rechardt, M., et al. (2011). "Soluble IL-1RII and IL-18 are associated with incipient upper extremity soft tissue disorders." Cytokine 54(2): 149-153.	EMBASE_08_02_2017	no	0	no	0	0	0	0	0	exclusion; after scanning title & abstract (no; no)	1	no	0	no	0	b, c, e
353	Rechardt, M., et al. (2014). "Adipokines as predictors of recovery from upper extremity soft tissue disorders." Rheumatology (Oxford) 53(12): 2238-2242.	PubMed_03_02_2017	no	0	no	0	0	0	0	0	exclusion; after scanning title & abstract (no; no)	1	no	0	no	0	b, c, e

354	Rhyou IH, Lim KS, Kim KC, Lee JH, Ahn KB, Moon SC. Drop sign of the elbow joint after surgical stabilization of an unstable simple posterolateral dislocation: natural course and contributing factors. J Shoulder Elbow Surg. 2015 Jul;24(7):1081-9. doi: 10.1016/j.jse.2015.01.018. PubMed PMID: 25825136.	EMBASE_08_02_2017	no	0	no	0	0	0	0	0	exclusion; after scanning title & abstract (no; no)	1	no	0	no	0	b, c, e
355	Ring, D. (2008). "Displaced, unstable fractures of the radial head: fixation vs. replacement--what is the evidence?" Injury 39(12): 1329-1337.	EMBASE_08_02_2017	no	0	no	0	0	0	0	0	exclusion; after scanning title & abstract (no; no)	1	no	0	no	0	b, d
356	Riviere, S., et al. (2014). "Underreporting of musculoskeletal disorders in 10 regions in France in 2009." Am J Ind Med 57(10): 1174-1180.	PubMed_03_02_2017	yes	1	no	0	1	1	1	1	discussion necessary	4	no	0	no	0	c, e
357	Roquelaure, Y., et al. (2009). "Risk factors for upper-extremity musculoskeletal disorders in the working population." Arthritis Rheum 61(10): 1425-1434.	EMBASE_08_02_2017	yes	1	yes	1	3	2	2	1	inclusion for scanning full-text	5	no	0	no	0	e
358	Roquelaure, Y., LeManach, A. P., Ha, C., Poisnel, C., Bodin, J., Descatha, A., & Imbernon, E. (2012). Working in temporary employment and exposure to musculoskeletal constraints. Occupational medicine, kqs004.	added by Seidel	yes	1	yes	1	3	2	2	1	inclusion for scanning full-text	5	yes	1	no	0	e
359	Roscher, Patrick (2012).Three-Dimensional Biomechanical Model of Wrist Dynamics during Tasks of Daily LivingMarquette University, Biomedical Engineering	added by Seidel	no	0	no	0	0	0	0	0	exclusion; after scanning title & abstract (no; no)	1	no	0	no	0	b, c, d, e
360	Rose, N. E., et al. (2013). "Denervation of the lateral humeral epicondyle for treatment of chronic lateral epicondylitis." J Hand Surg Am 38(2): 344-349.	EMBASE_08_02_2017	no	0	no	0	0	0	0	0	exclusion; after scanning title & abstract (no; no)	1	no	0	no	0	b, d, e
361	Rosenbaum, D. A., et al. (2013). "Prevalence of epicondylitis, rotator cuff syndrome, and low back pain in Latino poultry workers and manual laborers." Am J Ind Med 56(2): 226-234.	PubMed_03_02_2017/ EMBASE_08_02_2017	no	0	no	0	0	0	0	0	exclusion; after scanning title & abstract (no; no)	1	no	0	no	0	c, e
362	Rosenbaum, D. A., et al. (2014). "Employer differences in upper-body musculoskeletal disorders and pain among immigrant Latino poultry processing workers." J Agromedicine 19(4): 384-394.	PubMed_03_02_2017/ EMBASE_08_02_2017	yes	1	yes	1	3	2	2	1	inclusion for scanning full-text	5	no	0	no	0	e
363	Rosenzweig, Lee E., and Joshua S. Dines. "Conservative and Surgical Management of Tennis Elbow." Techniques in Shoulder & Elbow Surgery 15.1 (2014): 28-35.	EMBASE_08_02_2017	no	0	no	0	0	0	0	0	exclusion; after scanning title & abstract (no; no)	1	no	0	no	0	c, e
364	Ross, M. W., et al. (2012). "Occupational health and safety among commercial sex workers." Scand J Work Environ Health 38(2): 105-119.	EMBASE_08_02_2017	no	0	no	0	0	0	0	0	exclusion; after scanning title & abstract (no; no)	1	no	0	no	0	c, e
365	Rossi, J., et al. (2014). "Potential effects of racket grip size on lateral epicondilalgy risks." Scand J Med Sci Sports 24(6): e462-470.	PubMed_03_02_2017/ EMBASE_08_02_2017	no	0	yes	1	2	1	1	1	discussion necessary	4	no	0	no	0	d
366	Sadeghian, F., Sadeghian, A., Raei, M., & Kasaeian, A. (2012). Musculoskeletal disorders among oil field workers: influences of health beliefs, mental health and somatisation tendency. Journal of Medical Sciences, 12(4), 114.	EMBASE_08_02_2017	no	0	no	0	0	0	0	0	exclusion; after scanning title & abstract (no; no)	1	no	0	no	0	c, e

367	Salazar D, Golz A, Israel H, Marra G. Heterotopic ossification of the elbow treated with surgical resection: risk factors, bony ankylosis, and complications. Clin Orthop Relat Res. 2014 Jul;472(7):2269-75. doi: 10.1007/s11999-014-3591-0.PubMed PMID: 24711127; PubMed Central PMCID: PMC4048438.	EMBASE_08_02_2017	no	0	no	0	0	0	0	0	exclusion; after scanning title & abstract (no; no)	1	no	0	no	0	b, c, e
368	Saltzman BM, Chalmers PN, Mascarenhas R, Cole BJ, Romeo AA. Upper extremity physeal injury in young baseball pitchers. Phys Sportsmed. 2014 Sep;42(3):100-11. doi: 10.3810/psm.2014.09.2081. Review. PubMed PMID: 25295772.	EMBASE_08_02_2017	no	0	no	0	0	0	0	0	exclusion; after scanning title & abstract (no; no)	1	no	0	no	0	b, d, e
369	Saito, M., et al. (2014). "Relationship Between Tightness of the Hip Joint and Elbow Pain in Adolescent Baseball Players." Orthop J Sports Med 2(5): 2325967114532424.	added by Seidel	no	0	no	0	0	0	0	0	exclusion; after scanning title & abstract (no; no)	1	no	0	no	0	b, d, e
370	Salles, J. I., et al. (2015). "BMP4 and FGF3 haplotypes increase the risk of tendinopathy in volleyball athletes." J Sci Med Sport 18(2): 150-155.	added by Seidel	no	0	no	0	0	0	0	0	exclusion; after scanning title & abstract (no; no)	1	no	0	no	0	b, d, e
371	Sandell, C., Frykman, M., Chesky, K., & Fjellman-Wiklund, A. (2009). Playing-related musculoskeletal disorders and stress-related health problems among percussionists. Med Probl Perform Art, 24(4), 175-180.	EMBASE_08_02_2017	no	0	no	0	0	0	0	0	exclusion; after scanning title & abstract (no; no)	1	no	0	no	0	b, c, e
372	Sandoughi M, Zakeri Z, Tehrani Banihashemi A, Davatchi F, Narouie B, Shikhzadeh A, Mohammadi M, Jahantigh M, Shakiba M, Moulaei NA, Yousefi S, Rezazehi B, Shirzaei Sani E, Emamdadi A, Hoseynian M, Masoodian S, Shahbakhsh S. Prevalence of musculoskeletal disorders in southeastern Iran: a WHO-ILAR COPCORD study (stage 1, urban study). Int J Rheum Dis. 2013 Oct;16(5):509-17. doi: 10.1111/1756-185X.12110. PubMed PMID: 24164837.	EMBASE_08_02_2017	no	0	no	0	0	0	0	0	exclusion; after scanning title & abstract (no; no)	1	no	0	no	0	b, d
373	Santos, J., Baptista, J. S., Monteiro, P. R. R., Miguel, A. S., Santos, R., & Vaz, M. A. (2016). The influence of task design on upper limb muscles fatigue during low-load repetitive work: A systematic review. International Journal of Industrial Ergonomics, 52, 78-91.	added by Seidel	yes	1	no	0	1	1	1	1	discussion necessary	4	no	0	no	0	c, e
374	Sato, J., et al. (2016). "Clinical and ultrasound features in patients with intersection syndrome or de Quervain's disease." J Hand Surg Eur Vol 41(2): 220-225.	PubMed_03_02_2017	no	0	no	0	0	0	0	0	exclusion; after scanning title & abstract (no; no)	1	no	0	no	0	d
375	Saupe, N., et al. (2009). "Pain and other side effects after MR arthrography: prospective evaluation in 1085 patients." Radiology 250(3): 830-838.	PubMed_03_02_2017	no	0	no	0	0	0	0	0	exclusion; after scanning title & abstract (no; no)	1	no	0	no	0	b, c, d, e
376	Sauni R, Virtema P, Paakkonen R, et al. Quality of life (EQ-5D) and hand-arm vibration syndrome. International archives of occupational and environmental health 2010;83(2):209-16. doi: 10.1007/s00420-009-0441-6 [published Online First: 2009/07/14]	PubMed_03_02_2017	no	0	no	0	0	0	0	0	exclusion; after scanning title & abstract (no; no)	1	no	0	no	0	d
377	Schaafsma FG, Mahmud N, Reneman MF, Fassier JB, Jungbauer FHW . Pre-employment examinations for preventing injury, disease and sick leave in workers. Cochrane Database of Systematic Reviews 2016, Issue 1. Art. No.: CD008881. DOI:10.1002/14651858.CD008881.pub2.	CochraneWork_06_01_2017	no	0	no	0	0	0	0	0	exclusion; after scanning title & abstract (no; no)	1	no	0	no	0	c, e
378	Schaefer, P. T. and J. Speier (2012). "Common medical problems of instrumental athletes." Curr Sports Med Rep 11(6): 316-322.	EMBASE_08_02_2017	no	0	no	0	0	0	0	0	exclusion; after scanning title & abstract (no; no)	1	no	0	no	0	c, e

379	Schlechter JA. Reducing Cumulative Arm Overuse Injuries in Young Throwers. Pediatr Ann. 2016 Jan;45(1):e15-20. doi: 10.3928/00904481-20151209-01. PubMed PMID: 26783969.	EMBASE_08_02_2017	no	0	no	0	0	0	0	0	exclusion; after scanning title & abstract (no; no)	1	no	0	no	0	b
380	Schnorenberg, A. J., et al. (2014). "Biomechanical model for evaluation of pediatric upper extremity joint dynamics during wheelchair mobility." J Biomech 47(1): 269-276.	PubMed_03_02_2017	no	0	no	0	0	0	0	0	exclusion; after scanning title & abstract (no; no)	1	no	0	no	0	b
381	Schupp, C. M. and A. Bedgood (2013). "Sideline management from head to toe of the skeletally immature athlete." Curr Sports Med Rep 12(3): 162-169.	EMBASE_08_02_2017	no	0	no	0	0	0	0	0	exclusion; after scanning title & abstract (no; no)	1	no	0	no	0	b, c, e
382	Sciascia, A., et al. (2015). "Preseason Perceived Physical Capability and Previous Injury." J Athl Train 50(9): 937-943.	PubMed_03_02_2017	no	0	no	0	0	0	0	0	exclusion; after scanning title & abstract (no; no)	1	no	0	no	0	b, c, e
383	SEILS, DREW R., "A Comprehensive Methodology for Assessing Biomechanical Risks Associated with Hand Tool Use: Applied to Laparoscopic Surgical Instruments" (2012). Master's Theses. 282. http://digitalcommons.uconn.edu/gs_theses/282	added by Seidel	yes	1	yes	1	3	2	2	1	inclusion for scanning full-text	5	no	0	no	0	a
384	Seo, N. J., Armstrong, T. J., & Young, J. G. (2010). Effects of handle orientation, gloves, handle friction and elbow posture on maximum horizontal pull and push forces. Ergonomics, 53(1), 92-101.	added by Seidel	yes	1	yes	1	3	2	2	1	inclusion for scanning full-text	5	no	0	no	0	d
385	Serazin, C., et al. (2013). "Employment and occupational outcomes of workers with musculoskeletal pain in a French region." Occup Environ Med 70(3): 143-148.	PubMed_03_02_2017/ EMBASE_08_02_2017	yes	1	no	0	1	1	1	1	discussion necessary	4	no	0	no	0	c, e
386	Sethi, A., et al. (2013). "Temporal structure of variability decreases in upper extremity movements post stroke." Clin Biomech (Bristol, Avon) 28(2): 134-139.	PubMed_03_02_2017	no	0	no	0	0	0	0	0	exclusion; after scanning title & abstract (no; no)	1	no	0	no	0	b, c, e
387	Shanley, Ellen, et al. "Influence of a Prevention Program on Arm Injury Risk: An RCT in Adolescent Pitchers." Orthopaedic Journal of Sports Medicine 2.2 suppl (2014): 2325967114S00089.	EMBASE_08_02_2017_Abstract_not found	no	0	no	0	0	0	0	0	exclusion; because abstract is missing	0	no	0	no	0	a
388	Shin, G., & Zhu, X. (2011). User discomfort, work posture and muscle activity while using a touchscreen in a desktop PC setting. Ergonomics, 54(8), 733-744.	added by Seidel	yes	1	yes	1	3	2	2	1	inclusion for scanning full-text	5	no	0	no	0	d
389	Shiri, R. et al. (2007). Hand Dominance in Upper Extremity Musculoskeletal Disorders The Journal of Rheumatology 2007; 34:5	added by Seidel	yes	1	no	0	1	1	1	1	discussion necessary	4	no	0	no	0	b, c, e
390	Shiri R, Martimo KP, Miranda H, Ketola R, Kaila-Kangas L, Liira H, Karppinen J, Viikari-Juntura E. The effect of workplace intervention on pain and sickness absence caused by upper-extremity musculoskeletal disorders. Scand J Work Environ Health. 2011 Mar;37(2):120-8. doi: 10.5271/sjweh.3141. PubMed PMID: 21218270.	EMBASE_08_02_2017	no	0	no	0	0	0	0	0	exclusion; after scanning title & abstract (no; no)	1	no	0	no	0	c, e
391	Shiri, R. and E. Viikari-Juntura (2011). "Lateral and medial epicondylitis: role of occupational factors." Best Pract Res Clin Rheumatol 25(1): 43-57.	EMBASE_08_02_2017	yes	1	yes	1	3	2	2	1	inclusion for scanning full-text	5	yes	1	no	0	review, no single study
392	Shiri, R. (2016). "Arthritis as a risk factor for carpal tunnel syndrome: a meta-analysis." Scand J Rheumatol 45(5): 339-346.	added by Seidel	no	0	no	0	0	0	0	0	exclusion; after scanning title & abstract (no; no)	1	no	0	no	0	b, c, e

393	Shechtman, O., et al. (2007). "Evaluation of the torque-velocity test of the BTE-Primus as a measure of sincerity of effort of grip strength." J Hand Ther 20(4): 326-334; quiz 335.	PubMed_03_02_2017	no	0	no	0	0	0	0	0	exclusion; after scanning title & abstract (no; no)	1	no	0	no	0	b, c, e
394	Silvetti, A., et al. (2015). "Kinematic and electromyographic assessment of manual handling on a supermarket green- grocery shelf." Work 51(2): 261-271.	PubMed_03_02_2017	yes	1	yes	1	3	2	2	1	inclusion for scanning full-text	5	no	0	no	0	e
395	Sinclair, A. J. and C. Smidt (2009). "Analysis of 10 years of injury in high school rodeo." Clin J Sport Med 19(5): 383-387.	EMBASE_08_02_2017_Abstract_not found	no	0	no	0	0	0	0	0	exclusion; because abstract is missing	0	no	0	no	0	a
396	Simkins, M., et al. (2014). "Stroke-induced synergistic phase shifting and its possible implications for recovery mechanisms." Exp Brain Res 232(11): 3489-3499.	PubMed_03_02_2017	no	0	no	0	0	0	0	0	exclusion; after scanning title & abstract (no; no)	1	no	0	no	0	b, d
397	Smith-Forbes, E. V., et al. (2016). "Specificity of the minimal clinically important difference of the quick Disabilities of the Arm Shoulder and Hand (QDASH) for distal upper extremity conditions." J Hand Ther 29(1): 81-88; quiz 88.	PubMed_03_02_2017	no	0	no	0	0	0	0	0	exclusion; after scanning title & abstract (no; no)	1	no	0	no	0	b, c, e
398	Sole, J. S., et al. (2015). "Sonographic evaluation of the extensor carpi ulnaris in asymptomatic tennis players." Pm r 7(3): 255-263.	PubMed_03_02_2017	no	0	no	0	0	0	0	0	exclusion; after scanning title & abstract (no; no)	1	no	0	no	0	b, c, e
399	Solidaki, E., et al. (2010). "Work-related and psychological determinants of multisite musculoskeletal pain." Scand J Work Environ Health 36(1): 54-61.	EMBASE_08_02_2017	yes	1	yes	1	3	2	2	1	inclusion for scanning full-text	5	no	0	no	0	e
400	Soma DB. Opening the Black Box: Evaluating the Pediatric Athlete With Elbow Pain. PM R. 2016 Mar;8(3 Suppl):S101-12. doi: 10.1016/j.pmrj.2016.01.002. Review. PubMed PMID: 26972259.	EMBASE_08_02_2017	no	0	no	0	0	0	0	0	exclusion; after scanning title & abstract (no; no)	1	no	0	no	0	b
401	Sood, D., Nussbaum, M. A., & Hager, K. (2007). Fatigue during prolonged intermittent overhead work: reliability of measures and effects of working height. Ergonomics, 50(4), 497-513.	added by Seidel	no	0	no	0	0	0	0	0	exclusion; after scanning title & abstract (no; no)	1	no	0	no	0	c, e
402	Soong, M., et al. (2011). "Fracture of the distal radius: risk factors for complications after locked volar plate fixation." J Hand Surg Am 36(1): 3-9.	EMBASE_08_02_2017	no	0	no	0	0	0	0	0	exclusion; after scanning title & abstract (no; no)	1	no	0	no	0	b, c, e
403	Spahn G, Lipfert J-U, Schmidt A, et al. Fall-Kontrolle-Studie zur Bestimmung von Risikofaktoren der lateralen Epikondylitis. Arbeitsmed Sozialmed Umweltmed 2016;51:360–68	added by Seidel	yes	1	yes	1	3	2	2	1	inclusion for scanning full-text	5	yes	1	yes	1	no
404	Spyropoulos, E., Chroni, E., Katsakiori, P., & Athanassiou, G. (2013). A quantitative approach to assess upper limb fatigue in the work field. Occupational Ergonomics, 11(1), 45-57.	added by Seidel	no	0	no	0	0	0	0	0	exclusion; after scanning title & abstract (no; no)	1	no	0	no	0	c, e
405	Srinivasan, D., Rudolfsson, T., & Mathiassen, S. E. (2015). Between-and within-subject variance of motor variability metrics in females performing repetitive upper-extremity precision work. Journal of Electromyography and Kinesiology, 25(1), 121-129.	added by Seidel	no	0	no	0	0	0	0	0	exclusion; after scanning title & abstract (no; no)	1	no	0	no	0	c, e
406	Srinivasan, D., et al. (2015). "The combined influence of task accuracy and pace on motor variability in a standardised repetitive precision task." Ergonomics 58(8): 1388-1397.	PubMed_03_02_2017	yes	1	yes	1	3	2	2	1	inclusion for scanning full-text	5	no	0	no	0	d
407	Srinivasan, D., et al. (2015). "Effects of concurrent physical and cognitive demands on arm movement kinematics in a repetitive upper-extremity precision task." Hum Mov Sci 42: 89-99.	PubMed_03_02_2017	no	0	no	0	0	0	0	0	exclusion; after scanning title & abstract (no; no)	1	no	0	no	0	c, e

408	Srinivasan, D., Samani, A., Mathiassen, S. E., & Madeleine, P. (2015). The size and structure of arm movement variability decreased with work pace in a standardised repetitive precision task. Ergonomics, 58(1), 128-139.	added by Seidel	yes	1	yes	1	3	2	2	1	inclusion for scanning full-text	5	no	0	no	0	d
409	Steinhilber, B., Seibt, R., Reiff, F. et al. Effect of a laparoscopic instrument with rotatable handle piece on biomechanical stress during laparoscopic procedures. Surg Endosc (2016) 30: 78. doi:10.1007/s00464-015-4164-3	added by Seidel	yes	1	no	0	1	1	1	1	discussion necessary	4	no	0	no	0	d, e
410	Steven L. Fischer, Kathryn Woodcock, A cross-sectional survey of reported musculoskeletal pain, disorders, work volume and employment situation among sign language interpreters, International Journal of Industrial Ergonomics, Volume 42, Issue 4, July 2012, Pages 335-340, ISSN 0169-8141, http://dx.doi.org/10.1016/j.ergon.2012.03.003.	EMBASE_08_02_2017	yes	1	yes	1	3	2	2	1	inclusion for scanning full-text	5	no	0	no	0	e
411	Stephens, B. and D. P. Gross (2007). "The influence of a continuum of care model on the rehabilitation of compensation claimants with soft tissue disorders." Spine (Phila Pa 1976) 32(25): 2898-2904.	EMBASE_08_02_2017	no	0	no	0	0	0	0	0	exclusion; after scanning title & abstract (no; no)	1	no	0	no	0	c, e
412	Sun Y, Cai J, Li F, Liu S, Ruan H, Fan C. The efficacy of celecoxib in preventing heterotopic ossification recurrence after open arthrolysis for post-traumatic elbow stiffness in adults. J Shoulder Elbow Surg. 2015 Nov;24(11):1735-40. doi: 10.1016/j.jse.2015.07.006. PubMed PMID: 26480878.	EMBASE_08_02_2017	no	0	no	0	0	0	0	0	exclusion; after scanning title & abstract (no; no)	1	no	0	no	0	b, c, e
413	Sutton, D., et al. (2016). "Multimodal care for the management of musculoskeletal disorders of the elbow, forearm, wrist and hand: a systematic review by the Ontario Protocol for Traffic Injury Management (OPTIMa) Collaboration." Chiropr Man Therap 24: 8.	added by Seidel	no	0	no	0	0	0	0	0	exclusion; after scanning title & abstract (no; no)	1	no	0	no	0	c, e
414	Suzuki, H., et al. (2013). "Effects of a vertical console position on operator muscular stress during ultrasonic diagnosis." J Med Ultrason (2001) 40(3): 189-195.	added by Seidel	yes	1	yes	1	3	2	2	1	inclusion for scanning full-text	5	no	0	no	0	e
415	Svendsen SW, Johnsen B, Fuglsang-Frederiksen A, et al Ulnar neuropathy and ulnar neuropathy-like symptoms in relation to biomechanical exposures assessed by a job exposure matrix: a triple case–referent study Occup Environ Med 2012;69:773-780.	added by Seidel	yes	1	yes	1	3	2	2	1	inclusion for scanning full-text	5	yes	1	yes	1	no
416	Tajika, T., et al. (2014). "Prevalence and risk factors of lateral epicondylitis in a mountain village in Japan." J Orthop Surg (Hong Kong) 22(2): 240-243.	PubMed_03_02_2017/ EMBASE_08_02_2017	no	0	yes	1	2	1	1	1	discussion necessary	4	no	0	no	0	e
417	Talbot CL, Ring J, Holt EM. Litigation relating to conditions affecting the shoulder and elbow: an analysis of claims against the National Health Service. Bone Joint J. 2014 May;96-B(5):574-9. doi: 10.1302/0301-620X.96B5.33257. PubMedPMID: 24788489.	EMBASE_08_02_2017	no	0	no	0	0	0	0	0	exclusion; after scanning title & abstract (no; no)	1	no	0	no	0	c, e
418	Tanaka, H., et al. (2007). "The mode of destruction in shoulders with rheumatoid arthritis based on radiographic findings." J Shoulder Elbow Surg 16(5): 539-543.	PubMed_03_02_2017	no	0	no	0	0	0	0	0	exclusion; after scanning title & abstract (no; no)	1	no	0	no	0	b, d
419	Taneja, A. K., et al. (2013). "Peroneal tendon abnormalities in subjects with an enlarged peroneal tubercle." Skeletal Radiol 42(12): 1703-1709.	PubMed_03_02_2017	no	0	no	0	0	0	0	0	exclusion; after scanning title & abstract (no; no)	1	no	0	no	0	b, d
420	Tang, K., et al. (2009). "Comparison of the psychometric properties of four at-work disability measures in workers with shoulder or elbow disorders." J Occup Rehabil 19(2): 142-154.	PubMed_03_02_2017/	no	0	no	0	0	0	0	0	exclusion; after scanning title & abstract (no; no)	1	no	0	no	0	c, d, e

		EMBASE_0 8_02_2017															
421	Tashjian, R. Z., et al. (2016). "Evidence for an Environmental and Inherited Predisposition Contributing to the Risk for Global Tendinopathies or Compression Neuropathies in Patients With Rotator Cuff Tears." Orthop J Sports Med 4(4): 2325967116642173.	EMBASE_0 8_02_2017	no	0	no	0	0	0	0	0	exclusion; after scanning title & abstract (no; no)	1	no	0	no	0	c, e
422	Taspinar, O., et al. (2014). "Upper extremity problems in doner kebab masters." J Phys Ther Sci 26(9): 1433-1436.	EMBASE_0 8_02_2017	yes	1	no	0	1	1	1	1	discussion necessary	4	no	0	no	0	c, e
423	Tat, J., et al. (2016). "Relative displacement of the tendon and subsynovial connective tissue using ultrasound captures different phenomena than mechanical tendon shear." J Biomech 49(15): 3682-3687.	added by Seidel	no	0	no	0	0	0	0	0	exclusion; after scanning title & abstract (no; no)	1	no	0	no	0	c, e
424	Thiese, M. S., et al. (2016). "Psychosocial Factors Related to Lateral and Medial Epicondylitis: Results From Pooled Study Analyses." J Occup Environ Med 58(6): 588-593.	EMBASE_0 8_02_2017	yes	1	no	0	1	1	1	1	discussion necessary	4	no	0	no	0	c, e
425	Thomas, S. J., et al. (2007). "Prevalence of symptoms and signs of shoulder problems in people with diabetes mellitus." J Shoulder Elbow Surg 16(6): 748-751.	PubMed_03 _02_2017	no	0	no	0	0	0	0	0	exclusion; after scanning title & abstract (no; no)	1	no	0	no	0	b, d
426	Tiric-Campara, M., et al. (2014). "Occupational overuse syndrome (technological diseases): carpal tunnel syndrome, a mouse shoulder, cervical pain syndrome." Acta Inform Med 22(5): 333-340.	added by Seidel	yes	1	yes	1	3	2	2	1	inclusion for scanning full-text	5	no	0	no	0	e
427	Titchener, A. G., et al. (2013). "Risk factors in lateral epicondylitis (tennis elbow): a case-control study." J Hand Surg Eur Vol 38(2): 159-164.	PubMed_03 _02_2017/E MBASE_08 _02_2017	no	0	yes	1	2	1	1	1	discussion necessary	4	no	0	no	0	e, b
428	Titchener AG, White JJ, Hinchliffe SR, Tambe AA, Hubbard RB, Clark DI. Comorbidities in rotator cuff disease: a case-control study. J Shoulder Elbow Surg. 2014 Sep;23(9):1282-8. doi: 10.1016/j.jse.2013.12.019. PubMed PMID: 24618192.	EMBASE_0 8_02_2017	no	0	no	0	0	0	0	0	exclusion; after scanning title & abstract (no; no)	1	no	0	no	0	c, d, e
429	Tjoumakaris, F. P., et al. (2012). "Eminence-based medicine versus evidence-based medicine: it's okay for 12-year-old pitchers to throw curveballs; it's the pitch count that matters." Phys Sportsmed 40(3): 83-86.	EMBASE_0 8_02_2017	no	0	no	0	0	0	0	0	exclusion; after scanning title & abstract (no; no)	1	no	0	no	0	b
430	Toosi, K. K., Impink, B. G., Baker, N. A., & Boninger, M. L. (2011). Effects of computer keyboarding on ultrasonographic measures of the median nerve. American journal of industrial medicine, 54(11), 826-833.	added by Seidel	no	0	no	0	0	0	0	0	exclusion; after scanning title & abstract (no; no)	1	no	0	no	0	c, d, e
431	Torabi, M., Martell, B., Tuohy, C. et al. Curr Radiol Rep (2016) 4: 10. doi:10.1007/s40134-015-0137-5	EMBASE_0 8_02_2017	no	0	no	0	0	0	0	0	exclusion; after scanning title & abstract (no; no)	1	no	0	no	0	c, e
432	Tunc, A. and B. D. Gungen (2016). "Carpal tunnel syndrome: Investigating the sensitivity of initial-diagnosis with electro-diagnostic tests in 600 cases and associated risk factors especially manual milking." J Back Musculoskelet Rehabil.	added by Seidel	no	0	no	0	0	0	0	0	exclusion; after scanning title & abstract (no; no)	1	no	0	no	0	d, e
433	Tsikopoulos K, Tsikopoulos A, Natsis K. Autologous whole blood or corticosteroid injections for the treatment of epicondylopathy and plantar fasciopathy? A systematic review and meta-analysis of	EMBASE_0 8_02_2017	no	0	no	0	0	0	0	0	exclusion; after scanning title & abstract (no; no)	1	no	0	no	0	c, e

	randomized controlled trials. Phys Ther Sport. 2016 Nov;22:114-122. doi: 10.1016/j.ptsp.2016.02.002.Review. PubMed PMID: 27085490.																
434	Ustuner, E., et al. (2013). "Sonographic examination of the common extensor tendon of the forearm at three different locations in the normal asymptomatic population." Surg Radiol Anat 35(7): 547-552.	PubMed_03_02_2017	no	0	no	0	0	0	0	0	exclusion; after scanning title & abstract (no; no)	1	no	0	no	0	c, d, e
435	Uzunkulaoglu, A., et al. (2016). "Association Between Gender, Body Mass Index, and Ulnar Nerve Entrapment at the Elbow: A Retrospective Study." J Clin Neurophysiol 33(6): 545-548.	added by Seidel	no	0	no	0	0	0	0	0	exclusion; after scanning title & abstract (no; no)	1	no	0	no	0	c, e
436	Valdes, K., et al. (2014). "Hand therapist use of patient report outcome (PRO) in practice: A survey study" Journal of Hand Therapy Volume 27, Issue 4, Pages 299–308 http://www.jhandtherapy.org/article/S0894-1130(14)00076-3/abstract	added by Seidel	no	0	no	0	0	0	0	0	exclusion; after scanning title & abstract (no; no)	1	no	0	no	0	c, d, e
437	van der Molen HF, Lehtola MM, Lappalainen J, Hoonakker PLT, Hsiao H, Haslam R, Hale AR, Frings-Dresen MHW,Verbeek JH. Interventions to prevent injuries in construction workers. Cochrane Database of Systematic Reviews 2012, Issue 12. Art. No.: CD006251. DOI: 10.1002/14651858.CD006251.pub3.	CochraneWork_06_01_2017	no	0	no	0	0	0	0	0	exclusion; after scanning title & abstract (no; no)	1	no	0	no	0	c, d, e
438	van de Stadt, L. A., et al. (2010). "The value of ultrasonography in predicting arthritis in auto-antibody positive arthralgia patients: a prospective cohort study." Arthritis Res Ther 12(3): R98.	PubMed_03_02_2017	no	0	no	0	0	0	0	0	exclusion; after scanning title & abstract (no; no)	1	no	0	no	0	c, e
439	van Rijn, R. M., et al. (2009). "Associations between work-related factors and specific disorders at the elbow: a systematic literature review." Rheumatology (Oxford) 48(5): 528-536.	EMBASE_08_02_2017	yes	1	yes	1	3	2	2	1	inclusion for scanning full-text	5	yes	1	no	0	key paper
440	van Middelkoop, M., et al. (2015). "Incidence and Risk Factors for Upper Extremity Climbing Injuries in Indoor Climbers." Int J Sports Med 36(10): 837-842.	PubMed_03_02_2017	no	0	no	0	0	0	0	0	exclusion; after scanning title & abstract (no; no)	1	no	0	no	0	b, c, d, e
441	Van Sonhonven, F., Eric Geusens, and Stefaan Nijs. "Osteochondrosis dissecans of the elbow." Journal Belge de Radiologie 92.4 (2009): 207.	EMBASE_08_02_2017	no	0	no	0	0	0	0	0	exclusion; after scanning title & abstract (no; no)	1	no	0	no	0	c, e
442	van Vilsteren M, van Oostrom SH, de Vet HCW, Franche RL, Boot CRL, Anema JR. Workplace interventions to prevent work disability in workers on sick leave. Cochrane Database of Systematic Reviews 2015, Issue 10. Art. No.: CD006955. DOI: 10.1002/14651858.CD006955.pub3.	CochraneWork_06_01_2017	no	0	no	0	0	0	0	0	exclusion; after scanning title & abstract (no; no)	1	no	0	no	0	c, e
443	Vance, M. C., et al. (2012). "The association of hemoglobin A1c with the prevalence of stenosing flexor tenosynovitis." J Hand Surg Am 37(9): 1765-1769.	PubMed_03_02_2017	no	0	no	0	0	0	0	0	exclusion; after scanning title & abstract (no; no)	1	no	0	no	0	b, c, e
444	Vehmas, T., et al. (2013). "The relations of obesity indicators and early metabolic disturbance with upper extremity pain." Pain Med 14(7): 1081-1087.	EMBASE_08_02_2017	no	0	no	0	0	0	0	0	exclusion; after scanning title & abstract (no; no)	1	no	0	no	0	c, e
445	Verhagen AP, Bierma-Zeinstra SMA, Burdorf A, Stynes SM, de Vet HCW, Koes BW. Conservative interventions for treating work-related complaints of the arm, neck or shoulder in adults. Cochrane Database of Systematic Reviews 2013, Issue 12. Art. No.: CD008742. DOI: 10.1002/14651858.CD008742.pub2.	CochraneWork_06_01_2017	yes	1	no	0	1	1	1	1	discussion necessary	4	no	0	no	0	c, e
446	Vieira, E. R., Schneider, P., Guidera, C., Gadotti, I. C., & Brunt, D. (2016). Work-related musculoskeletal disorders among physical therapists: a systematic review. Journal of back and musculoskeletal rehabilitation, 29(3), 417-428.	added by Seidel	yes	1	yes	1	3	2	2	1	inclusion for scanning full-text	5	no	0	no	0	systematic review, no

																	single study
447	Vieira, E. R., et al. (2011). "Work disability benefits due to musculoskeletal disorders among Brazilian private sector workers." BMJ Open 1(1): e000003.	added by Seidel	no	0	no	0	0	0	0	0	exclusion; after scanning title & abstract (no; no)	1	no	0	no	0	c, e
448	Vimercati, L., et al. (2009). "Bilateral carpal tunnel syndrome and ulnar neuropathy at the elbow in a pizza chef." BMJ Case Rep 2009.	added by Seidel	no	0	no	0	0	0	0	0	exclusion; after scanning title & abstract (no; no)	1	no	0	no	0	b, c, e
449	Vincent JI, MacDermid JC, Michlovitz SL, Rafuse R, Wells-Rowsell C, Wong O, Bisbee L. The push-off test: development of a simple, reliable test of upper extremity weight-bearing capability. J Hand Ther. 2014 Jul-Sep;27(3):185-90; quiz 191. doi: 10.1016/j.jht.2014.03.002. PubMed PMID: 24794466.	EMBASE_08_02_2017	no	0	no	0	0	0	0	0	exclusion; after scanning title & abstract (no; no)	1	no	0	no	0	b, c, d, e
450	Violante, F. S., et al. (2016). "Carpal tunnel syndrome and manual work: the OCTOPUS cohort, results of a ten-year longitudinal study." Scand J Work Environ Health 42(4): 280-290.	added by Seidel	no	0	no	0	0	0	0	0	exclusion; after scanning title & abstract (no; no)	1	no	0	no	0	d, e
451	Waersted, M., et al. (2010). "Computer work and musculoskeletal disorders of the neck and upper extremity: a systematic review." BMC Musculoskelet Disord 11: 79.	added by Seidel	yes	1	yes	1	3	2	2	1	inclusion for scanning full-text	5	yes	1	no	0	systematic review, no single study
452	Walker-Bone, K., et al. (2012). "Occupation and epicondylitis: a population-based study." Rheumatology (Oxford) 51(2): 305-310.	PubMed_03_02_2017/ EMBASE_08_02_2017	yes	1	yes	1	3	2	2	1	inclusion for scanning full-text	5	yes	1	yes	1	no
453	Wang, A. W. and S. Erak (2007). "Fractional lengthening of forearm extensors for resistant lateral epicondylitis." ANZ J Surg 77(11): 981-984.	EMBASE_08_02_2017	no	0	no	0	0	0	0	0	exclusion; after scanning title & abstract (no; no)	1	no	0	no	0	b, c, e
454	Wang, K., et al. (2013). "Risk factors in idiopathic adhesive capsulitis: a case control study." J Shoulder Elbow Surg 22(7): e24-29.	PubMed_03_02_2017	no	0	no	0	0	0	0	0	exclusion; after scanning title & abstract (no; no)	1	no	0	no	0	b, d, e
455	Wang, D., Dai, F., & Ning, X. (2015). Risk assessment of work-related musculoskeletal disorders in construction: state-of-the-art review. Journal of Construction Engineering and management, 141(6), 04015008.	added by Seidel	no	0	no	0	0	0	0	0	exclusion; after scanning title & abstract (no; no)	1	no	0	no	0	c, e
456	Wang, L. H., et al. (2016). "The effects of forearm fatigue on baseball fastball pitching, with implications about elbow injury." J Sports Sci 34(12): 1182-1189.	PubMed_03_02_2017	no	0	no	0	0	0	0	0	exclusion; after scanning title & abstract (no; no)	1	no	0	no	0	b, c, e
457	Wang, P. C., Rempel, D. M., Harrison, R. J., Chan, J., & Ritz, B. R. (2007). Work-organisational and personal factors associated with upper body musculoskeletal disorders among sewing machine operators. Occupational and environmental medicine, 64(12), 806-813.	added by Seidel	yes	1	yes	1	3	2	2	1	inclusion for scanning full-text	5	no	0	no	0	e
458	Watts, A. C. & Bain, G. (2009). Patient-Rated Outcome of Ulnar Nerve Decompression: A Comparison of Endoscopic and Open In Situ Decompression. Journal of Hand Surgery Volume 34, Issue 8, Pages 1492–1498	added by Seidel	no	0	no	0	0	0	0	0	exclusion; after scanning title & abstract (no; no)	1	no	0	no	0	b, c, e

459	Waryasz GR, Daniels AH, Gil JA, Suric V, Eberson CP. Personal Trainer Demographics, Current Practice Trends and Common Trainee Injuries. Orthop Rev (Pavia). 2016 Oct 3;8(3):6600. PubMed PMID: 27761219; PubMed Central PMCID:PMC5066109.	EMBASE_08_02_2017	no	0	no	0	0	0	0	0	exclusion; after scanning title & abstract (no; no)	1	no	0	no	0	c, e
460	Whibley D, Martin KR, Lovell K, Jones GT. A systematic review of prognostic factors for distal upper limb pain. Br J Pain. 2015 Nov;9(4):241-55. doi: 10.1177/2049463715590885. PubMed PMID: 26526466; PubMed Central PMCID: PMC4616981.	EMBASE_08_02_2017	no	0	no	0	0	0	0	0	exclusion; after scanning title & abstract (no; no)	1	no	0	no	0	c, e
461	Whiteside, D., et al. (2016). "Predictors of Ulnar Collateral Ligament Reconstruction in Major League Baseball Pitchers." Am J Sports Med 44(9): 2202-2209.	added by Seidel	no	0	no	0	0	0	0	0	exclusion; after scanning title & abstract (no; no)	1	no	0	no	0	b, c, e
462	Widanarko B, Legg S, Devereux J, Stevenson M. The combined effect of physical, psychosocial/organisational and/or environmental risk factors on the presence of work-related musculoskeletal symptoms and its consequences. Appl Ergon. 2014 Nov;45(6):1610-21. doi: 10.1016/j.apergo.2014.05.018. PubMed PMID: 24934982.	EMBASE_08_02_2017_Double	yes	1	yes	1	3	2	2	1	inclusion for scanning full-text	5	yes	1	no	0	d
463	Wiggers JK, Helmerhorst GT, Brouwer KM, Niekel MC, Nunez F, Ring D. Injury complexity factors predict heterotopic ossification restricting motion after elbow trauma. Clin Orthop Relat Res. 2014 Jul;472(7):2162-7. PubMed PMID: 24078170; PubMed Central PMCID: PMC4048434.	EMBASE_08_02_2017	no	0	no	0	0	0	0	0	exclusion; after scanning title & abstract (no; no)	1	no	0	no	0	b, c, d, e
464	Wiggers, J. K., et al. (2012). "Predictors of diagnosis of ulnar neuropathy after surgically treated distal humerus fractures." J Hand Surg Am 37(6): 1168-1172.	EMBASE_08_02_2017	no	0	no	0	0	0	0	0	exclusion; after scanning title & abstract (no; no)	1	no	0	no	0	b, c, d, e
465	Wilhelmus Johannes Andreas, G., Wernstedt, P., & Campo, M. (2011). Work-related musculoskeletal disorders in female Swedish physical therapists with more than 15 years of job experience: prevalence and associations with work exposures. Physiotherapy theory and practice, 27(3), 213-222.	added by Seidel	yes	1	yes	1	3	2	2	1	inclusion for scanning full-text	5	no	0	no	0	e
466	Wilke, C., et al. (2011). "Motor activity as a way of preventing musculoskeletal problems in string musicians." Med Probl Perform Art 26(1): 24-29.	EMBASE_08_02_2017	no	0	no	0	0	0	0	0	exclusion; after scanning title & abstract (no; no)	1	no	0	no	0	c, e
467	Wilson AT, Pidgeon TS, Morrell NT, DaSilva MF. Trends in Revision Elbow Ulnar Collateral Ligament Reconstruction in Professional Baseball Pitchers. J Hand Surg Am. 2015 Nov;40(11):2249-54. doi: 10.1016/j.jhsa.2015.07.024. PubMed PMID: 26328904.	EMBASE_08_02_2017	no	0	no	0	0	0	0	0	exclusion; after scanning title & abstract (no; no)	1	no	0	no	0	b, c, d, e
468	Wilson d'Almeida, K., et al. (2008). "Sickness absence for upper limb disorders in a French company." Occup Med (Lond) 58(7): 506-508.	PubMed_03_02_2017/ EMBASE_08_02_2017	no	0	no	0	0	0	0	0	exclusion; after scanning title & abstract (no; no)	1	no	0	no	0	c, e
469	Winski, T. A. A systematic literature review: What are the sources of risk to health associated with new technologies in the office environment?.	added by Seidel	no	0	no	0	0	0	0	0	exclusion; after scanning title & abstract (no; no)	1	no	0	no	0	c, e
470	Wolf, B. R., et al. (2009). "Injury patterns in Division I collegiate swimming." Am J Sports Med 37(10): 2037-2042.	EMBASE_08_02_2017	no	0	no	0	0	0	0	0	exclusion; after scanning title & abstract (no; no)	1	no	0	no	0	b, c, d, e
471	Wolf, J. M., et al. (2009). "Incidence of carpal tunnel syndrome in the US military population." Hand (N Y) 4(3): 289-293.	added by Seidel	no	0	no	0	0	0	0	0	exclusion; after scanning title & abstract (no; no)	1	no	0	no	0	d, e

472	Wolf, J. M., et al. (2009). "Incidence of de Quervain's tenosynovitis in a young, active population." J Hand Surg Am 34(1): 112-115.	PubMed_03_02_2017	no	0	no	0	0	0	0	0	exclusion; after scanning title & abstract (no; no)	1	no	0	no	0	d, e
473	Wolf, J. M., et al. (2010). "Epidemiology of lateral and medial epicondylitis in a military population." Mil Med 175(5): 336-339.	EMBASE_08_02_2017	no	0	no	0	0	0	0	0	exclusion; after scanning title & abstract (no; no)	1	no	0	no	0	c, e
474	Wright, C., et al. (2014). "Who develops carpal tunnel syndrome during pregnancy: An analysis of obesity, gestational weight gain, and parity." Obstet Med 7(2): 90-94.	added by Seidel	no	0	no	0	0	0	0	0	exclusion; after scanning title & abstract (no; no)	1	no	0	no	0	b, c, d, e
475	Wroblewski, Andrew P., Hector A. Mejia, and Vonda J. Wright. "Application of platelet-rich plasma to enhance tissue repair." Operative Techniques in Orthopaedics 20.2 (2010): 98-105.	EMBASE_08_02_2017	no	0	no	0	0	0	0	0	exclusion; after scanning title & abstract (no; no)	1	no	0	no	0	b, c, d, e
476	Wymore, L. and J. Fronek (2015). "Shoulder functional performance status of National Collegiate Athletic Association swimmers: baseline Kerlan-Jobe Orthopedic Clinic scores." Am J Sports Med 43(6): 1513-1517.	PubMed_03_02_2017	no	0	no	0	0	0	0	0	exclusion; after scanning title & abstract (no; no)	1	no	0	no	0	b, c, d, e
477	Yamada, E. and D. C. Thomas (2011). "Common musculoskeletal diagnoses of upper and lower extremities in older patients." Mt Sinai J Med 78(4): 546-557.	EMBASE_08_02_2017	no	0	no	0	0	0	0	0	exclusion; after scanning title & abstract (no; no)	1	no	0	no	0	b, c, d, e
478	Yamazaki, H., et al. (2008). "Extensor tendon rupture associated with osteoarthritis of the distal radioulnar joint." J Hand Surg Eur Vol 33(4): 469-474.	PubMed_03_02_2017	no	0	no	0	0	0	0	0	exclusion; after scanning title & abstract (no; no)	1	no	0	no	0	b, c, d, e
479	Yang, Z. and T. W. Cheung (2016). "The inclusion of homemakers as an occupation amongst people with upper limb repetitive stress injuries." Work 55(1): 181-186.	added by Seidel	no	0	no	0	0	0	0	0	exclusion; after scanning title & abstract (no; no)	1	no	0	no	0	b, c, e
480	Yoshii, Y., et al. (2015). "Measurement of wrist flexion and extension torques in different forearm positions." Biomed Eng Online 14: 115.	PubMed_03_02_2017	yes	1	no	0	1	1	1	1	discussion necessary	4	yes	1	no	0	c, e
481	You, D., et al. (2014). "Meta-analysis: association between wrist posture and carpal tunnel syndrome among workers." Saf Health Work 5(1): 27-31.	added by Seidel	no	0	no	0	0	0	0	0	exclusion; after scanning title & abstract (no; no)	1	no	0	no	0	d, e
482	Yu, D., et al. (2012). "Quantitative posture analysis of 2D, 3D, and optical microscope visualization methods for microsurgery tasks." Work 41 Suppl 1: 1944-1947.	PubMed_03_02_2017	yes	1	no	0	1	1	1	1	discussion necessary	4	no	0	no	0	d, e
483	Yang, Y., Zhao, X., Dong, T., Du, C., Zhang, Y., & Zhang, Q. (2016). A meta-analysis of risk factors for heterotopic ossification after elbow trauma. Int J Clin Exp Med, 9(3), 5308-5317.	EMBASE_08_02_2017	no	0	no	0	0	0	0	0	exclusion; after scanning title & abstract (no; no)	1	no	0	no	0	d, e
484	Yung, M., et al. (2012). "Variation of force amplitude and its effects on local fatigue." Eur J Appl Physiol 112(11): 3865-3879.	PubMed_03_02_2017	yes	1	yes	1	3	2	2	1	inclusion for scanning full-text	5	no	0	no	0	d
485	Zeng, Q. Y., et al. (2010). "Epidemiologic study of soft tissue rheumatism in Shantou and Taiyuan, China." Chin Med J (Engl) 123(15): 2058-2062.	PubMed_03_02_2017/ EMBASE_08_02_2017	no	0	yes	1	2	1	1	1	discussion necessary	4	no	0	no	0	b
486	Zhang, S., et al. (2016). "Cost-Minimization Analysis of Open and Endoscopic Carpal Tunnel Release." J Bone Joint Surg Am 98(23): 1970-1977.	added by Seidel	no	0	no	0	0	0	0	0	exclusion; after scanning title & abstract (no; no)	1	no	0	no	0	b, c, d, e

487	Title: Tennis elbow: Subject matter of concern.	EMBASE_08_02_2017_Reference_not found	no	0	no	0	0	0	0	0	exclusion; after scanning title & abstract (no; no)	1	no	0	no	0	a
488	Title: Heterotrophic ossification following a elbow dislocation and coronoid process fracture causing significant morbidity.	EMBASE_08_02_2017_Reference_not found	no	0	no	0	0	0	0	0	exclusion; after scanning title & abstract (no; no)	1	no	0	no	0	a
489	Title: Evaluation of the unified approach to radial tunnel syndrome and tennis elbow syndrome.	EMBASE_08_02_2017_Reference_not found	no	0	no	0	0	0	0	0	exclusion; after scanning title & abstract (no; no)	1	no	0	no	0	a
490	Title: The use of growth mixture models to characterise recovery trajectories of patients with tennis elbow.	EMBASE_08_02_2017_Reference_not found	no	0	no	0	0	0	0	0	exclusion; after scanning title & abstract (no; no)	1	no	0	no	0	a
491	Title: Hypovitaminosis D in postmenopausal women with a distal radius fracture.	EMBASE_08_02_2017_Reference_not found	no	0	no	0	0	0	0	0	exclusion; after scanning title & abstract (no; no)	1	no	0	no	0	a
492	Title: A rare complication of eswt application in a patient with lateral epicondylitis ESWT Uygulanan Lateral Epikondilitli Hastada Gelisen Nadir Bir Komplikasyon.	EMBASE_08_02_2017_Reference_not found	no	0	no	0	0	0	0	0	exclusion; after scanning title & abstract (no; no)	1	no	0	no	0	a
493	Title: Investigation of the effectiveness of laser in the treatment of lateral epicondylitis Lateral Epikondilit Tedavisinde Lazerin Etkinli inin Arastirilmasi.	EMBASE_08_02_2017_Reference_not found	no	0	no	0	0	0	0	0	exclusion; after scanning title & abstract (no; no)	1	no	0	no	0	a
494	Title: Coexistence of lateral epicondylitis and ulnar nerve entrapment Lateral Epikondilit ve Ulnar Tuzak Noropati Birlikteli i.	EMBASE_08_02_2017_Reference_not found	no	0	no	0	0	0	0	0	exclusion; after scanning title & abstract (no; no)	1	no	0	no	0	a

0		322	322	340	340	18	463	484
1		63	78	85	154	304	31	10
2		15	94	69		18		
3		94				25		
4						60		
5						69		
	∑	494	494	494	494	494	494	494

Statistics	n
CochraneWork_06_01_2017	10
PubMed_03_02_2017	116
EMBASE_08_02_2017	167
PubMed_03_02_2017/EMBASE_08_02_2017	25
EMBASE_08_02_2017_Double	5
added by Seidel	147
EMBASE_08_02_2017_Abstract_not found	16
EMBASE_08_02_2017_Reference_not found	8

Doubles	30
Cochrane Work	10

Exclusion		BS		total
		no = 0	Yes = 1	
DHS	no = 0	322	15	337
	yes = 1	63	94	157
total		385	109	494
p0	=	0.8421 (84,2% initial agreement)		
cohens kappa	=	0.6035		
pe	=	0.6018		
K	=	0.6035		

Pubmed	141
EMBASE	226
added by Seidel	147
Total	524
Total without double	494
Exclusion after scanning title & abstract	322
scanning full text	172
Exclusion after scanning full text	141
Exclusion after scanning full text	463
checking for eligibility	31
Exclusion after checking for eligibility	21
Exclusion in total	484
Included	10

File VII – Table S5: All relevant results of all included studies (incl. study attributes)

Table S5. All relevant results of all included studies (incl. study attributes).

Reference Author (year)	Design	Population / Sample	Exposure Assessment	Outcome Assessment	No.	Risk factor	Outcome	Gender	Measure (CI 95%)		Adjustment	Significance	Different risk factor specifications associated with at least one disorder 1 = non-significant; 2 = significant 3 = same risk factor as reported in previous study	Additional declaration
Descatha et al. (2013) [36]	CH	1107 newly employed worker in St. Louis, USA<br><br>(76 cases at baseline, 699 participants completed follow up)<br><br>(at follow up: LE (n = 34), ME (n = 30), LE/ME (n = 48), LE or ME (n = 16))	self-reported physical work exposures and psycho-social measures via question-naire<br><br>responses categorized by authors into 4 categories (none or less than 1 hour/day, 1-2 hours/day, 2-4 hours/day, ≥ 4 hours/day)	Question-naire<br><br>+ physical examination		Frequently wrist bending or twisting on average								"... "bending" (On average, how long altogether each day did you frequently bend or twist your hands or wrists?)<br><br>"rotating" (On average, how long altogether each day did you do tasks where there was a rotating, twisting or screwing motion of the forearm?), and<br><br>"gripping" (On average, how long altogether each day did you use your hand in a forceful grip?).
					#58	1-2 hours/day	LE		OR	0.80 (0.10, 7.40)	a)	/	1	
							LE / ME		OR	2.50 (0.60, 11.40)	a)	/		
					#13	2-4 hours/day	LE		OR	2.80 (0.70, 10.50)	a)	/	2	
							ME		OR	4.90 (1.10, 20.70)	a)	*		
							LE / ME		OR	3.90 (1.10, 13.80)	a)	*		
					#14	≥ 4hours/day	LE		OR	4.40 (1.50, 13.10)	a)	*	2	
							ME		OR	8.20 (2.40, 27.90)	a)	*		
							LE / ME		OR	6.90 (2.40, 19.90)	a)	*		
						Forearm rotating (also twisting, or screwing motion)								
					#68	1-2 hours/day	LE		OR	1.00 (0.20, 4.60)	a)	/	1	
							ME		OR	0.50 (0.10, 3.90)	a)	/		
							LE / ME		OR	1.00 (0.30, 3.60)	a)	/		
					#69	2-4 hours/day	LE		OR	2.30 (0.80, 6.70)	a)	/	1	
							ME		OR	2.80 (1.00, 7.70)	a)	/		
							LE / ME		OR	2.60 (1.10, 6.30)	a)	/		
					#22	≥ 4 hours/day	LE		OR	2.70 (1.20, 6.20)	a)	*	2	
							ME		OR	2.50 (1.00, 5.80)	a)	/		
							LE / ME		OR	2.70 (1.30, 5.40)	a)	*		
						Hand in forceful grip on average								
					#46	1-2 hours/day	LE		OR	1.30 (0.40, 4.20)	a)	/	1	
							ME		OR	2.10 (0.60, 7.20)	a)	/		
							LE / ME		OR	1.70 (0.60, 4.50)	a)	/		
					#47	2-4 hours/day	LE		OR	1.50 (0.50, 4.30)	a)	/	1	
							ME		OR	1.90 (0.50, 6.50)	a)	/		
							LE / ME		OR	1.50 (0.60, 4.00)	a)	/		
					#2	≥ 4 hours/day	LE		OR	1.70 (0.70, 4.00)	a)	/	2	
							ME		OR	3.80 (1.50, 9.60)	a)	*		
							LE / ME		OR	2.80 (1.40, 5.80)	a)	*		
						Frequently wrist bending ≥ 4h/day and forearm rotating on average								

					#15	≥ 2h/day	LE		OR	2.50 (1.10, 5.30)	b)	*	2	
							ME		OR	3.10 (1.40, 6.80)	b)	*		
							LE / ME		OR	3.00 (1.60, 5.80)	b)	*		
							LE / ME	Men	OR	2.80 (1.20, 6.20)	b)	*		
							LE / ME	Women	OR	3.60 (1.20, 11.00)	b)	*		
Fan et al. (2009) [31]	CSS	733 worker (695 non-cases, 38 LE cases) in manufacturing sectors in western Washington State	observation and videotaping (2 synchronized cameras with different positions), measurements for biomechanical exposures, self-administered psychosocial questionnaire for psychosocial factors time-based posture analysis via software, based on video frames; frequency and percent of time (duty cycle) of forceful exertions based on time-studies object weight and push or pull forces measured	structured interviews and specific body map was used + physical examination		Frequency of forceful exertions (times/min)								high - high: high force – high posture at median<br><br>intermediate: low on either posture or force and high on the other<br><br>(high force: pinch grip > 9N; power grip, push/pull or lifting force> 44N)<br><br>Forceful exertion (pinch grip force ≥ 8.9 N (2 lbs/ 0.9 kg);<br><br>power grip forces, lifting object weights or push/ pull forces ≥ 44.1 N (10 lbs/ 4.5 kg)<br><br>Bao & Silverstein (2005) [43]
					#29	≤ 1 to < 5 vs. < 1	LE		OR	4.47 (1.57, 13.71)	d)	*	2	
					#30	≥ 5 vs. < 1	LE		OR	5.17 (1.78, 15.02)	d)	*	2	
						Duty cycle of forceful exertions (% time)								
					#31	≤ 3 to < 15 %	LE		OR	3.36 (1.28, 8.84)	i)	*	2	
					#32	≥ 15 %			OR	3.00 (1.13, 7.96)	i)	*	2	
						Frequency of shoulder movement (times/min)								
					#55	≤ 10 to < 20	LE		OR	2.03 (0.73, 5.66)	i)	/	1	
					#56	≥ 20	LE		OR	2.70 (0.96, 7.63)	i)	/	1	
						Forceful lifting, time-weighted average (% time)								
					#6	> 0 %	LE		OR	2.65 (1.21, 5.83)	i)	*	2	
						Frequency of forceful lifting, time-weighted average (times/min)								
					#53	< 0 to < 2	LE		OR	2.30 (0.95, 5.59)	i)	/	1	
					#7	≥ 2	LE		OR	3.06 (1.28, 7.27)	i)	*	2	
						Forearm supination ≥ 45° (% time)								
					#26	≥ 5 %	LE		OR	2.25 (1.13, 4.50)	i)	*	2	
						Wrist flexion or extension ≥ 45° (% time)								
					#63	≥ 1 %	LE		OR	0.66 (0.34, 1.27)	i)	/	1	
						Wrist radial deviation < 5° or ulnar deviation ≥ 20° (% time)								
					#59	≥ 4 %	LE		OR	0.62 (0.32, 1.22)	i)	/	1	
						Forearm supination ≥ 45° and forceful lifting (% time)								
					#91	Intermediate	LE		OR	1.21 (0.44, 3.38)	i)	/	1	
					#33	High - high	LE		OR	3.65 (1.47, 9.07)	i)	*	2	
						Forearm supination (≥ 45°) ≥ 5% of time (duty cycle)								
					#92	or forceful lifting (> 0 % time)	LE		OR	1.09 (0.38, 3.09)	d)	/	1	
					#34	and forceful lifting (> 0 % time)	LE		OR	2.98 (1.18, 7.55)	d)	*	2	

			using force gauges<br>+ using a force matching method										
Fan et al. (2014) [37]	CH	733 worker at baseline, 611 at follow up,<br><br>(57 cases and 554 non-cases, cases at follow up)<br><br>in manufacturing sectors in western Washington State	observation and videotaping, measurements for biomechanical exposures,<br><br>self-administered psychosocial questionnaire for psychosocial factors<br><br>time-based posture analysis via software, based on video frames;<br><br>frequency and percent of time (duty cycle) of forceful exertions based on time-studies<br><br>forceful exertions measured via force gauges (object	structured interviews<br><br>+ physical examinations		Wrist flexion/extension ≥ 15° for							Forceful exertions: pinch grip force ≥ 0.9 kg of object weight/ 1.8-kg pinch grip force power grip forces: ≥ 4.5 kg of object weight / 4.5-kg power grip, lifting/ lowering as object weights ≥ 4.5 kg, and pushing/pulling forces as ≥ 4.5 kg force<br>Bao et al. (2006a) [42]<br><br>Forceful exertion included lifting/lowering, pushing/pulling, power gripping, pinch gripping, other types of forceful hand exertions (e.g., thumb press, one hand pull) [37],<br><br>awkward postures: wrist flexion or extension ≥ 15°, forearm supination ≥ 45°, forearm pronation ≥ 45°, and forearm rotation (either forearm supination or pronation) ≥ 45°<br>Bao et al. (2006b) [44]<br><br>forearm rotation = forearm supination or forearm pronation<br><br>qualitative specification: any pinch grip fore (LE; HR 1.20 (0.64, 2.24); a); /)
					#60	≥ 40% time	LE	HR	0.94 (0.56, 1.58)	a)	/	1	
						Wrist flexion/extension ≥ 45° for							
					#61	≥ 2% time	LE	HR	0.98 (0.58, 1.66)	a)	/	1	
						Forearm pronation ≥ 45° for							
					#64	≥ 40% time	LE	HR	1.60 (0.93, 2.73)	a)	/	1	
						Forearm supination ≥ 45° for							
					#26	≥ 5% time	LE	HR	1.19 (0.71, 2.00)	a)	/	3	
						Forearm rotation ≥ 45° for							
					#65	≥ 45% time	LE	HR	1.41 (0.82, 2.42)	a)	/	1	
						Lifting							
					#50	≥ 3% time	LE	HR	1.28 (0.76, 2.15)	a)	/	1	
						Duty cycle							
					#54	≥ 10% time	LE	HR	1.43 (0.84, 2.43)	a)	/	1	
						Frequency of forceful exertion for							
					#90	≥ 2 times/min	LE	HR	1.18 (0.69, 2.00)	a)	/	1	
						Wrist flexion/extension ≥ 15° for							
					#93	≥ 40% time and any power grip	LE	HR	1.52 (0.78, 2.96)	a)	/	1	
					#94	≥ 40% time and no power grip	LE	HR	0.77 (0.40, 1.50)	a)	/	1	
					#95	< 40% time and any power grip	LE	HR	1.32 (0.55, 3.15)	a)	/	1	
						Wrist flexion/extension ≥ 15° for							
					#96	≥ 40% time and lifting ≥ 3% time	LE	HR	1.18 (0.60, 2.33)	a)	/	1	
					#97	≥ 40% time and lifting < 3% time	LE	HR	0.74 (0.36, 1.50)	a)	/	1	
					#98	< 40% time and lifting ≥ 3% time	LE	HR	0.96 (0.43, 2.13)	a)	/	1	
						Wrist flexion/extension ≥ 15° for							
					#71	≥ 40% time and duty cycle ≥ 10% time	LE	HR	1.30 (0.66, 2.54)	a)	/	1	
					#72	≥ 40% timeand duty cycle < 10% time	LE	HR	0.68 (0.33, 1.43)	a)	/	1	
					#73	< 40% time and duty cycle ≥ 10% time	LE	HR	0.99 (0.45, 2.20)	a)	/	1	
						Wrist flexion/extension ≥ 15° for							
					#119	≥ 40% time and Freq force ≥ 2/min	LE	HR	1.09 (0.56, 2.13)	a)	/	1	
					#120	≥ 40% time and Freq force < 2/min	LE	HR	0.67 (0.33, 1.36)	a)	/	1	
					#121	< 40% time and Freq force ≥ 2/min	LE	HR	0.77 (0.34, 1.74)	a)	/	1	
						Wrist flexion/extension ≥ 45° for							

			weight and push/pull forces)<br><br>+ grip dynamometers (pinch and power grip<br><br>+ using a force matching method)		#99	≥ 5% time and any power grip	LE	HR	1.39 (0.67, 2.89)	a)	/	1	
					#100	≥ 5% time and no power grip	LE	HR	0.91 (0.46, 1.79)	a)	/	1	any power grip fore (LE; HR 1.65 (0.90, 2.82); a); /)
					#101	< 5% time and any power grip	LE	HR	1.94 (0.85, 4.39)	a)	/	1	
						Wrist flexion/extension ≥ 45° for							
					#102	≥ 5% time and lifting ≥ 3% time	LE	HR	1.11 (0.54, 2.27)	a)	/	1	
					#103	≥ 5% time and lifting < 3% time	LE	HR	0.93 (0.45, 1.95)	a)	/	1	
					#104	< 5% time and lifting ≥ 3% time	LE	HR	1.52 (0.68, 3.43)	a)	/	1	
						Wrist flexion/extension ≥ 45° for							
					#74	≥ 5% time and duty cycle ≥ 10% time	LE	HR	1.22 (0.60, 2.50)	a)	/	1	
					#75	≥ 5% time and duty cycle < 10% time	LE	HR	1.00 (0.47, 2.11)	a)	/	1	
					#76	< 5% time and duty cycle ≥ 10% time	LE	HR	2.06 (0.91, 4.66)	a)	/	1	
						Wrist flexion/extension ≥ 45° for							
					#122	≥ 5% time and Freq force ≥ 2/min	LE	HR	1.02 (0.51, 2.04)	a)	/	1	
					#123	≥ 5% time and Freq force < 2/min	LE	HR	0.86 (0.41, 1.77)	a)	/	1	
					#124	< 5% time and Freq force ≥ 2/min	LE	HR	1.28 (0.55, 3.02)	a)	/	1	
						Forearm pronation ≥ 45° for							
					#38	≥ 40% time and any power grip	LE	HR	2.80 (1.35, 5.77)	e)	**	2	
					#105	≥ 40% time and no power grip	LE	HR	1.97 (1.00, 3.89)	e)	/	1	
					#106	< 40% time and any power grip	LE	HR	1.65 (0.69, 3.96)	e)	/	1	
						Forearm pronation ≥ 45° for							
					#39	≥ 40% time and lifting ≥ 3% time	LE	HR	2.50 (1.19, 5.24)	e)	*	2	
					#107	≥ 40% time and lifting < 3% time	LE	HR	1.98 (0.96, 4.07)	e)	/	1	
					#108	< 40% time and lifting ≥ 3% time	LE	HR	1.58 (0.77, 3.22)	e)	/	1	
						Forearm pronation ≥ 45° for							
					#25	≥ 40% time and duty cycle ≥ 10% time	LE	HR	2.25 (1.09, 4.66)	e)	*	2	
					#77	≥ 40% time and duty cycle < 10% time	LE	HR	1.61 (0.76, 3.39)	e)	/	1	
					#78	< 40% time and duty cycle ≥ 10% time	LE	HR	1.76 (0.85, 3.62)	e)	/	1	
						Forearm pronation ≥ 45° for							
					#125	≥ 40% time and Freq force ≥ 2/min	LE	HR	2.28 (1.00, 5.19)	a)	/	1	
					#126	≥ 40% time and Freq force < 2/min	LE	HR	1.36 (0.63, 2.94)	a)	/	1	
					#127	< 40% time and Freq force ≥ 2/min	LE	HR	1.03 (0.44, 2.42)	a)	/	1	
						Forearm supination ≥ 45° for							
					#109	≥ 5% time and any power grip	LE	HR	1.48 (0.62, 3.55)	a)	/	1	
					#110	≥ 5% time and no power grip	LE	HR	1.86 (0.96, 3.60)	a)	/	1	
					#36	< 5% time and any power grip	LE	HR	2.86 (1.41, 5.82)	a)	**	2	
						Forearm supination ≥ 45° for							
					#111	≥ 5% time and lifting ≥ 3% time	LE	HR	1.32 (0.66, 2.62)	a)	/	1	
					#112	≥ 5% time and lifting < 3% time	LE	HR	1.89 (0.92, 3.90)	a)	/	1	
					#35	< 5% time and lifting ≥ 3% time	LE	HR	2.09 (1.02, 4.27)	a)	*	2	

						Forearm supination ≥ 45° for								
					#79	≥ 5% time and duty cycle ≥ 10% time	LE		HR	1.47 (0.74, 2.93)	a)	/	1	
					#80	≥ 5% time and duty cycle < 10% time	LE		HR	1.59 (0.76, 3.34)	a)	/	1	
					#81	< 5% time and duty cycle ≥ 10% time	LE		HR	2.02 (0.98, 4.13)	a)	/	1	
						Forearm supination ≥ 45° for								
					#128	≥ 5% time and Freq force ≥ 2/min	LE		HR	1.29 (0.66, 2.51)	a)	/	1	
					#129	≥ 5% time and Freq force < 2/min	LE		HR	1.36 (0.65, 2.82)	a)	/	1	
					#130	< 5% time and Freq force ≥ 2/min	LE		HR	1.35 (0.64, 2.83)	a)	/	1	
						Forearm rotation ≥ 45° for								
					#37	≥ 45% time and any power grip	LE		HR	2.83 (1.16, 6.90)	a)	*	2	
					#113	≥ 45% time and no power grip	LE		HR	1.88 (0.83, 4.28)	a)	/	1	
					#114	< 45% time and any power grip	LE		HR	2.31 (0.82, 6.53)	a)	/	1	
						Forearm rotation ≥ 45° for								
					#115	≥ 45% time and lifting ≥ 3% time	LE		HR	2.27 (0.88, 5.88)	a)	/	1	
					#116	≥ 45% time and lifting < 3% time	LE		HR	1.50 (0.58, 3.84)	a)	/	1	
					#117	< 45% time and lifting ≥ 3% time	LE		HR	1.25 (0.43, 3.61)	a)	/	1	
						Forearm rotation ≥ 45° for								
					#24	≥ 45% time and duty cycle ≥ 10% time	LE		HR	3.10 (1.05, 9.15)	a)	*	2	
					#82	≥ 45% time and duty cycle < 10% time	LE		HR	2.20 (0.77, 6.30)	a)	/	1	
					#83	< 45% time and duty cycle ≥ 10% time	LE		HR	2.22 (0.70, 7.04)	a)	/	1	
						Forearm rotation ≥ 45° for								
					#131	≥ 45% time and Freq force ≥ 2/min	LE		HR	1.96 (0.80, 4.84)	a)	/	1	
					#132	≥ 45% time and Freq force < 2/min	LE		HR	1.52 (0.63, 3.66)	a)	/	1	
					#133	< 45% time and Freq force ≥ 2/min	LE		HR	1.20 (0.42, 3.37)	a)	/	1	
Herquelot, et al. (2013a) [32]	CSS	3710 adult, temporary and part-time worker in a French region<br><br>(90 LE cases in men, 39 LE cases in women)	self-administered questionnaire, physical work load assessed via Borg Rating of Perceived Exertion Scale<br><br>Responses categorized into 4-point Likert-type	physical examinations		Doing repetitive tasks								Elbow movements = elbow flexion/extension more than 2 h/day and wrist bending more than 2 h/day<br>hard = hard physical exertion;<br>Borg Rating of Perceived Exertion [47]<br>Scale (6 to 20); less hard exertion (6–13) and hard exertion to exhaustion (14–20),<br>qualitative specification: hard physical exertion: (LE; Men; OR; 2.57 (1.34, 4.90); adjusted for
					#9	> 4 h/day	LE	Men	OR	1.05 (0.54, 2.02)	l)	/	2	
							LE	Women	OR	2.46 (1.30, 4.65)	a)	**		
							LE	Women	OR	1.80 (0.91, 3.59)	l)	/		
						Elbow flexion/ extension								
					#21	> 2 h/day	LE	Men	OR	2.41 (1.38, 4.22)	a)	**	2	
							LE	Women	OR	2.65 (1.40, 5.02)	a)	**		
						Extreme wrist bending								
					#20	> 2 h/day	LE	Men	OR	2.27 (1.30, 3.97)	a)	**	2	
							LE	Women	OR	1.98 (1.04, 3.75)	a)	*		
						Use of vibrating handtools								
					#88	> 2 h/day	LE	Men	OR	0.95 (0.46, 1.97)	a)	/	1	
							LE	Women	OR	2.08 (0.62, 6.98)	a)	/		
						Elbow-specific combined physical exposure								

			scale,: never or practically never; rarely (< 2 h/day), often (2–4 h/day) and always (> 4 h/day)		#45	Light and 1 to 2 elbow movements	LE	Men	OR	1.34 (0.46, 3.90)	a)	/	1	individual characteristics, repetition, physical exertion, and social support, *) (LE; Women; OR; 2.51 (1.24, 5.08); adjusted for individual characteristics, repetition, physical exertion, and social support, *)
							LE	Men	OR	1.52 (0.52, 4.44)	l)	/		
							LE	Women	OR	0.53 (0.17, 1.60)	l)	/		
					#118	Hard and no elbow movement	LE	Men	OR	0.56 (0.11, 2.92)	a)	/	1	
							LE	Men	OR	0.76 (0.14, 3.98)	l)	/		
							LE	Women	OR	0.80 (0.24, 2.71)	l)	/		
					#41	Hard and 1 elbow movement	LE	Men	OR	3.78 (1.85, 7.70)	m)	**	2	
							LE	Women	OR	2.54 (1.12, 5.76)	m)	/		
					#42	Hard and 2 elbow movements	LE	Men	OR	5.27 (1.93, 14.37)	a)	***	2	
							LE	Men	OR	5.60 (2.76, 11.35)	m)	/		
							LE	Women	OR	2.89 (1.28, 6.51)	m)	/		
Herquelot, et al. (2013b) [38]	CH	3710 adult, temporary and part-time worker in a French region (1046 at follow up, 103 LE cases in men and 68 LE cases in women at follow up)	self-reported occupation-al exposures Responses categorized into 4-point Likert-type scale,: never or practically never; rarely (< 2 h/day), often (2–4 h/day) and always (> 4 h/day)	Standard-ized physical examina-tions (method-logy and clinical tests of the Saltsa consensus)		Doing repetitive tasks (> 4 h/day)								Elbow-specific combined physical exposure: High physical exertion with elbow flexion/ extension > 2 hours/day and extreme wrist bending > 2 hours/day
					#9	at first Questionnaire (at baseline investigation)	LE	Men	IRR	1.20 (0.40, 3.60)	n)	/	3	
							LE	Women	IRR	2.70 (1.00, 7.00)	n)	/		
					#9	at second Questionnaire (at follow up investigation)	LE	Men	IRR	1.20 (0.40, 3.50)	n)	/		
							LE	Women	IRR	1.00 (0.30, 3.80)	n)	/		
					#9	at both Questionnaires (at baseline and at follow up	LE	Men	IRR	2.80 (1.20, 6.20)	n)	*		
							LE	Women	IRR	2.20 (0.80, 6.30)	n)	/		
						Elbow-specific combined physical exposure								
					#42	at first Questionnaire (at baseline investigation)	LE	Women	IRR	1.50 (0.50, 4.70)	f)	/	3	
					#42	at second Questionnaire (at follow up investigation)	LE	Men	IRR	2.70 (1.10, 6.10)	f)	*		
					#42	at both Questionnaires (at baseline and at follow up	LE	Men	IRR	3.20 (1.50, 6.40)	f)	**		
							LE	Women	IRR	3.30 (1.40, 7.60)	f)	**		
Nordander et al. (2009) [33]	CSS	4961 adult worker (1241 males, 3720 females, worker with homogen-eous work tasks were grouped, 43 groups);	Homogen-eous jobs (similar work tasks) were grouped for job titles and authors classified them in 2 exposure	Nordic Question-naire in interview or as mailed question-naire + physical examination (only for		repetitive/constrained vs. varied/mobile work								Repetitive work: cycle time < 30 s or > 50% cycle time (involved the same fundamental cycle) Constrained work: > 50% of working time involved prolonged awkward posturesstatistically significant, 95% CI exceeds 1.0
					#43		LE	Men	PR	1.00 (0.30, 2.80)	c)	/	2	
							LE	Women	PR	1.90 (1.00, 3.80)	c)	/		
							ME	Men	PR	4.00 (1.10, 15.00)	c)	*		
							ME	Women	PR	3.50 (1.00, 12.00)	c)	/		
							Pronator	Women	PR	3.40 (0.40, 29.00)	c)	/		
							Radial	Men	PR	1.30 (0.10, 21.00)	c)	/		
							Radial	Women	PR	3.40 (0.80, 15.00)	c)	/		

		repetitive/ constrained work (female: 85 cases female, male: 19 cases); varied / mobile work (female: 18 cases, male: 13 cases)		some groups)			
Nordander et al. (2013) [34]	CSS	2652 adult worker<br><br>(761 males, 1891 females, 27 different groups, worker representing repetitive and/or constrained as well as varied/ mobile work)	wrist postures + movements measured using biaxial flexible electrogonio -meters; Flexion/ extension angles recorded via data logger; bipolar surface electro-myography (EMG) used to record the muscular load; psycho-social factors: Swedish version of the Job Content Questionnai re Ruben-owitz standard-ized question-naire,	Nordic Question-naire<br><br>+ physical examination	Wrist flexion β in [%/°]<br>#16 at -40° Flexion LE PR 0.30 (0.04, 0.60) g) *<br>ME PR 0.17 (-0.02, 0.40) g) /<br><br>#17 at -20° Flexion LE PR 0.04 (-0.07, 0.20) g) /<br>ME PR 0.08 (0.01, 0.10) g) *<br><br>#62 at 0° Flexion LE PR 0.05 (-0.06, 0.20) g) /<br>ME PR 0.05 (-0.02, 0.10) g) /<br><br>Wrist angular velocity β in [%/(°/s)]<br>#10 at 5°/s LE PR 0.00 (-0.08, 0.08) g) /<br>ME PR 0.10 (0.10, 0.20) g) *<br><br>Muscular activity β in [%/%MVE]<br>#48 at 1 % MVE LE PR -0.34 (-1.10, 0.50) g) /<br><br>#49 at 15 % MVE LE PR -0.02 (-0.20, 0.10) g) /<br>ME PR 0.03 (-0.09, 0.10) g) /	 2<br><br><br>2<br><br><br>1<br><br><br><br>2<br><br><br><br>1<br><br>1	Physical exposures recorded in subsample of workers in each group<br><br>β = sex-adjusted slope of the regression line

			Copenhagen Psycho-social Question-											
Spahn et al. (2016) [40]	CRS	197 adult worker;<br><br>71 cases (38 males, 33 females);<br><br>126 controls (89 males, 37 females)	Question-naire, interview	structured question-naire<br><br>+ physical examination<br><br>+ clinical-functional tests		Standing work								
					#84	> 1h/day	LE	Men	OR	2.40 (0.80, 6.70)	g)	/	1	
							LE	Women	OR	1.90 (0.40, 8.10)	g)	/		
						Sedentary work								
					#85	> 1h/day	LE	Men	OR	0.50 (0.20, 1.10)	g)	/	1	
							LE	Women	OR	0.50 (0.20, 2.10)	g)	/		
						PC work								
					#86	> 1h/day	LE	Men	OR	1.00 (0.30, 3.50)	g)	/	1	
							LE	Women	OR	0.30 (0.10, 0.80)	g)	/		
						Constant moving, lifting and carrying of loads								
					#51	> 10 kg	LE	Men	OR	2.20 (0.80, 6.10)	g)	/	1	
					#52	> 5 kg	LE	Women	OR	4.00 (0.90, 27.10)	g)	/	1	
						overhead working								
					#12	> 1h/day	LE	Men	OR	12.00 (3.20, 43.80)	g)	***	2	
							LE	Women	OR	1.00 (0.30, 4.20)	g)	/		
						Arm holding in front of body								
					#66	> 1h/day	LE	Men	OR	1.40 (0.40, 5.00)	g)	/	1	
							LE	Women	OR	2.20 (0.50, 9.20)	g)	/		
						Swinging movements with the arm								
					#67	> 1h/day	LE	Men	OR	2.60 (0.80, 8.80)	g)	/	1	
							LE	Women	OR	1.90 (0.40, 8.30)	g)	/		
						Vibration stress								
					#89	> 1h/day	LE	Men	OR	1.50 (0.50, 4.10)	g)	/	1	
						Maximum forceful efforts of the Hand								
					#1	> 1h/day	LE	Men	OR	6.90 (2.70, 17.50)	g)	***	2	
								Women	OR	9.60 (3.10, 30.40)	g)	***		
						Repetition (> 3 motion sequences /sec or minimum 10,000 / h)								
					#8	> 1h/day	LE	Men	OR	10.60 (4.00, 28.30)	g)	***	2	
								Women	OR	11.00 (2.60, 45.10)	g)	**		
						Forceful turning								
					#40	> 1h/day	LE	Men	OR	4.70 (1.40, 16.20)	g)	**	2	
							LE	Women	OR	1.40 (0.30, 6.80)	g)	/		
						Pat with the hand								

					#5	> 1h/day	LE	Men	OR	13.80 (2.90, 66.10)	g)	***	2	
						Maximum forceful efforts of the Hand and repetition								
					#28	> 1h/day	LE	Men	OR	14.70 (5.20, 41.50)	g)	***	2	
								Women	OR	29.30 (3.40, 34.80)	g)	***		
						Wrist Extension								
					#18	> 1h/day	LE	Men	OR	12.00 (3.00, 47.90)	g)	***	2	
								Women	OR	7.50 (1.80, 31.60)	g)	***		
						Wrist Flexion								
					#19	> 1h/day	LE	Men	OR	4.20 (1.20, 14.80)	g)	*	2	
							LE	Women	OR	1.20 (0.40, 3.60)	g)	/		
Svendsen et al. (2012) [39]	TCRS	4296 adult worker (546 patients with UN, 1450 referents)	mailed question-naire<br><br>+ exposure classify-cation via Job Exposure Matrix and experts' ratings	Neuro-physio-logical examination via electro-neuro-graphy		Repetition time (excluding computer use)								Repetion: (≥ 4 wrist or elbow movements /min), McAtamney & Nigel Corlett (1993) [45] excluding computer use<br><br>non-neutral postures of elbow (flexion > 100°, or ≥ near maximal pronation/supination) or wrist (> 5° radial deviation, > 10° ulnar deviation or > 15° palmar/dorsal flexion McAtamney & Nigel Corlett (1993), Thomsen et al. (2007) [45,46]; force score as maximal strength across a full working day (Moore & Garg (1995) [41]):<br>0 (mean force < 10% MVC (maximum voluntary contraction))<br>1 (10 to 29% MVC)
					#57	> 0 to < 2.5 h/day	UN		OR	0.85 (0.51, 1.41)	j)	/	1	
					#11	≥ 2.5 h/day	UN		OR	2.22 (1.41, 3.51)	j)	*	2	
						Nonneutral-posture-time								
					#70	≥ 1 to < 2 h/day	UN		OR	1.29 (0.82, 2.02)	j)	/	1	
					#23	≥ 2 h/day	UN		OR	1.82 (1.15, 2.89)	j)	*	2	
						Hand-arm vibration [HAV] - time (acceleration ≥ 3 m/s²)								
					#87	> 0 to < 1 h/day	UN		OR	1.97 (0.95, 4.10)	j)	/	1	
					#27	≥ 1h/day	UN		OR	2.19 (1.05, 4.56)	j)	*	2	
						Force score								
					#3	> 0 to < 1	UN		OR	2.73 (1.42, 5.25)	h)	*	2	
					#4	≥ 1	UN		OR	3.85 (2.04, 7.24)	h)	*	2	
Walker-Bone et al. (2012) [35]	CSS	6038 adult worker (45 LE cases, 34 ME cases)	self-defined exposures according to a carefully validated exposure list (ranging	Question-naire<br><br>+ standard-ized interview		repetitive bending/ straightening of the elbow								
					#44	> 1h/ day	LE		OR	2.50 (1.20, 5.30)	k)	*	2	
							ME		OR	5.30 (1.90, 14.90)	k)	**		

			from working with the neck bent/ twisted; working	+ physical examination			

Different non-significant risk factor specifications associated with at least one specific disorder of the elbow ∑	89
Different significant risk factor specifications associated with at least one specific disorder of the elbow ∑	44
Total same risk factor as reported in previous study associated with at least one specific disorder of the elbow ∑	3
Total different significant and non-significant risk factor specifications associated with at least one specific disorder of the elbow ∑	133

Study design: CRS = Case Referent Study; CH = Cohort Study; CSS = Cross-Sectional Study; TCRS = Triple Case Referent Study

Outcome: UN = ulnar neuropathy; LE = lateral epicondylitis; ME = medial epicondylitis, LE / ME = lateral and / or medial epicondylitis, Radial = Radial tunnel syndrome, Pronator = Pronator teres syndrome

Measure: odds ratio [OR]; hazard ratio [HR]; incidence rate ratio [IRR]; prevalence ratio [PR]

Adjustment:
a) = univariate analysis;
b) = multivariate analysis; adjustment not reported;
c) = adjustment not reported;
d) = final model (age, gender, BMI, smoking status, personal, psychosocial, and work organizational variables);
e) = adjusted for age and gender;
f) = adjusted for age and combined physical work exposure including physical exertion and elbow movements;
g) = sex-adjusted;
h) = fully adjusted for body mass index, pack-years of smoking (continuous), alcohol consumption (continuous), side-specific fractures (never/ever), full anaesthesia within a 5-year period up to the index year (no/yes), predisposing disorders (no/yes), use of crutches within a 5-year period up to the index year (no/yes), hand–arm intensive sports (0, 1, 2) and weight loss ≥ 10 kg within half a year during a 5-year period up to the index year (no/yes) and all occupational exposure variables in table 2 of Svendsen et al. (2012) [39];
i) = adjusted for age (continuous), gender, BMI (continuous);
j) = partly adjusted for body mass index, pack-years of smoking (continuous), alcohol consumption (continuous), side-specific fractures (never/ever), full anaesthesia within a 5-year period up to the index year (no/yes), predisposing disorders (no/yes), use of crutches within a 5-year period up to the index year (no/yes), hand–arm intensive sports (0, 1, 2) and weight loss ≥ 10 kg within half a year during a 5-year period up to the index year (no/yes);
k) = multivariate analyses; adjusted for vitality, white/blue collar, age in four age bands and sex;
l) = adjusted for individual characteristics, repetition, combined physical work exposure including physical exertion, elbow flexion/extension and wrist bending, and social support
m) = adjusted for individual characteristics, repetition, combined physical work exposure including physical exertion, elbow flexion/extension and wrist bending, and social support with aggregation of low categories for combined physical work exposure;
n) = adjusted for age and repetitiveness;

Significance: / = *non-significant;** $p < 0.05$; ** $p < 0.01$; *** $p < 0.001$

File VIII – Table S6: Overview of relevant physical risk factors non-significantly associated with the development of specific disorders at the elbow

Table S6. Overview of relevant physical risk factors non-significantly associated with the development of specific disorders at the elbow.

Exposure (main- and sub-category (S)*)			Non-Significant risk factor specification	Reference	Outcome	Gender	Measure (95% - CI)		Adjustment
Force	S2 Forceful exertion	#46	Hand in forceful grip on average 1 to 2 h/day	[36]	LE	-	OR	1.30 (0.40, 4.20)	a)
					ME	-	OR	2.10 (0.60, 7.20)	a)
					LE / ME	-	OR	1.70 (0.60, 4.50)	a)
		#47	Hand in forceful grip on average 2 to 4 h/day	[36]	LE	-	OR	1.50 (0.50, 4.30)	a)
					ME	-	OR	1.90 (0.50, 6.50)	a)
					LE / ME	-	OR	1.50 (0.60, 4.00)	a)
		#2	Hand in forceful grip on average ≥ 4 h/day	[36]	LE	-	OR	1.70 (0.70, 4.00)	a)
		#48	at 1% Muscular activity in [%/%MVE]	[34]	LE	-	PR	-0.34 (-1.10, 0.50)	g)
		#49	at 15% Muscular activity in [%/%MVE]	[34]	LE	-	PR	-0.02 (-0.20, 0.10)	g)
					ME	-	PR	0.03 (-0.09, 0.10)	g)
	S4 Manual load handling	#50	Lifting (power grip: ≥ 4.5 kg; pinch grip: ≥ 0.9 kg) ≥ 3% time	[37]	LE	-	HR	1.28 (0.76, 2.15)	a)
		#51	Constant moving, lifting and carrying of loads (> 10 kg)	[40]	LE	Men	OR	2.20 (0.80, 6.10)	g)
		#52	Constant moving, lifting and carrying of loads (> 5 kg)	[40]	LE	Women	OR	4.00 (0.90, 27.10)	g)
		#53	Forceful lifting (≥ 4.5 kg) < 0 to < 2 times/min	[31]	LE	-	OR	2.30 (0.95, 5.59)	i)
Repetition	S6 Repetitiveness	#9	Doing repetitive tasks ≥ 4 h/day	[32]	LE	Men	OR	1.05 (0.54, 2.02)	l)
					LE	Women	OR	1.80 (0.91, 3.59)	l)
			at baseline investigation)	[38]	LE	Men	IRR	1.20 (0.40, 3.60)	n)
					LE	Women	IRR	2.70 (1.00, 7.00)	n)
			(at follow up investigation)		LE	Men	IRR	1.20 (0.40, 3.50)	n)
					LE	Women	IRR	1.00 (0.30, 3.80)	n)
			(at baseline and at follow up investigation)		LE	Women	IRR	2.20 (0.80, 6.30)	n)
		#54	Duty cycle ≥ 10% time	[37]	LE	-	HR	1.43 (0.84, 2.43)	a)
		#55	Frequency of shoulder movement ≤ 10 to < 20 times/min	[31]	LE	-	OR	2.03 (0.73, 5.66)	i)

		#56	Frequency of shoulder movement ≥ 20 times/min	[31]	LE	-	OR	2.70 (0.96, 7.63)	i)
		#10	Wrist angular velocity (5°/s) in [%/(°/s)]	[34]	LE	-	PR	0.00 (-0.08, 0.08)	g)
		#57	Repetitive elbow or wrist movements (≥ 4/min) > 0 to < 2.5 h/day, excluding computer use	[39]	UN	-	OR	0.85 (0.51, 1.41)	j)
						-			
Posture/movement	S7 Overhead work	#12	Overhead working > 1h/day	[40]	LE	Women	OR	1.00 (0.30, 4.20)	g)
	S8 Hand movements	#58	Frequent wrist bending or twisting on average 1 to 2 h/day	[36]	LE	-	OR	0.80 (0.10, 7.40)	a)
					LE / ME	-	OR	2.50 (0.60, 11.40)	a)
		#13	Frequent wrist bending or twisting on average 2 to 4 h/day	[36]	LE	-	OR	2.80 (0.70, 10.50)	a)
		#59	Wrist radial deviation < 5° or ulnar deviation ≥ 20° ≥ 4% of time	[31]	LE	-	OR	0.62 (0.32, 1.22)	i)
		#60	Wrist flexion/extension ≥ 15° for ≥ 40% time	[37]	LE	-	HR	0.94 (0.56, 1.58)	a)
		#61	Wrist flexion/extension ≥ 45° for ≥ 2% time	[37]	LE	-	HR	0.98 (0.58, 1.66)	a)
		#16	Wrist flexion (- 40.0°) in [%/°]	[34]	ME	-	PR	0.17 (-0.02, 0.40)	g)
		#17	Wrist flexion (- 20.0°) in [%/°]	[34]	LE	-	PR	0.04 (-0.07, 0.20)	g)
		#62	Wrist flexion (0.0°) in [%/°]	[34]	LE	-	PR	0.05 (-0.06, 0.20)	g)
					ME	-	PR	0.05 (-0.02, 0.10)	g)
		#63	Wrist flexion or extension ≥ 45° ≥ 1 % of time	[31]	LE	-	OR	0.66 (0.34, 1.27)	i)
		#19	Wrist flexion > 1 h/day	[40]	LE	Women	OR	1.20 (0.40, 3.60)	g)
	S9 Forearm & elbow movements	#66	Arm holding in front of body > 1h/day	[40]	LE	Men	OR	1.40 (0.40, 5.00)	g)
					LE	Women	OR	2.20 (0.50, 9.20)	g)
		#67	Swinging movements with the arm > 1h/day	[40]	LE	Men	OR	2.60 (0.80, 8.80)	g)
					LE	Women	OR	1.90 (0.40, 8.30)	g)
		#68	Forearm rotating (also twisting, or screwing motion) 1 to 2 h/day	[36]	LE	-	OR	1.00 (0.20, 4.60)	a)
					ME	-	OR	0.50 (0.10, 3.90)	a)
					LE / ME	-	OR	1.00 (0.30, 3.60)	a)
		#69	Forearm rotating (also twisting, or screwing motion) 2 to 4 h/day	[36]	LE	-	OR	2.30 (0.80, 6.70)	a)
					ME	-	OR	2.80 (1.00, 7.70)	a)
					LE / ME	-	OR	2.60 (1.10, 6.30)	a)
		#22	Forearm rotating (also twisting, or screwing motion) ≥ 4 h/day	[36]	ME	-	OR	2.50 (1.00, 5.80)	a)
	S10 Non- neutral posture	#70	Non-neutral posture (elbow flexion > 100°, or ≥ near maximal pronation/supination; or wrist deviation (> 5° radial, > 10° ulnar) or > 15° palmar/dorsal flexion) ≥ 1 to < 2 h/day	[39]	UN	-	OR	1.29 (0.82, 2.02)	j)
		#71	Wrist flexion/extension ≥ 15° for ≥ 40% time and duty cycle ≥ 10% time	[37]	LE	-	HR	1.30 (0.66, 2.54)	a)

		#72	Wrist flexion/extension ≥ 15° for ≥ 40% time and duty cycle < 10% time	[37]	LE	-	HR	0.68 (0.33, 1.43)	a)
		#73	Wrist flexion/extension ≥ 15° for < 40% time and duty cycle ≥ 10% time	[37]	LE	-	HR	0.99 (0.45, 2.20)	a)
		#74	Wrist flexion/extension ≥ 45° for ≥ 5% time and duty cycle ≥ 10% time	[37]	LE	-	HR	1.22 (0.60, 2.50)	a)
		#75	Wrist flexion/extension ≥ 45° for ≥ 5% time and duty cycle < 10% time	[37]	LE	-	HR	1.00 (0.47, 2.11)	a)
		#76	Wrist flexion/extension ≥ 45° for < 5% time and duty cycle ≥ 10% time	[37]	LE	-	HR	2.06 (0.91, 4.66)	a)
		#77	Forearm pronation ≥ 45° for ≥ 40% time and duty cycle < 10% time	[37]	LE	-	HR	1.61 (0.76, 3.39)	e)
		#78	Forearm pronation ≥ 45° for < 40% time and duty cycle ≥ 10% time	[37]	LE	-	HR	1.76 (0.85, 3.62)	e)
		#64	Forearm pronation ≥ 45° for ≥ 40% time	[37]	LE	-	HR	1.60 (0.93, 2.73)	a)
		#26	Forearm supination ≥ 45° for ≥ 5% time	[37]	LE	-	HR	1.19 (0.71, 2.00)	a)
		#79	Forearm supination ≥ 45° for ≥ 5% time and duty cycle ≥ 10% time	[37]	LE	-	HR	1.47 (0.74, 2.93)	a)
		#80	Forearm supination ≥ 45° for ≥ 5% time and duty cycle < 10% time	[37]	LE	-	HR	1.59 (0.76, 3.34)	a)
		#81	Forearm supination ≥ 45° for < 5% time and duty cycle ≥ 10% time	[37]	LE	-	HR	2.02 (0.98, 4.13)	a)
		#65	Forearm rotation ≥ 45° for ≥ 45% time	[37]	LE	-	HR	1.41 (0.82, 2.42)	a)
		#82	Forearm rotation ≥ 45° for ≥ 45% time and duty cycle < 10% time	[37]	LE	-	HR	2.20 (0.77, 6.30)	a)
		#83	Forearm rotation ≥ 45° for < 45% time and duty cycle ≥ 10% time	[37]	LE	-	HR	2.22 (0.70, 7.04)	a)
	S11 Body posture	#84	Standing work > 1h/day	[40]	LE	Men	OR	2.40 (0.80, 6.70)	g)
					LE	Women	OR	1.90 (0.40, 8.10)	g)
		#85	Sedentary work > 1h/day	[40]	LE	Men	OR	0.50 (0.20, 1.10)	g)
					LE	Women	OR	0.50 (0.20, 2.10)	g)
		#86	PC work > 1h/day	[40]	LE	Men	OR	1.00 (0.30, 3.50)	g)
					LE	Women	OR	0.30 (0.10, 0.80)	g)
Vibration	S12 Hand-arm vibration	#87	Hand-arm vibration: acceleration ≥ 3 m/s² > 0 to < 1 h/day	[39]	UN	-	OR	1.97 (0.95, 4.10)	j)
		#88	Use of vibrating hand tools > 2 h/day	[32]	LE	Men	OR	0.95 (0.46, 1.97)	a)
					LE	Women	OR	2.08 (0.62, 6.98)	a)
		#89	Vibration stress > 1h/day	[40]	LE	Men	OR	1.50 (0.50, 4.10)	g)
	S13 Force & repetition	#90	Frequency of forceful exertions (≥ 44.1 N or ≥ 4.5 kg) ≥ 2 times/min	[37]	LE	-	HR	1.18 (0.69, 2.00)	a)
	S14 Posture & force	#91	Forearm supination ≥ 45°; forceful lifting (≥ 4.5 kg) (low on either posture or force and high on the other) in [% time]	[31]	LE	-	OR	1.21 (0.44, 3.38)	i)
		#92	Forearm supination ≥ 45° ≥ 5 % (duty cycle) or forceful lifting (≥ 4.5 kg) > 0% of time	[31]	LE	-	OR	1.09 (0.38, 3.09)	d)

Combined Exposures	#93	Wrist flexion/extension ≥ 15° for ≥ 40% time and any power grip (≥ 44.1 N)	[37]	LE	-	HR	1.52 (0.78, 2.96)	a)
	#94	Wrist flexion/extension ≥ 15° for ≥ 40% time and no power grip (≥ 44.1 N)	[37]	LE	-	HR	0.77 (0.40, 1.50)	a)
	#95	Wrist flexion/extension ≥ 15° for < 40% time and any power grip (≥ 44.1 N)	[37]	LE	-	HR	1.32 (0.55, 3.15)	a)
	#96	Wrist flexion/extension ≥ 15° for ≥ 40% time and lifting (≥ 4.5 kg) ≥ 3% time	[37]	LE	-	HR	1.18 (0.60, 2.33)	a)
	#97	Wrist flexion/extension ≥ 15° for ≥ 40% time and lifting (≥ 4.5 kg) < 3% time	[37]	LE	-	HR	0.74 (0.36, 1.50)	a)
	#98	Wrist flexion/extension ≥ 15° for < 40% time and lifting (≥ 4.5 kg) ≥ 3% time	[37]	LE	-	HR	0.96 (0.43, 2.13)	a)
	#99	Wrist flexion/extension ≥ 45° for ≥ 5% time and any power grip (≥ 44.1 N)	[37]	LE	-	HR	1.39 (0.67, 2.89)	a)
	#100	Wrist flexion/extension ≥ 45° for ≥ 5% time and no power grip (≥ 44.1 N)	[37]	LE	-	HR	0.91 (0.46, 1.79)	a)
	#101	Wrist flexion/extension ≥ 45° for < 5% time and any power grip (≥ 44.1 N)	[37]	LE	-	HR	1.94 (0.85, 4.39)	a)
	#102	Wrist flexion/extension ≥ 45° for ≥ 5% time and lifting (≥ 4.5 kg) ≥ 3% time	[37]	LE	-	HR	1.11 (0.54, 2.27)	a)
	#103	Wrist flexion/extension ≥ 45° for ≥ 5% time and lifting (≥ 4.5 kg) < 3% time	[37]	LE	-	HR	0.93 (0.45, 1.95)	a)
	#104	Wrist flexion/extension ≥ 45° for < 5% time and lifting (≥ 4.5 kg) ≥ 3% time	[37]	LE	-	HR	1.52 (0.68, 3.43)	a)
	#105	Forearm pronation ≥ 45° for ≥ 40 % time and no power grip (≥ 44.1 N)	[37]	LE	-	HR	1.97 (1.00, 3.89)	e)
	#106	Forearm pronation ≥ 45° for < 40 % time and any power grip (≥ 44.1 N)	[37]	LE	-	HR	1.65 (0.69, 3.96)	e)
	#107	Forearm pronation ≥ 45° for ≥ 40 % time and lifting (≥ 4.5 kg) < 3 % of time	[37]	LE	-	HR	1.98 (0.96, 4.07)	e)
	#108	Forearm pronation ≥ 45° for < 40 % time and lifting (≥ 4.5 kg) ≥ 3 % of time	[37]	LE	-	HR	1.58 (0.77, 3.22)	e)
	#109	Forearm supination ≥ 45° for ≥ 5% time and any power grip (≥ 44.1 N)	[37]	LE	-	HR	1.48 (0.62, 3.55)	a)
	#110	Forearm supination ≥ 45° for ≥ 5% time and no power grip (≥ 44.1 N)	[37]	LE	-	HR	1.86 (0.96, 3.60)	a)
	#111	Forearm supination ≥ 45° for ≥ 5% time and lifting (≥ 4.5 kg) ≥ 3% time	[37]	LE	-	HR	1.32 (0.66, 2.62)	a)
	#112	Forearm supination ≥ 45° for ≥ 5% time and lifting (≥ 4.5 kg) < 3% time	[37]	LE	-	HR	1.89 (0.92, 3.90)	a)
	#113	Forearm rotation ≥ 45° for ≥ 45% time and no power grip (≥ 44.1 N)	[37]	LE	-	HR	1.88 (0.83, 4.28)	a)
	#114	Forearm rotation ≥ 45° for < 45% time and any power grip (≥ 44.1 N)	[37]	LE	-	HR	2.31 (0.82, 6.53)	a)
	#115	Forearm rotation ≥ 45° for ≥ 45 % time and lifting (≥ 4.5 kg) ≥ 3 % of time	[37]	LE	-	HR	2.27 (0.88, 5.88)	a)
	#116	Forearm rotation ≥ 45° for ≥ 45 % time and lifting (≥ 4.5 kg) < 3 % of time	[37]	LE	-	HR	1.50 (0.58, 3.84)	a)
	#117	Forearm rotation ≥ 45° for < 45 % time and lifting (≥ 4.5 kg) ≥ 3 % of time	[37]	LE	-	HR	1.25 (0.43, 3.61)	a)
	#40	Forceful exertion (turning) > 1 h/day	[40]	LE	Women	OR	1.40 (0.30, 6.80)	g)
	#45	Less hard physical exertion (BORG Score 6 to 13) and	[32]	LE	Men	OR	1.52 (0.52, 4.44)	l)
		1 to 2 elbow movements		LE	Women	OR	0.53 (0.17, 1.60)	l)
	#118	Hard physical exertion (BORG Score 14 to 20) and no elbow movement	[32]	LE	Men	OR	0.56 (0.11, 2.92)	a)
				LE	Men	OR	0.76 (0.14, 3.98)	l)
				LE	Women	OR	0.80 (0.24, 2.71)	l)
	#41	Hard physical exertion (BORG Score 14 to 20) and 1 elbow movement	[32]	LE	Women	OR	2.54 (1.12, 5.76)	m)
	#42	Hard physical exertion (BORG Score 14 to 20) and 2 elbow movements	[32]	LE	Men	OR	5.60 (2.76, 11.35)	m)

			(elbow movements = elbow flexion/extension > 2 h/day and wrist bending > 2 h/day		LE	Women	OR	2.89 (1.28, 6.51)	m)
			High physical exertion with elbow flexion/ extension > 2 hours/day wrist and extreme bending > 2 hours/day (at baseline investigation)	[38]	LE	Women	IRR	1.50 (0.50, 4.70)	f)
	S15 Repetition & posture	#43	Repetitive/constrained work with > 30 s or > 50 % of cycle time involved same fundamental cycle) vs. > 50% (working time) involved prolonged awkward postures	[33]	LE	Men	PR	1.00 (0.30, 2.80)	c)
					LE	Women	PR	1.90 (1.00, 3.80)	c)
					ME	Women	PR	3.50 (1.00, 12.00)	c)
					Pronator	Women	PR	3.40 (0.40, 29.00)	c)
					Radial	Men	PR	1.30 (0.10, 21.00)	c)
					Radial	Women	PR	3.40 (0.80, 15.00)	c)
	S16 Posture & repetition & force	#119	Wrist flexion/extension ≥ 15° for ≥ 40% time and forceful exertions (≥ 44.1 N) ≥ 2/min	[37]	LE	-	HR	1.09 (0.56, 2.13)	a)
		#120	Wrist flexion/extension ≥ 15° for ≥ 40% time and forceful exertions (≥ 44.1 N) < 2/min	[37]	LE	-	HR	0.67 (0.33, 1.36)	a)
		#121	Wrist flexion/extension ≥ 15° for < 40% time and forceful exertions (≥ 44.1 N) ≥ 2/min	[37]	LE	-	HR	0.77 (0.34, 1.74)	a)
		#122	Wrist flexion/extension ≥ 45° for ≥ 5% time and forceful exertions (≥ 44.1 N) ≥ 2/min	[37]	LE	-	HR	1.02 (0.51, 2.04)	a)
		#123	Wrist flexion/extension ≥ 45° for ≥ 5% time and forceful exertions (≥ 44.1 N) < 2/min	[37]	LE	-	HR	0.86 (0.41, 1.77)	a)
		#124	Wrist flexion/extension ≥ 45° for < 5% time and forceful exertions (≥ 44.1 N) ≥ 2/min	[37]	LE	-	HR	1.28 (0.55, 3.02)	a)
		#125	Forearm pronation ≥ 45° for ≥ 40% time and forceful exertions (≥ 44.1 N) ≥ 2/min	[37]	LE	-	HR	2.28 (1.00, 5.19)	a)
		#126	Forearm pronation ≥ 45° for ≥ 40% time and forceful exertions (≥ 44.1 N) < 2/min	[37]	LE	-	HR	1.36 (0.63, 2.94)	a)
		#127	Forearm pronation ≥ 45° for < 40% time and forceful exertions (≥ 44.1 N) ≥ 2/min	[37]	LE	-	HR	1.03 (0.44, 2.42)	a)
		#128	Forearm supination ≥ 45° for ≥ 5% time and forceful exertions (≥ 44.1 N) ≥ 2/min	[37]	LE	-	HR	1.29 (0.66, 2.51)	a)
		#129	Forearm supination ≥ 45° for ≥ 5% time and forceful exertions (≥ 44.1 N) < 2/min	[37]	LE	-	HR	1.36 (0.65, 2.82)	a)

		#130	Forearm supination ≥ 45° for < 5% time and forceful exertions (≥ 44.1 N) ≥ 2/min	[37]	LE	-	HR	1.35 (0.64, 2.83)	a)
		#131	Forearm rotation ≥ 45° for ≥ 45% time and forceful exertions (≥ 44.1 N) ≥ 2/min	[37]	LE	-	HR	1.96 (0.80, 4.84)	a)
		#132	Forearm rotation ≥ 45° for ≥ 45% time and forceful exertions (≥ 44.1 N) < 2/min	[37]	LE	-	HR	1.52 (0.63, 3.66)	a)
		#133	Forearm rotation ≥ 45° for < 45% time and forceful exertions (≥ 44.1 N) ≥ 2/min	[37]	LE	-	HR	1.20 (0.42, 3.37)	a)

Legend: * Forceful exertions were only shown for power grip, details for pinch grip were shown in Supplementary File VII (Table S5); sub-categories of exposure S, S3 and S5 are significant and are not presented here.
Outcome: UN = ulnar neuropathy; LE = lateral epicondylitis; ME = medial epicondylitis; LE / ME = lateral and / or medial epicondylitis, Radial = Radial tunnel syndrome, Pronator = Pronator teres syndrome
Measure: odds ratio [OR]; hazard ratio [HR]; incidence rate ratio [IRR]; prevalence ratio [PR]
Adjustment:
a) = univariate analysis;
c) = adjustment not reported;
d) = final model (age, gender, BMI, smoking status, personal, psychosocial, and work organizational variables);
e) = adjusted for age and gender;
f) = adjusted for age and combined physical work exposure including physical exertion and elbow movements;
g) = sex-adjusted;
i) = adjusted for age (continuous), gender, BMI (continuous);
j) = partly adjusted for body mass index, pack-years of smoking (continuous), alcohol consumption (continuous), side-specific fractures (never/ever), full anaesthesia within a 5-year period up to the index year (no/yes), predisposing disorders (no/yes), use of crutches within a 5-year period up to the index year (no/yes), hand–arm intensive sports (0, 1, 2) and weight loss ≥ 10 kg within half a year during a 5-year period up to the index year (no/yes);
l) = adjusted for individual characteristics, repetition, combined physical work exposure including physical exertion, elbow flexion/extension and wrist bending, and social support
m) = adjusted for individual characteristics, repetition, combined physical work exposure including physical exertion, elbow flexion/extension and wrist bending, and social support with aggregation of low categories for combined physical work exposure;
n) = adjusted for age and repetitiveness
